# ﻿An annotated catalogue of selected historical type specimens, including genetic data, housed in the Natural History Museum Vienna

**DOI:** 10.3897/zookeys.1203.117699

**Published:** 2024-05-30

**Authors:** Anja Palandačić, Min J. Chai, Gennadiy A. Shandikov, Nesrine Akkari, Pedro R. Frade, Susanne Randolf, Hans-Martin Berg, Ernst Mikschi, Nina G. Bogutskaya

**Affiliations:** 1 Fish collection, First Zoological Department, Natural History Museum Vienna, Burgring 7, 1010 Vienna, Austria; 2 Department of Biology, Biotechnical Faculty, University of Ljubljana, Jamnikarjeva 101, SI-1000 Ljubljana, Slovenia; 3 Myriapoda collection, Third Zoological Department, Natural History Museum Vienna, Burgring 7, 1010 Vienna, Austria; 4 Evertebrata Varia collection, Third Zoological Department, Natural History Museum Vienna, Burgring 7, 1010 Vienna, Austria; 5 Neuropterida-Orthopteroidea-Insecta Varia collection, Second Zoological Department, Natural History Museum Vienna, Burgring 7, 1010 Vienna, Austria; 6 Bird collection, First Zoological Department, Natural History Museum Vienna, Burgring 7, 1010 Vienna, Austria; 7 BIOTA j d.o.o., Dolga Gora 2, 3232 Ponikva, Slovenia

**Keywords:** Biodiversity, digitisation, historical DNA, type locality Vienna, zoological collections

## Abstract

Museum collections are an important source for resolving taxonomic issues and species delimitation. Type specimens as name-bearing specimens, traditionally used in morphology-based taxonomy, are, due to the progress in historical DNA methodology, increasingly used in molecular taxonomic studies. Museum collections are subject to constant deterioration and major disasters. The digitisation of collections offers a partial solution to these problems and makes museum collections more accessible to the wider scientific community. The Extended Specimen Approach (ESA) is a method of digitisation that goes beyond the physical specimen to include the historical information stored in the collection. The collections of the Natural History Museum Vienna represent one of the largest non-university research centres in Europe and, due to their size and numerous type specimens, are frequently used for taxonomic studies by visiting and resident scientists. Recently, a version of ESA was presented in the common catalogue of the Fish and Evertebrata Varia collections and extended to include genetic information on type specimens in a case study of a torpedo ray. Here the case study was extended to a heterogeneous selection of historical type series from different collections with the type locality of Vienna. The goal was to apply the ESA, including genetic data on a selected set of type material: three parasitic worms, three myriapods, two insects, twelve fishes, and one bird species. Five hundred digital items (photographs, X-rays, scans) were produced, and genetic analysis was successful in eleven of the 21 type series. In one case a complete mitochondrial genome was assembled, and in another case ten short fragments (100–230 bp) of the cytochrome oxidase I gene were amplified and sequenced. For five type series, genetic analysis confirmed their taxonomic status as previously recognised synonyms, and for one the analysis supported its status as a distinct species. For two species, genetic information was provided for the first time. This catalogue thus demonstrates the usefulness of ESA in providing digitised data of types that can be easily made available to scientists worldwide for further study.

## ﻿Introduction

Museum collections are the largest archives of biodiversity, encompassing taxonomic, spatial, and temporal variation (Webster 2017). As such, they are an important source for studying demographic (Hoeksema and Koh 2009; van der Meij et al. 2009; Hoeksema et al. 2011; Meineke et al. 2018) and climatic changes (van der Meij et al. 2010; Robbirt et al. 2014), as well as for resolving taxonomic questions (Stoev et al. 2013; Silva et al. 2017, 2019; Antić and Akkari 2020; Kehlmaier et al. 2020; Straube et al. 2021) and the species delimitation (Agne et al. 2022a, 2022b) of (sometimes) extinct species (Feigin et al. 2017; Palandačić et al. 2023). Type specimens as name-bearing specimens, traditionally used in morphology-based taxonomy (Maxted 1992; Winston 1999), are, due to the progress in historical DNA (as defined in Raxworthy and Smith 2021) methodology, increasingly used in molecular taxonomy studies (e.g., Federhen 2014; Li et al. 2015; Straube et al. 2021; Agne et al. 2022a; Sullivan et al. 2022). While barcoding projects have provided a method for rapid species identification (Goldstein and DeSalle 2011; Kress and Erickson 2012), only genotyping of the type specimen(s) provides an explicit link between a genetic lineage (or in some cases a specific sequence) and the species name (Prosser et al. 2016; Castañeda-Rico et al. 2022). Nevertheless, there is often a (taxonomic) ambiguity associated with the type series and the specimens it contains (e.g., van Steenberge et al. 2016; Agatha et al. 2021), and therefore a careful examination of the associated historical information should be carried out in order to contextualise the acquired genetic data appropriately (Durette-Desset and Digiani 2010; Renner 2016; Kehlmaier et al. 2020).

Museum collections are subject to gradual but constant deterioration, as well as catastrophes of major proportions (recently reviewed in Tyler et al. (2023)). The digitisation of collections offers a partial solution to these problems and, although digital data can never replace the physical specimen, it can be seen as an insurance policy. At the same time, through online databases or other shared resources (Lendemer et al. 2020; Monfils et al. 2022; Hardisty et al. 2023), digitisation makes museum collections more accessible to the wider scientific community and to researchers from disadvantaged or distant countries who may not have the opportunity to see the specimen in person (open science concept). The Extended Specimen Approach (ESA; Webster 2017; Lendemer et al. 2020) is a method of digitisation that goes beyond the physical specimen, e.g., photographs, X-rays, CT scans (Stoev et al. 2013; Akkari et al. 2015, 2018), but also includes all its attributes, such as historical information stored in the collection in the form of acquisition and inventory books, inventory cards and labels (Haston et al. 2012; Albano et al. 2018; Price et al. 2018; Zahiri et al. 2021; Bogutskaya et al. 2022; Takano et al. 2024).

**Figure 1.**
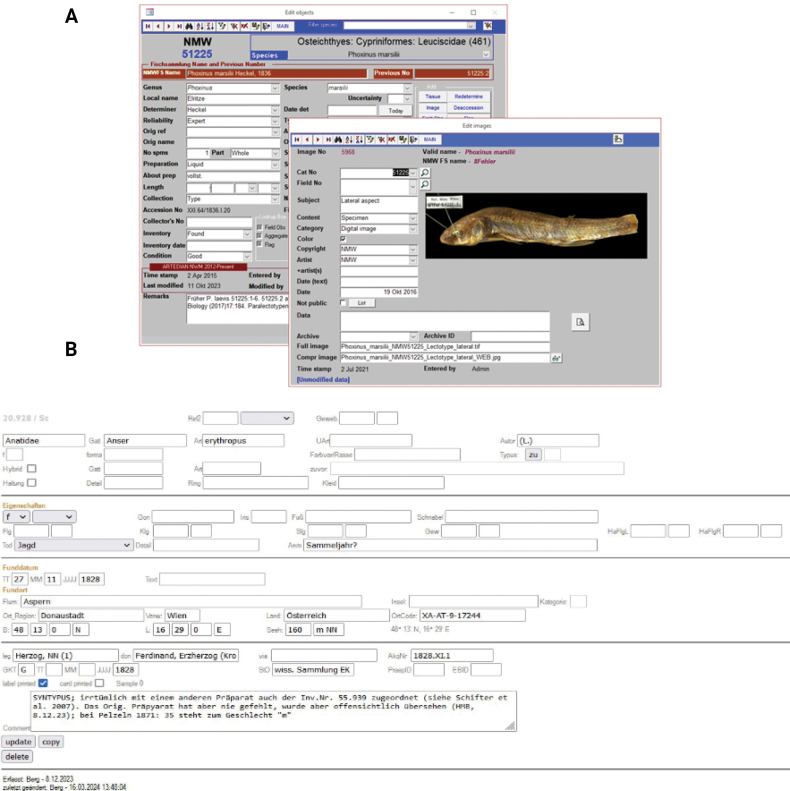
**A** an example of the Fish collection and **B** bird collection databases presenting lectotype of *Phoxinusmarsilii* Heckel, 1836 and *Anserbrevirostris* Brehm, 1831.

Founded more than 270 years ago, the collections of the Natural History Museum Vienna (NHMW) represent one of the largest non-university research centres in Europe, with both historic and recent specimens of most animal groups. The collections date back to the United Imperial Royal Natural History Cabinet of the early 18^th^ century and are the result of many expeditions and material collected by naval personnel on special missions, as well as many specimens donated, purchased, or exchanged (Kähsbauer 1959; Fischer et al. 1976; Hamann 1976; Schefbeck 1996; Herzig-Straschil 1997). Due to their size and extensive representation of type specimens, the collections are repeatedly used in taxonomic studies by visiting and resident scientists. Currently, the collections are in various stages of digitisation (e.g., Fig. 1) and a museum-wide database covering all NHMW collections is being developed, but none of the collections are yet available online. Thus, the holdings of the museum have been reviewed in a series of 20 volumes of Catalogues of the scientific collections of the Natural History Museum Vienna (Kataloge der wissenschaftlichen Sammlungen des Naturhistorischen Museums in Wien) published 1978–2007, and are regularly presented in illustrated and annotated catalogues of different taxonomic groups (e.g., Wirkner et al. 2002; Schileyko and Stagl 2004; Stagl and Stoev 2005; Stagl and Zapparoli 2006; Schifter et al. 2007; Ilie et al. 2009; Zettel et al. 2022, 2023; van den Elzen et al. 2023), by collectors or authors (e.g., Albano et al. 2018), or only type specimens by taxa or/and authors (e.g., Saint Quentin 1970; Schifter 1991; Gemel et al. 2019).

Similarly, a version of this approach was presented in the common catalogue of the NHMW Fish and Evertebrata Varia collections published recently (Bogutskaya et al. 2022), and extended to include genetic information on type specimens in a case study of a torpedo ray (Palandačić et al. 2023). Here, this case study has been extended to heterogeneous selection of historical type series from different collections, with the common denominator of the type locality Vienna. Thus, the goal of this catalogue is to apply the ESA as presented earlier (digitisation of physical specimens, associated historical information; Bogutskaya et al. (2022)) and including genetic data (where possible; see Palandačić et al. (2023: fig. 1)), on a wide variety of type material: three parasitic-worm, three myriapod, two insect, twelve fish, and one bird species. The catalogue includes historical information and literature in which they were mentioned, as well as genetic data where the analysis was successful.

### ﻿Background on the authors of the species names

#### ﻿Chromadorea and Trematoda type series

The Chromadorea and Trematoda type series represented in this catalogue are a part of the Parasitic-worms collection (for further reading see Sattmann et al. (2000, 2001), Sattmann (2002), Stagl and Sattmann (2013)), which is a part of the larger Evertebrata Varia (EV) collection, and were described by Maximilian Braun, Leopold Karl Böhm, and Rudolf Supperer.

Maximilian Braun (1850–1930) was an ornithologist, zoologist, and physician whose main focus was on the trematode parasites of birds. As an anatomist, Braun contributed greatly to the medical field of parasitology. Born in Myslowitz in 1850, Braun studied medicine and natural sciences before obtaining his doctorate in 1877. Braun was a full Professor of zoology and comparative anatomy at the University of Rostock and later in Königsberg (now Kaliningrad), where he was director of the Zoological Museum and where he would die in 1930. In 1916–1917 he was president of the German Zoological Society. During his career, Maximilian Braun published several zoological books, such as Developmental history of the Tapeworm (Entwicklungsgeschichte des Bandwurms) (Braun 1883), and also on parasitology, such as A handbook of Practical Parasitology (Ein Handbuch der praktischen Parasitologie) (Braun and Lühe 1910).

Leopold Karl Böhm (1886–1958) was a veterinarian, zoologist, and parasitologist. Born in Vienna in 1886, Böhm received his doctorate in 1910 and his veterinary degree in 1916, becoming an associate Professor in 1924 and a Professor in 1937. Böhm went on to head the Institute of General Zoology and Parasitology at the University of Veterinary Medicine in Vienna, and later serving as its rector (1948–1950) and vice-rector (1950–1952). In 1941, Böhm became a full member of the Austrian Academy of Sciences and died in Vienna in 1958. Böhm published numerous scientific papers in zoological and veterinary journals and was co-editor of several journals (e.g., the Vienna Veterinary Monthly (Wiener Tierärztliche Monatsschrift), the Journal of Scientific Biology (Zeitschrift für wissenschaftliche Biologie), the Journal of Parasitology (Zeitschrift für Parasitenkunde), and the Austrian Zoological Journal (Österreichische Zoologische Zeitschrift).

Rudolf Supperer (1918–2006) was a veterinary surgeon and student of Leopold Karl Böhm, who later became Professor of Parasitology and General Zoology at the University of Veterinary Medicine in Vienna. Born in Kirchstetten in 1918, Supperer was also rector of the University of Veterinary Medicine from 1967 to 1969. Together with Böhm, Supperer established the genus name *Wehrdikmansia*, in honour of the work of Wehr and Dikmans, who described numerous filarioid nematodes (family Filariidae; Wehr and Dikmans (1935)) associated with various diseases of North American mammals such as sheep, deer, or elk.

#### ﻿Diplopoda type series

The Diplopoda type series represented in this catalogue are a part of Myriapoda collection (MY; for further reading see Stagl 2006) and were described by Robert Latzel and Carl Attems.

Robert Latzel (1845–1919), a pioneer in myriapodology, was born in Silesia (today’s Czech Republic). Although his main profession at that time was a teacher of natural history at high schools and later a principal of the main grammar school in Klagenfurt, Carinthia, since 1875, he studied myriapods. His major work (Latzel 1884) was considered a turning point in millipede systematics, as he was the first to emphasize on the importance of the gonopods (modified legs used for copulation in millipedes) for the taxonomy of the group. Latzel sold large collections to the NHMW in 1884 and in 1919, the year of his death. The collection contained ca 545 species and 8098 specimens. The main issue with these samples is that Latzel did not give precise information on the localities of the species, neither in the original description of new species and variations, nor on the labels in the jars of his collection. This information could however be retrieved only from the book of acquisitions for 1884, written in red ink by Latzel himself (Stagl 2006).

The collection owes its value also to the imminent Austrian myriapodologist Carl Attems (1894–1952), examined material from nearly all parts of the world, described ca 1700 species and published 138 papers, monographs, and textbooks.

#### ﻿Insecta type series

The Insecta type series represented in this catalogue are a part of the Neuropterida-Orthopteroidea-Insecta Varia collection (ORTH; for further reading see Kaltenbach 2001) and were described by Vinzenz Kollar and Hermann Krauss.

Detailed biographical data on Vinzenz Kollar (1797–1860) can be found in von Wurzbach (1864) and in two obituaries by Schiner (1860) and Schrötter (1861). The latter includes a bibliography and dates of birth and death that differ from other sources. Recent publications deal with different aspects of this versatile researcher (Thaler and Gruber 2003; Rabitsch 2006; Christian 2008; Zuna-Kratky 2017). The following is a brief summary of Kollar’s life and work in relation to the insect collection.

Vinzenz Kollar was born in Kranowitz (then Prussian Silesia, now Poland) on 15 January 1797. After completing his education, he moved to Vienna in 1815 to study medicine. His growing interest in entomology led him to the Natural History Cabinet in 1817, where he met the curator of the insect collection, Franz Anton Ziegler (1760–1842). Under his guidance, Kollar began to examine the existing collections and put them into a systematic order. Initially an unpaid volunteer, he was eventually given a permanent position and finally became the director of the Imperial Court Zoological Cabinet in 1851.

His first publication was a systematic work on a genus of beetles, inspired by the many collections made by explorers in Brazil (Kollar 1824). But far more than the diversity of forms, he seems to have been fascinated by the distribution, lifestyle, and development of insects. Over the years, in addition to faunistic works (e.g., Kollar 1831a, 1831b, 1833a, 1850), he published mainly on insects that were directly harmful to humans or indirectly harmful as pests in agriculture and forestry (e.g., Pohl and Kollar 1832; Kollar 1833b, 1837, 1842, 1850, 1855). Kollar’s extensive collecting activities greatly increased the number of known locust species in Upper and Lower Austria. He listed 51 species and described four species new to science (Kollar 1833a). One of them still bears his name in its German common name: Kollars Höhlenschrecke, *Troglophiluscavicola* (Kollar, 1833).

Vinzenz Kollar was a member of the Austrian Academy of Sciences, awarded the Ritterkreuz of the Franz-Joseph Order and appointed a Geheimer Regierungsrat. After his death on 30 May 1860, he was buried in an honorary grave in the Vienna Central Cemetery.

The largest and most valuable addition to the Orthoptera collection was the Brunner von Wattenwyl collection, acquired by the Museum in 1901. At that time, it was one of the most important Orthoptera collections in the world, with some 79,500 specimens of 10,600 species. Carl Brunner von Wattenwyl (1823–1914) was a Swiss geologist who established telegraphy in Switzerland in 1851 and became director of the Austrian Post and Telegraph Administration in 1857 (Brunner 1914). His great passion for Orthoptera led him to start his own collection. As director of telegraphy he was also responsible for the expansion of the telegraph network in south-eastern Europe and Turkey. The collection also includes Orthoptera collected during his business trips to these areas (Directories I–VI of Brunner von Wattenwyl/2^nd^ Zoological Department).

Not only did he collect himself, but he also actively traded and added to his collection through purchases. In 1859 he received from Rudolf Türk some alpine groundhoppers collected on the banks of the Danube. Not much is known about Rudolf Türk. His date of birth is given as “around 1820” (Zuna-Kratky et al. 2009) and his main occupation was probably imperial court secretary (Krauss 1876). In his publications he abbreviated his first name to “Rud.”. Türk summarised the results of his intensive collecting activities in Lower Austria in a detailed fauna listing 78 different species (Türk 1858, 1860, 1862).

The physician and entomologist Hermann Krauss (1848–1939) was in active correspondence with Brunner von Wattenwyl for several decades (Entomological letter collection Brunner von Wattenwyl (Entomologische Briefsammlung Brunner von Wattenwyl)/Second Zoological Department/NHMW) and worked as an assistant at the Natural History Court Museum (k. k. Naturhistorisches Hofmuseum) from 1876 to 1880. Later he returned to Tübingen and opened a medical practice (Kaltenbach 2001). The first description of a new species dates from his time in Vienna, which he named *Tetrixtuerki* in honour of Türk as a collector and for his faunistic works (Krauss 1876). In the description he noted that the species was only found in a few localities at the time and attributed this to the “reorganisation of the whole terrain”. The reorganisation refers to the regulation of the Danube, which began in 1870 and led to the rapid and complete disappearance of the sandy, gravelly, sparsely vegetated alluvial soils so important for this species (Zuna-Kratky et al. 2009).

#### ﻿Actinopteri type series

The Actinopteri type series represented in this catalogue are a part of the Fish collection (FS from Fischsammlung in German; for further reading see Herzig-Straschil 1997; Mikschi 2009) and were described by Johann Jakob Heckel.

A detailed description of the life and scientific career of Johann Jakob Heckel (1790–1857) can be found in historical (Anonymous 1857a; von Wurzbach 1862; Carus 1880) and recent (Herzig-Straschil 1997; Svojtka et al. 2009, 2012) publications. In the following, we present a brief summary of Heckel’s life and activities relevant to the subject of this study.

Johann Jakob Heckel, born on 23 January 1790 in Churpfalz (now Mannheim), began his career in 1818 as a volunteer taxidermist in the United Imperial Royal Natural History Cabinet in Vienna. In 1819–1820, Heckel travelled through Germany, Switzerland, and Italy, and in August 1820 he was officially employed as a taxidermist in the vertebrate department of the Natural History Cabinet in Vienna under the curator Joseph Natterer Jr. He began scientific studies of terrestrial and freshwater molluscs, birds, and fishes, paying particular attention to the Fish collection, which at that time consisted of only ca 700 specimens.

**Figure 2.**
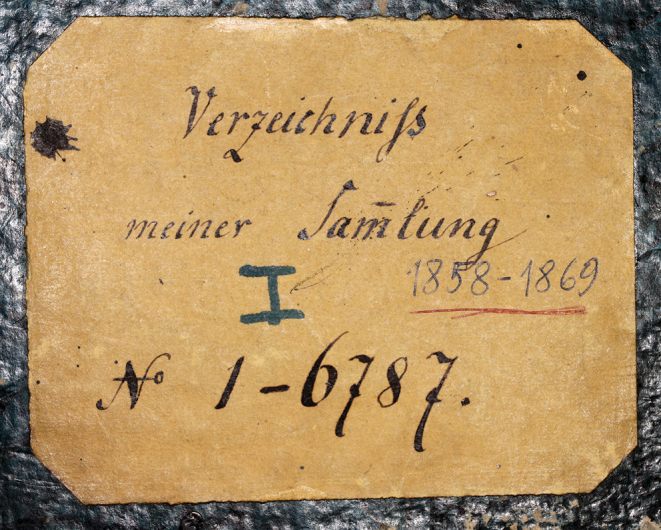
Directory I of the Brunner von Wattenwyl collection.

Under the guidance of the curator Leopold Joseph Fitzinger (1802–1884), Heckel participated in the preparation of a detailed inventory of Austrian fish fauna, first of the Danube, then of Lake Neusiedl, Lake Balaton, and the Upper Austrian lakes. In 1824 he travelled to Upper Austria and Salzburg for several months and made some useful acquaintances, including the well-known Swiss ichthyologist Louis J. R. Agassiz (1807–1873), who then spent a long period in Vienna in 1830. A number of fish specimens collected during these trips are still extant in the NHMW Fish collection (e.g., acquisitions 1824.II; Fig. 2).

On 26 February 1832, Heckel was appointed curator of the Fish collection of the Natural History Cabinet. A number of stuffed fishes collected by him were given to the Vienna University Museum (Fitzinger 1856), the so-called Old Collection (Alte Sammlung); some collections were later returned to the NHMW. In 1835 Heckel was appointed second curator and soon after, in 1836, first curator, responsible for overseeing the incorporation of the collections from the disbanded “Brazilian Museum” and the subsequent rearrangement of the collections in the Natural History Cabinet. In the following years, Heckel also undertook expeditions to southern Hungary and Croatia (in 1839), Dalmatia and western Herzegovina (in August-September 1840, together with Rudolf Kner), and to the Tisza region, which enriched his knowledge, especially of the cyprinoids. These expeditions were extremely productive and a large number of new species were described.

Heckel was a skilled artist; his original drawings of scales, bones, teeth, and whole fishes were used as illustrations in his publications (e.g., Heckel 1843; Heckel and Kner 1857). Over 100 drafts of illustrations, still deposited in the NHMW Archive (in a sketchbook of Heckel, who used an instrument he developed to draw precise outlines), were made by Heckel “with mathematical accuracy using his ichthyometer” (Heckel 1852a: 109: “Sämmtliche Tafeln sinf mittelst meines Ichthyometers mit mathematischer Genaugkeit angefertigt worden“). Heckel was particularly interested in some osteological features of the fishes he studied, such as the scale structure and the pharyngeal bones and teeth of cypriniforms. Many of his new species (including those discussed in this catalogue) were described on the basis of the shape and structure of the pharyngeal teeth. Heckel’s collection of cypriniform pharyngeal bones is still deposited in the NHMW Fish collection and comprises 184 catalogue numbers. Heckel also established a classification of cypriniform fishes based on the structure of the pharyngeal bones and teeth (“Dispositio systematica familiae Cyprinorum”, Heckel 1843: 1013–1043).

During the 1840s and 1850s, Heckel authored or contributed to more than 30 publications on recent fishes (e.g., Heckel 1850, 1851a, 1851c, 1851d, 1852a, 1852b), including the new species descriptions discussed in this catalogue (for a full bibliographic list, see Svojtka et al. (2012)). By this time, his reputation and expertise had brought him into close contact with the most eminent ichthyologists in Europe: Prince Charles Bonaparte, Johannes Müller, Louis Agassiz, and Achille Valenciennes.

In 1851, Emperor Franz Joseph ordered the reorganisation of the United Imperial Royal Natural History Cabinet into three administratively separate cabinets, and Heckel was appointed deputy curator of the Fish collection of the Court Zoological Cabinet. Johann Jakob Heckel died of ‘wasting’ (a long-term infection or tuberculosis) on 1. March 1857. He did not live to see the publication of the summary results on the fishes of Austria (Heckel and Kner 1857).

#### ﻿Aves type series

The two Aves types represented in this catalogue are a part of the Bird collection (VS from Vogelsammlung in German; for further reading see Bauernfeind 2003; Schifter 2010; Berg 2016) and were described by Christian Ludwig Brehm.

Christian Ludwig Brehm (1787–1864) was born on 24 January 1787 in Schönau vor dem Walde near Gotha, Thuringia, the son of a pastor. He studied theology at Jena and began a career as a tutor in 1810. In 1812 he became a pastor in Drackendorf, near Jena, and from 1813 until his death on 23 June 1864 he was the parish priest in Renthendorf, near Neustadt, Thuringia (Hildebrandt 1929; Kleinschmidt 1955; Gebhardt 1964).

Throughout his life, Brehm’s deep interest in the world of birds coexisted with his pastoral duties, earning him an honoured position in German ornithology. His early fascination with birds, coupled with his expertise in taxidermy and bird collecting, culminated in a collection of at least 9000 bird specimens. This collection laid the foundation for his research into the differentiation of bird species. Initiated by Pastor Otto Kleinschmidt and Ernst Hartert, a significant part of Brehm’s collection found its way to the Rothschild Museum in Tring, UK, and then to New York. Some parts of the collection eventually returned to the Alexander Koenig Museum in Bonn. Brehm’s attention to minute morphological distinctions led to several species and subspecies descriptions, most notably in his comprehensive work Handbook of the natural history of all birds in Germany (Handbuch der Naturgeschichte aller Vögel Deutschlands) (Brehm 1831). Despite criticism of his typological taxonomic views, his descriptions of some 60 bird taxa remain valid to day (Hildebrandt 1929; Kleinschmidt 1955; Gebhardt 1964).

Brehm’s other notable works include Contributions to Ornithology (Beiträge zur Ornithologie, 3 volumes, 1820–1822), the world’s first ornithological journal, Ornis or the newest and most important of ornithology (Ornis oder das neueste und wichtigste der Vögelkunde; 3 issues, 1824–1827) and The entire bird catch (Der gesamte Vogelfang) (Brehm 1855).

It is evident that Brehm was in contact with Johann Jakob Heckel, as a note at *Anserbrevirostris* “Heckel” in the copy of Handbook of the natural history of all birds in Germany (Brehm 1831: 844–845), which is still kept in the department, indicates. Furthermore, an entry in the acquisition list of the NHMW´s Bird collection for the year 1828 (1828.X.1–20) records the acquisition of 22 bird skins through an exchange with C.L. Brehm. In return, Brehm received an ‘old *Crocodylusniloticus*’ and a ‘skin of *Equus* zebra’, both of which were given to Heckel. This entry also shows the scientific exchange between Brehm and Heckel (Hildebrandt 1929; Kleinschmidt 1955; Gebhardt 1964).

## ﻿Materials and methods

### ﻿Extended Specimen Approach (ESA) as applied to the NHMW collections in this catalogue

The ESA, as previously applied to the NHMW collections (Bogutskaya et al. 2022; Palandačić et al. 2023) and adopted in this catalogue, includes the following information: (i) external morphological image files per specimen, including individual body parts and structures (structures of particular taxonomic importance in the groups concerned (e.g., the mouth to show the shape of the lips, the disc, the serration of the fin rays, the barbels in fish; or cuticular structures in insects); (ii) radiographs where appropriate; (iii) georeferencing of geographic localities (where possible), country, and comments clarifying the locality; (v) scanned or photographed copies of labels, acquisition and/or inventory records, original description; and (vi) comments on the nomenclatural status of the type specimen(s).

It is impossible to publish all the imagery and other prepared files, so these data have been linked to the associated physical voucher specimens via a database (Fig. 1) and/or are available from the authors on request.

Dates in the species accounts are given as they appear on the labels, catalogue cards, acquisition book and main inventory book. In some cases, it is not possible to distinguish between the date of collection, acquisition, and inventory (registration) based on existing written collection information sources. However, special searches were made for historical data on the routes and times of the collection trips under consideration, and the dates of collection and geographical location of type localities were clarified.

Recent preservation condition of type specimens was evaluated by a six-point grade: very poor – poor – bad – average – good – very good. The descriptions of the conditions are based on the definitions given in the Fish collection data base and are summarised in Table 1.

**Table 1.** The descriptions used for describing the preservation condition of specimens are based on the descriptions given in the Fish collection data base and are summarised here.

Condition	Description
Very poor	Fallen apart, completely destroyed; worse than dissected, maybe should be discarded.
Poor	Specimen not good for some systematic work, e.g., scale counts, colour or shape analysis.
Bad	Specimen not good but still suitable for some systematic work, e.g., shape analysis or radiography.
Average	Suitable for systematic work like some measurements and counts.
Good	Specimen well suitable for morphological analysis and photography or demonstration, but some damage to, e.g., fins.
Very good	Nice specimen suitable for photography or demonstration characters but not completely excellent.
Excellent	100% intact specimen; should be treated with great care.

### ﻿Abbreviations

**BL**, body length; **ESA**, Extended Specimen Approach; **NHMW**, Natural History Museum of Vienna (Naturhistorisches Museum Wien); **NMW**, a traditional abbreviation used here for the Fish collection of NHMW catalogue numbers; **TL**, total length, **SL**, standard length. Abbreviations for conservation status of species follow those used in the IUCN Red List of Categories and Criteria (IUCN 2012).

### ﻿Acquisition information

The set of Acquisition Sheets is a reliable source of original primary information that accompanied the specimens at the time of their accession to the collection. However, the earliest Acquisition Sheets (1806 to 1850s) do not constitute a true register analogous to a collection catalogue (they do not contain catalogue numbers), but rather a register to identify the materials in terms of from whom they were received: purchased (with the money paid indicated), donated, or exchanged. Identifications follow a sender or a person who filled in the acquisition list, and were sometimes later corrected or some information added. Localities are not always given, have been added later, or are not accurate, as the information would have been given on labels accompanying the specimens. However, these have often faded, been damaged or lost over time. As a result, the date of collection has often been lost or omitted, and only the date of acquisition is known. See also the Remarks section in the catalogue list.

#### ﻿A comment on the acquisition information in the Orthoptera collection relevant to this catalogue

**Figure 3.**
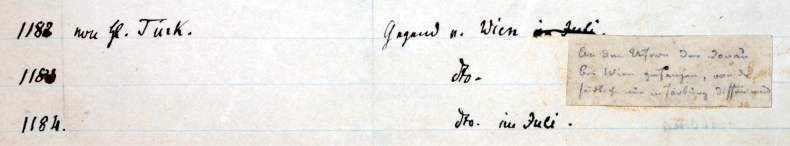
Handwritten entry 1859 of Brunner von Wattenwyl in his directory I with glued-in note.

There are no acquisition lists for the Orthoptera collection, apart from the directories (Figs 2, 3) kept by Brunner von Wattenwyl, in which he listed the acquisitions. He assigned over 26,500 numbers, with each number representing an average of 3 specimens.

#### ﻿A comment on the acquisition information in the Fish collection relevant to this catalogue

**Figure 4.**
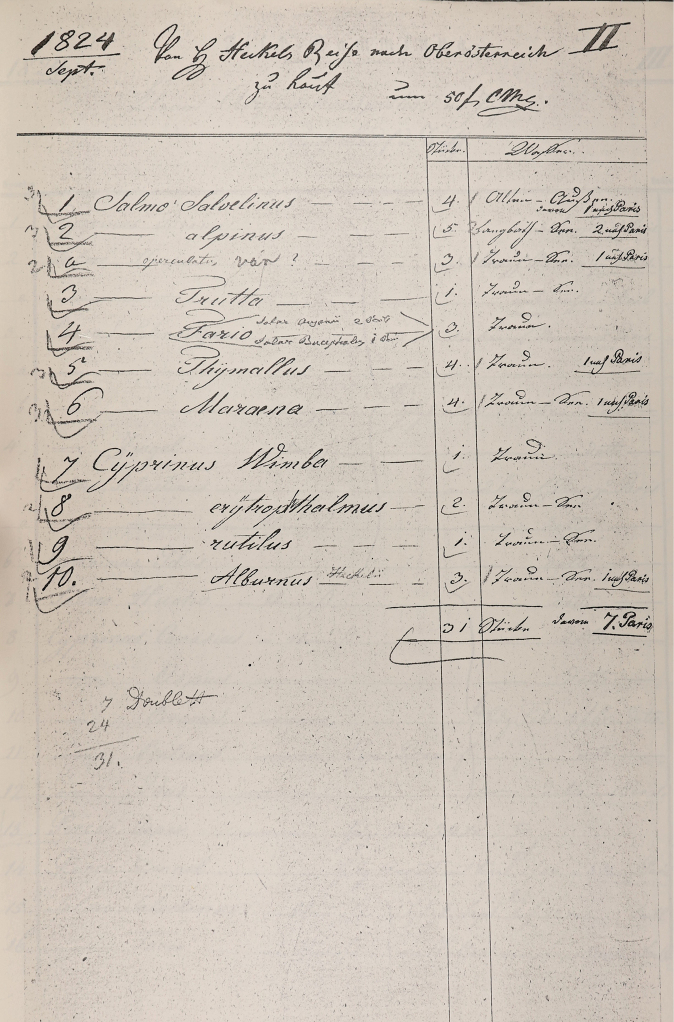
Acquisition Sheet “1824.II” for samples purchased from Heckel, collected during his trip to Upper Austria; 1824.II.10 record is for [*Cyprinus*] *mento* (here as *heckelii*; see the account on *Aspiusmento* for history of this species name).

After the acquisition of the material, the identifications follow either a sender or a person who filled in the acquisition list (Josef Natterer, Fitzinger or Heckel), with later corrections and additions by Heckel (who was the only one to study the collection taxonomically at the time). Localities were sometimes added later by Heckel (an example is shown in Fig. 4). Information was sometimes later revised by Victor Pietschmann and later curators Paul Kähsbauer, Rainer Hacker, Barbara Herzig, and Ernst Mikschi.

The source of a large number of fish specimens received (purchased) by the Fish collection in the 1825–1840s, named ‘Laboratorio’ or ‘Laboratorium’, is not entirely clear. Judging by the context, it could have been a laboratory of the Natural History Cabinet itself, which was organisationally not part of the collections and was managed separately (M. Svojtka, pers. comm.); at least this “laboratory” was involved in some kind of aquatic studies or fisheries control. It is important to note that although no localities were given in the acquisition sheets (only “purchased from Laboratory”), many (but not all) labels on (in) jars and recorded in the inventory book contain localities (mostly Vienna (“Wien”)), but also Lake Neusiedl (“Neusidlersee”) and some others; all reasonably close to Vienna. This obviously means that at least when Victor Pietschmann (curator of the Fish collection 1919–1946) started the Inventory Book (presumably, late 1940s), the old (now lost) labels existed.

### ﻿Samples

The sample set contains type series of 21 nominal species: three of parasitic worms, three of myriapods, two of insects, twelve of fishes, and one bird. Vernacular names are used here and throughout the text where generalisation is necessary, and original names when Latin names are given, for detailed classification see Table 2. They were collected within the present-day borders of the state of Vienna. However, as the type series also include specimens collected elsewhere (e.g., Lower Austria), these have also been included in the catalogue and genetic analysis. The specimens were collected between the years 1824 and 1935 and are held in the NHMW collections, preserved in alcohol or dry mounted. Details are given in Table 3.

**Table 2.** Classification of type series presented in this catalogue. The classification of fishes follows Van der Laan et al. (2023).

Coll.	Phylum	Subphylum	Class	Order	Family	Original name	Name
EV	Platyhelminthes		Trematoda	Plagiorchiida	Dicrocoeliidae	*Lyperosomumcorrigia* Braun, 1901	parasitic worm
EV	Platyhelminthes		Trematoda	Plagiorchiida	Orchipedidae	*Orchipedumtracheicola* Braun, 1901	parasitic worm
EV	Nematoda		Chromadorea	Rhabditida	Onchocercidae	*Wehrdikmansiarugosicauda* Böhm & Supperer, 1935	parasitic worm
MY	Arthropoda	Myriapoda	Diplopoda	Polydesmida	Polydesmidae	*Brachydesmussuperus* Latzel, 1884	myriapod
MY	Arthropoda	Myriapoda	Diplopoda	Julida	Julidae	*Cylindroiulusignoratus* Attems, 1927; *Iulusscandinavius* Latzel, 1884	myriapod
ORTH	Arthropoda		Insecta	Orthoptera	Rhaphidophoridae	*Locustacavicola* Kollar, 1833	insect
ORTH	Arthropoda		Insecta	Orthoptera	Tetrigidae	*Tetrixtuerki* Krauss, 1876	insect
FS	Chordata		Actinopteri	Cypriniformes	Leuciscidae	*Abramisleuckartii* Heckel, 1836; *Abramisschreibersii* Heckel, 1836; *Alburnusbreviceps* Heckel & Kner, 1858; *Aspiusmento* Heckel, 1837; *Bliccaargyroleuca* Heckel, 1843; *Cyprinusacuminatus* Heckel & Kner, 1858; *Idusmelanotus* Heckel & Kner, 1858; *Idusminiatus* Heckel & Kner, 1858; *Leuciscusvirgo* Heckel, 1852; *Phoxinusmarsilii* Heckel, 1836; *Squaliusdelineatus* Heckel, 1843; *Squaliuslepusculus* Heckel, 1852	fish
VS	Chordata		Aves	Anseriformes	Anatidae	*Anserbrevirostris* Brehm, 1831	goose

Coll. – collection; EV – Evertebrata Varia; MY - Myriapoda collection; ORTH – Orthoptera collection; FS – Fish collection (from Fischsammlung in German); VS – Bird collection (from Vogel Sammlung in German); Name – vernacular name used throughout the text when generalisation is necessary.

**Table 3.** Type series described in this catalogue.

Collection	Original name	Valid name	Inventory number	Type status	Year	Preservation
EV	*Lyperosomumcorrigia* Braun, 1901	*Lyperosomumcorrigia*	4429	SYN	1858	Ethanol
EV	*Orchipedumtracheicola* Braun, 1901	*Orchipedumtracheicola*	4472	SYN	1857	Ethanol
EV	*Wehrdikmansiarugosicauda* Böhm & Supperer, 1935	*Cercopithifilariarugosicauda*	6352	SYN	1952	Ethanol
MY	*Brachydesmussuperus* Latzel, 1884	*Brachydesmussuperus*	3661	SYN	1884	Ethanol
MY	*Cylindroiulusignoratus* Attems, 1927	*Cylindroiulusparisiorum*	8170	SYN	1884	Ethanol
MY	*Iulusscandinavius* Latzel, 1884	*Julusscandinavius*	2749	SYN	1884	Ethanol
ORTH	*Locustacavicola* Kollar, 1833	*Troglophiluscavicola*	-	SYN	1831	Dry Mounted
ORTH	*Tetrixtuerki* Krauss, 1876	*Tetrixtuerki*	-	HOLO, PARA	1859	Dry Mounted
FS	*Abramisleuckartii* Heckel, 1836	hybrid	55331, 94754	SYN	1836	Ethanol
FS	*Abramisschreibersii* Heckel, 1836	*Ballerussapa*	16584, 79462–63 74963	SYN	1825	Dry Mounted
FS	*Alburnusbreviceps* Heckel & Kner, 1858	*Alburnusalburnus*	55539	HOLO	1856	Ethanol
FS	*Aspiusmento* Heckel, 1837	*Alburnusmento*	16261, 16441, 50440, 55630, 55650, 55652, 94795	SYN	1824, 1836	Ethanol, Dry Mounted
FS	*Bliccaargyroleuca* Heckel, 1843	*Bliccabjoerkna*	16901, 54918–20, 94767	SYN	1836	Ethanol
FS	*Cyprinusacuminatus* Heckel & Kner, 1858	*Cyprinuscarpio*	52846, 52854–55, 52927–29, 52950, 53403, 94708	SYN	1836, 1840	Ethanol
FS	*Idusmelanotus* Heckel & Kner, 1858	*Leuciscusidus*	53434, 53436, 53438–39, 53455, 53467, 58775, 94805	SYN	1825, 1840	Ethanol, pharyngeal teeth
FS	*Idusminiatus* Heckel & Kner, 1858	*Leuciscusidus*	53432, 94807	SYN	1852	Ethanol, pharyngeal teeth
FS	*Leuciscusvirgo* Heckel, 1852	*Rutilusvirgo*	22373, 50626, 94733	SYN	1825, 1836	Ethanol
FS	*Phoxinusmarsilii* Heckel, 1836	*Phoxinusmarsilii*	51225, 98672	LECTO, paralecto	1825 or 1836	Ethanol
FS	*Squaliusdelineatus* Heckel, 1843	*Leucaspiusdelineatus*	49783, 50794, 94777	SYN	1840	Ethanol, pharyngeal teeth
FS	*Squaliuslepusculus* Heckel, 1852	*Leuciscusleuciscus*	49345, 49347–48, 49359, 49393	SYN	1825, 1840	Ethanol
VS	*Anserbrevirostris* Brehm, 1831	*Ansererythropus*	55170, 20928	SYN	1824, 1828	Dry Mounted

The abbreviations are official abbreviation used in the collections and also a part of the inventory numbers (e.g., NHMW-ZOO (for zoological collections)-EV4429; EV – Evertebrata Varia; MY - Myriapoda collection; ORTH – Orthoptera collection; FS- Fish collection (from Fischsammlung in German); VS – Bird collection (from Vogelsammlung in German); Year – year of collection is given, but sometimes it cannot be distinguished from the year of acquisition.

### ﻿Genetic analysis

Different tissue types were sampled depending on the animal group. For myriapods and parasitic worms, a damaged (incomplete) syntype was selected and digested for DNA extraction. For insects, a leg was carefully removed from a topotype (collected with the holotype) and digested for DNA extraction. For fish, gill rakes were taken from the right side of the body, while for dry specimens, small pieces of tissue were cut from the (historical) incision used to stuff the fish. For bird species, small pieces of toe pads were used. The insect species *L.cavicola* is represented by only one poorly preserved syntype, which is already missing a leg and was therefore considered too valuable to be further damaged for genetic analysis. See Table 3 for more information.

Laboratory procedures were carried out in accordance with all requirements for working with museum material, including the use of UV-irradiated equipment, a clean room and negative extraction controls. For alcohol preserved samples, DNA was extracted from air-dried tissue using the QIAamp® DNA Blood and Tissue Micro Kit (Qiagen) following the manufacturer’s protocol, but with the addition of 40 µl of 100 mM dithiothreitol to the lysis buffer (to enhance lysis, following Hawkins et al. 2022). For dry samples, tissue (toe pads, leg) was first pre-washed with water to remove dust and potential contaminants and then the same extraction protocol was followed. For lysis, samples were incubated overnight, but the time was extended if necessary until the tissue was completely dissolved.

After DNA extraction, the amount of double-stranded DNA was assessed by fluorometry (Qubit; ThermoFisher Scientific) using the Double-stranded DNA High Sensitivity Assay Kit. The average DNA fragment length was measured on the TapeStation system (Agilent) using High Sensitivity DNA Screen Tape. Depending on the results of these two measurements, the DNA was either sent for shotgun sequencing (IGA Technology Services, Udine, Italy). The raw sequences were then trimmed and complete mitochondrial genomes were assembled from a subset of 15 million pair-end reads using Geneious v 10.2.6 (http://www.geneious.com; for details see Palandačić et al. (2023)). Alternatively, two overlapping fragments of the cytochrome oxidase I (COI) barcode region were amplified by polymerase chain reaction (PCR) using specific primers designed in this study (see Table 4). For two parasitic worm species no COI sequences were available in GenBank to use as a basis for primer design, so 18S and 28S sequences were used instead. The proportions and conditions of the PCR reactions followed the protocol described in Antognazza et al. (2023), with an annealing temperature of 54 °C. The PCR products were then purified using the PCR Purification Kit (Qiagen) and sent to Mycrosynth (Balgach, Switzerland) for bidirectional sequencing using PCR primers.

After sequencing, smaller sequence fragments were visually inspected, aligned using MEGA 6.0 (Tamura et al. 2013) and, if multiple fragments were successfully amplified and sequenced, combined into single sequences. During this process, overlapping fragments were checked for congruence. Sequences of the same taxa and, where available, of geographical proximity, were then downloaded from GenBank. The programme MEGA 6.0 (Tamura et al. 2013) was used to construct simple neighbour-joining trees to compare the genetic information of the species with the sequences from GenBank.

**Table 4.** Primers used for polymerase chain reaction and sequencing.

Collection - Original name - Valid name - Gene	Primer Name	Sequence (5´-3´ direction)
EV - *Lyperosomumcorrigia* - *Corrigiacorrigia* - 28S	LcorriF1	TTCATCGAGCTTCCTTGCCA
	LcorriR1	GCTAACGAGCTACCTGCCAT
	LcorriF2	GTTAAACCGGCCTTGCGATG
	LcorriR2	ACAGAACCATCACGGTCAGC
EV - *Orchipedumtracheicola* - *Orchipedumtracheicola* - 18S	OtracheiiF1	CGCTGCTCGTATTCTGGTCC
	OtracheiiR1	AACCGGCAAGTGGAACTCAC
	OtracheiiF2	GTGAGTCGGTGTCGTGGTT
	OtracheiiR2	GAAGCATGCCAACCAACCG
EV - *Wehrdikmansiarugosicauda* - *Cercopithifilariarugosicauda* - COI	WrugoF1	GACCAGGAAGTAGTTGAA
	WrugoR1	CAGCCTCACTAATAATACCA
MY - *Brachydesmussuperus* - *Brachydesmussuperus* - COI	BrachySuperF1	GCACCCGATATGGCTTTTCC
	BrachySuperR1	AGACCACTAGCCAAAGGAGGA
	BrachySuperF2	GGAAATTGGGGTTGGTACTGGA
	BrachySuperR2	AGAAGAAGCCCCAGCTAAGT
MY - *Cylindroiulusignoratus* - *Cylindroiulusparisiorum* - COI	CylinIgnoF1	TCCGCTGTTGAAAAAGGTGC
	CylinIgnoR1	ATGAAGCACCCGCTAAGTGT
	CylinIgnoF2	GATATGGCCTTCCCCCGTTT
	CylinIgnoR2	ACAGAAGGACCTGAGTGTGA
MY - *Iulusscandinavius* - *Julusscandinavius* - COI	JulScandiF1	ACCCTGGGAGTTTAATTGGAGA
	JulScandiR1	AATCGAGGGAAAGCTATGTC
	JulScandiF2	AATTGATTAGTACCTTTAAT
	JulScandiR2	AGGGCCAGAGTGAGAAATGT
ORTH - *Tetrixtuerki* - *Tetrixtuerki* - COI	Ttuerki_F1	TTCATCTTCGGGGCATGAGC
	Ttuerki_R1	AATCGGAGGGTTTGGTAATTGA
	Ttuerki_F2	TAGTAGTAACAGCTCACGCATTTAT
	Ttuerki_R2	AGATATGGCATTCCCGCGAATA
FS - *Abramisleuckartii* – hybrid - COI	FishF1	TCAACCAACCACAAAGACATTGGCAC
	AleuckR1	TATTACGAAGGCGTGGGCAGT
	AleuckF2	AACGTCATCGTTACTGCCCA
	AleuckR2	ACGATGGGGGTAGAAGTCAGA
FS - *Abramisschreibersii* - *Ballerussapa* - COI	FishF1	TCAACCAACCACAAAGACATTGGCAC
	BsapaR1	AGAAAATTATTACGAAGGCGTGGG
	BsapaF2	GTCACTTTTAGGCGATGACCAAAT
	BsapaR2	TCGTGGGAATGCTATATCAGGT
FS - *Alburnusbreviceps* - *Alburnusalburnus* - COI FS - *Aspiusmento* - *Alburnusmento* - COI FS - *Bliccaargyroleuca* - *Bliccabjoerkna* - COI FS - *Leuciscusvirgo* - *Rutilusvirgo* - COI	FishF1	TCAACCAACCACAAAGACATTGGCAC
	BlicR1	CGTGGGCGGTAACGATGACA
	BlicF2	CTAAGCCAACCCGGGTCAC
	BlicR2	TCAGGCGCACCGATTATTAGT
FS - *Idusmelanotus* - *Leuciscusidus* - COI FS - *Idusminiatus* - *Leuciscusidus* - COI	FishF1	TCAACCAACCACAAAGACATTGGCAC
	LeuiduR1	TGGTCATCGCCTAAAAGTGACCC
	LeuiduF2	CCCTAAGCCTCCTTATTCGGG
	LeuiduR2	AGTCAATTTCCGAACCCGCC
FS - *Squaliusdelineatus* - *Leucaspiusdelineatus* - COI	FishF1	TCAACCAACCACAAAGACATTGGCAC
	SdeliR1	TCATCGCCTAAAAGTGACCCAGG
	SdeliF2	GGAATAGTGGGGACTGCCTT
	SdeliR2	ATCGGGCGCACCAATCATTA
FS - *Squaliuslepusculus* - *Leuciscusleucisus*- COI	FishF1	TCAACCAACCACAAAGACATTGGCAC
	Leuleu_R1	CGTGGGCGGTAACGATAACATTG
	Leuleu_F2	GCCGAACTAAGCCAACCCG
	Leuleu_R2	GCCAATCATTAGTGGGACGAG
VS - *Anserbrevirostris* - *Ansererythropus*	Aerythro F1	GCACCGCACTCAGCCTATTA
	Aerythro R1	CAGTTGCCGAATCCTCCGAT
	Aerythro F2	ACCGCTCACGCCTTTGTAATA
	Aerythro R2	TGGATGAGGCTAGTAGGAGGAG

FishF1 (Ward et al. 2005) is a general barcoding primer used for fishes.

## ﻿Results

### ﻿Extended specimen approach and samples

A total of 16 original descriptions, 17 drawings and illustrations, 64 acquisitions, registries, and labels, 48 catalogue cards, 91 radiographs, 239 image files (photographs and scans) were produced (Table 5).

**Table 5.** Digital items (image files, pdfs) prepared in the course of the project.

Category of digital item	Content	Collection					Total
		EV	MY	ORTH	FS	VS	
Original descriptions	Printed text	2	3	2	14	1	22
Drawings, illustrations	Graphic				17		17
Acquisition books, registries, labels	Handwritten text	9	5	3	38	9	64
Catalogue cards	Text		3		48	1	52
Radiographs	Digitised x-ray films				89	2	91
Image files (photos and scans)	Specimens (different aspects), specimen parts	18	25	18	174	6	241
Genetic information	Deposited at GenBank		1	1	10	1	13
**TOTAL**		29	37	24	390	20	= 500

### ﻿Genetic analysis

The results of the DNA extraction are shown in Table 6. The highest DNA concentration was measured in the goose sample (30.4 ng/µl), whereas all parasitic-worm samples seem to be devoid of DNA, or at least the DNA is below the detection limit. Based on DNA concentration and fragmentation, two fish samples were sent for shotgun sequencing: Cacu1 (NMW (FS) 52846, *Cyprinusacuminatus*) and Imini1 (NMW (FS) 53432, *Idusminiatus*). For Cacu1, the sequences obtained allowed the assembly of a complete mitochondrial genome (coverage >35) and can possibly be used for the assembly of the draft genome, whereas for Imini1 most of the sequences turned out to be contaminations.

**Table 6.** DNA concentration.

Collection	Original name	Valid name	Inv. No.	Lab ID	DNA concentration (ng/µl)	Result
EV	*Lyperosomumcorrigia*	*L.corrigia*	4429	Lcorr	too low	PCR not successful
EV	*Orchipedumtracheicola*	*Orchipedumtracheicola*	4472	Otrache	too low	PCR not successful
EV	*Wehrdikmansiarugosicauda*	*Cercopithifilariarugosicauda*	6352	Wrugo	too low	PCR not successful
MY	*Brachydesmussuperus*	*Brachydesmussuperus*	3661	Bsuper1	0,184	C1+C2 COI fragments 214 bp long with primers 167 bp long without primers GB No. PP576055
MY	*Cylindroiulusignoratus*	*Cylindroiulusparisiorum*	8170	Cigno	0,144	PCR not successful
MY	*Iulusscandinavius*	*Julusscandinavius*	2749	Jscandi	7,96	PCR not successful
ORTH	*Troglophiluscavicola*	*Troglophiluscavicola*	/	/		Only one damaged syntype, not sampled
ORTH	*Tetrixtuerki*	*Tetrixtuerki*	/	Ttuerki	0,404	C2 COI fragment 147 bp long with primers 101 bp long without primers GB No. PP579753
FS	*Abramisleuckartii*	hybrid	55331	Aleu1	0,6	PCR not successful
FS	*Abramisschreibersii*	*Ballerussapa*	79462	Abram2	0,568	C1+C2 COI fragments 266 bp long with primers 217 bp long without primers GB No. PP576053
FS	*Abramisschreibersii*	*Ballerussapa*	16584	Abram1	0,302	PCR not successful
FS	*Alburnusbreviceps*	*Alburnusalburnus*	55539	Abrevi1	0,188	C2 reverse sequence turns out to be a contamination
FS	*Aspiusmento*	*Alburnusmento*	55630	Amento1	3,34	C1 COI fragment 162 bp long with primers 114 bp long without primers GB No. PP579756
FS	*Aspiusmento*	*Alburnusmento*	55629	Amento2	3,84	PCR not successful
FS	*Aspiusmento*	*Alburnusmento*	50440	Amento3	1,68	C1 COI fragment 162 bp long with primers 114 bp long without primers GB No. PP579755
FS	*Aspiusmento*	*Alburnusmento*	55650	Amento4	1,67	PCR not successful
FS	*Aspiusmento*	*Alburnusmento*	55652	Amento5	2,56	C1 COI fragment 162 bp long with primers 114 bp long without primers GB No. PP579754
FS	*Aspiusmento*	*Alburnusmento*	16441	Amento6	2,1	PCR not successful
FS	*Aspiusmento*	*Alburnusmento*	16261	Amento7	0,134	PCR not successful
FS	*B.argyroleuca*	*B.bjoerkna*	54918	Bargy4	1,33	C2 COI fragment 152 bp long with primers 112 bp long without primers GB No. PP579757
FS	*Cyprinusacuminatus*	*Cyprinuscarpio*	52846	Cacu1	16,2	Shot-gun Reads after trimming 68 895 309 Complete mitochondrial genome Possibility of a draft genome assembly. GB No. (COI) PP576059 GB No. (complete mt) PP621518
FS	*Idusmelanotus*	*Leuciscusidus*	53434	Imel1	2,4	C1+C2 COI fragments 243 bp long with primers 193 bp long without primers GB No. PP576058
FS	*Idusmelanotus*	*Leuciscusidus*	58775	Imel2	0,26	PCR not successful
FS	*Idusmelanotus*	*Leuciscusidus*	53432	Imini1	6,69	Shot-gun: only contaminates C2 COI fragment 170 bp long with primers 149 bp long without primers GB No. PP579758
FS	*Leuciscusvirgo*	*Rutilusvirgo*	50626	Lvir1	0,9	C1+C2 COI fragments 250 bp long with primers 218 bp long without primers GB No. PP576056
FS	*Phoxinusmarsilii*	*Phoxinusmarsilii*	51225	/	/	Three previously published partial sequences of the genes: MF408203 (partial cytb) MF407956 (partial COI) MN818242 (partial ITS1)
FS	*Squaliusdelineatus*	*Leucaspiusdelineatus*	50794	Sdeli1	0,929	PCR not successful
FS	*Squaliuslepusculus*	*Leuciscusleuciscus*	49345_1	Sleb1	1,1	C1+C2 COI fragments 242 bp long with primers 192 bp long without primers GB No. PP576057
VS	*Anserbrevirostris*	*Ansererythropus*	55170	Aerythro	30,4	C1+C2 COI fragments 262 bp long with primers 220 bp long without primers GB No. PP576054

PCR – polymerase chain reaction, COI – cytochrome oxidase I, cytb – cytochrome b, ITS1– internal transcribed spacer 1; GB No. - GenBank Accession Number; mt - mitochondrion.

Two overlapping fragments of COI (designated C1 and C2) were successfully amplified and sequenced in the myriapod Bsuper1 (NMW (MY) 3661, *Brachydesmussuperus*), fish samples Abram2 (NMW (FS) 16584, *Abramisschreibersii*), Imel1 (NMW (FS) 53434, *Idusmelanotus*), Lvir1 (NMW (FS) 50626, *Leuciscusvirgo*), Sleb1 (NMW (FS) 49345, *Squaliuslepusculus*), and the bird species Aerythro1 (NMW (VS) 55170 *Anserbrevirostris*). While in the insect sample Ttuerki (no inv. number given; *Tetrixtuerki*) and the fish samples Abrevi1 (NMW (FS) 55539, *Alburnusbreviceps*), Amento1 (NMW (FS) 55630, *Aspiusmento*), Bargy4 (NMW (FS) 54918, *Bliccaargyroleuca*) and Imini1 (NMW (FS) 53432, *Idusminiatus*) either C1 or C2 was successfully amplified and sequenced. In the remaining samples, DNA extraction, amplification, and/or sequencing were not successful.

### ﻿Catalogue of nomenclatural types of taxa described based on specimens from the state of Vienna

#### ﻿Chromadorea and Trematoda type series

**Trematoda: Plagiorchiida: Dicrocoeliidae**

**1. *Lyperosomumcorrigia* Braun, 1901**

**Original publication of the name.**Braun (1901: 946).

**Figure 5.**
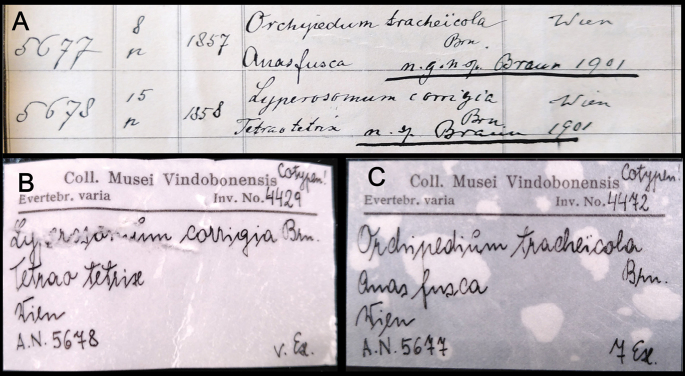
**A** old inventory records for syntypes of *Lyperosomumcorrigia* (No. 5678) and **B, C***Orchipedumtracheicola* (No. 5677), present-day labels.

**Figure 6.**
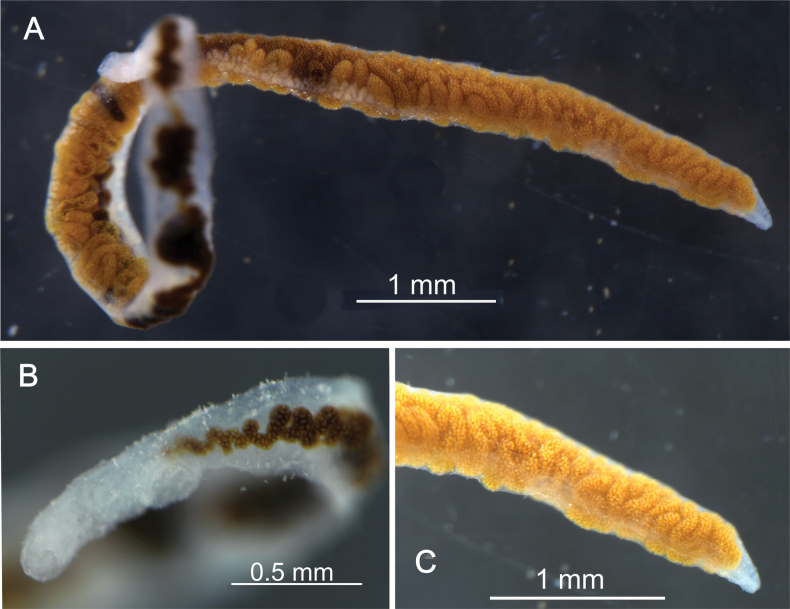
A syntype of *Lyperosomumcorrigia* (EV4429) **A** total view **B** anterior **C** posterior end of the body.

**Syntypes.**NHMW EV4429 (old inventory number 5678, 15 specimens in alcohol) (Fig. 5a, b); all 15 are currently found, of which one whole animal was used for DNA extraction. Preservation condition: average (Fig. 6).

**Remarks.** The year 1858 given in the Inventory Book (Fig. 5) is the date of acquisition rather than collecting; the host specimen was registered in the old collection (Wiener Sammlung) under the number 376 (Braun 1901: 946).

**Type locality.** Vienna; from the intestine of *Tetraotetrix* (Linnaeus, 1758) (= *Lyrurustetrix*, the black grouse).

**Distribution.** Gastrointestinal parasite of Galliformes in the Alpine area (Italy, France, Austria) (Tizzani et al. 2021).

**Etymology.** The species name is a Latin noun meaning shoelace or tie, from *corrigō* (smooth out, make straight).

**Taxonomic status.** Valid as *Corrigiacorrigia* (Braun, 1901).

**Conservation status.** Not assessed for the IUCN Red List.

**Genetic information.** Genetic analysis was not successful.

##### Trematoma: Plagiorchiida: Orchipedidae

**2. *Orchipedumtracheicola* Braun, 1901**

**Original publication of the name.**Braun (1901: 943).

**Figure 7.**
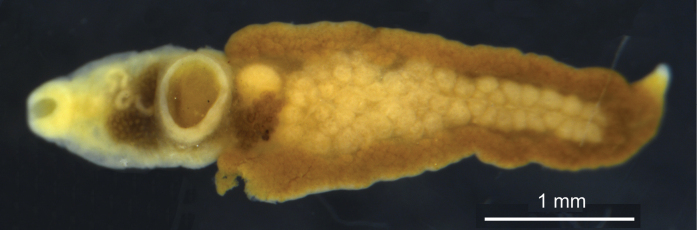
A syntype of *Orchipedumtracheicola* Braun, 1901 (EV4472).

**Syntypes.**NHMW EV4472 (old inventory number 5677, 8 specimens) (Fig. 5a, c); seven (in alcohol) are currently found, of which one whole animal used for DNA extraction. Preservation condition: good (Fig. 7).

**Type locality.** Vienna; in the trachea of *Anasfusca* Linnaeus, 1758 (the velvet scoter), collected in October 1857 (Braun 1901: 943).

**Remarks.** The host specimen was registered in the old collection (Wiener Sammlung) in 1857 under the number 377.

**Distribution.***Orchipedumtracheicola* in reported from trachea of water birds in North America and Europe (Webster 1959).

**Etymology.** The name *tracheicola* is a Latin compound noun, from *trachea* (windpipe) and *cola* (inhabitor, one who inhabits), referring to the finding of the syntypes in trachea of an avian host.

**Taxonomic status.** Valid as *Orchipedumtracheicola* Braun, 1901.

**Conservation status.** Not assessed by the IUCN.

**Genetic information.** Genetic analysis was not successful.

##### Chromadorea: Rhabditida: Onchocercidae

**3. *Wehrdikmansiarugosicauda* Böhm & Supperer, 1953**

**Original publication of the name.**Böhm and Supperer (1953: 96).

**Figure 8.**
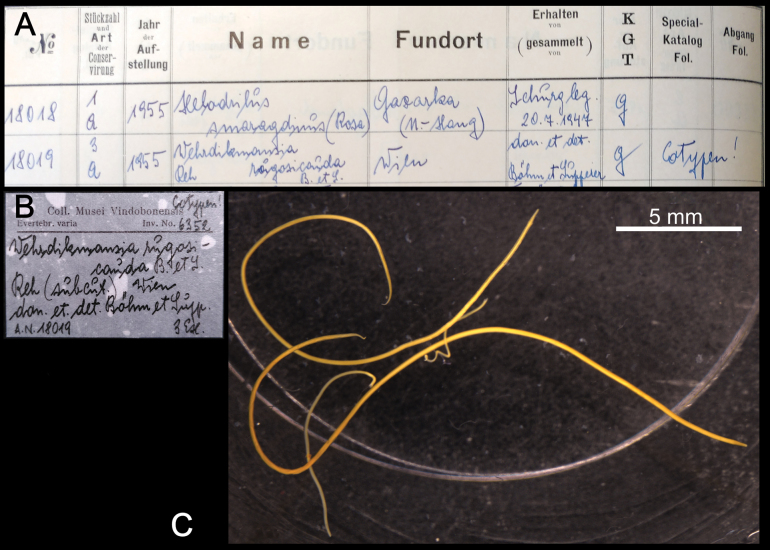
**A** old inventory record for syntypes of *Wehrdikmansiarugosicauda* (No. 18019) **B** present-day label (Nr. 6352) **B, C** total view of three extant syntypes.

**Syntypes.**NHMW EV6352 (old inventory number 18019, three specimens, donated by Böhm and Supperer in 1955; Fig. 8a, b); three (in alcohol) are currently found, of which one half was used for DNA extraction. Preservation condition: good (Fig. 8c).

**Remarks.** The species was described based on four syntypes in total: three female and one male (Böhm and Supperer 1953: 95).

**Type locality.** Vienna; from the subcutaneous connective tissue of the back in the lumbar region of *Capreoluscapreolus* (Linnaeus, 1758) (roe deer, collected in March 1952 (Böhm and Supperer 1953: 96).

**Distribution.** The species is a subcutaneous filarial nematode of roe deer *Capreoluscapreolus* (Linnaeus, 1758) in Europe (Lefoulon et al. 2014), mainly in Central Europe, Britain, Ireland, and southern Scandinavia. Also introduced into New Zealand and North America.

**Etymology.** The species name is a feminine Latin adjective, from *rugosa* (with wrinkles, folds, or creases) and cauda (tail), referring to the area rugosa, a peculiar feature in males.

**Taxonomic status.** Valid as *Cercopithifilariarugosicauda* (Böhm & Supperer, 1953).

**Conservation status.** Not assessed by the IUCN.

**Genetic information.** Genetic analysis was not successful.

#### ﻿Diplopoda type series

##### Diplopoda: Polydesmida: Polydesmidae

**1. *Brachydesmussuperus* Latzel, 1884**

**Original publication of the name.**Latzel (1884: 130).

**Figure 9.**
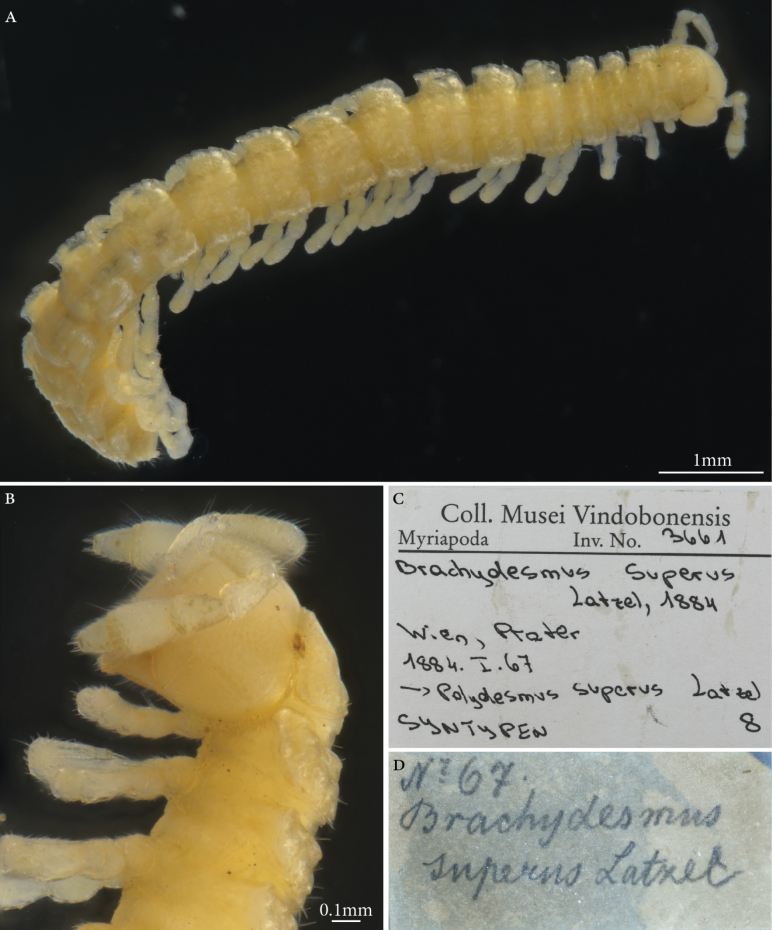
*Brachydesmussuperus* Latzel, 1884, male syntype (MY3661) **A** habitus, dorsolateral view **B** head and anteriormost bodyrings **C, D** labels.

**Syntypes.**NHMW MY3661; two males, two females, one juvenile, three fragments (in alcohol), one micro-preparation with gonopods, “Nr 67.”, Latzel leg. (Fig. 9). Preservation condition: good.

**Remarks.**Latzel (1884) mentioned he had studied more than 60 specimens, most of which are from the Prater in Vienna. Other type localities mentioned in Latzel (1884: 132) are “Mähren, Ober und Westungarn”, corresponding today to Czech Republic, Slovakia, and west Hungary. All the syntypes in NHMW are from Prater.

**Type locality.** Vienna, Prater.

**Etymology.** Not mentioned in the original description. However, the prefix *super* (above/upper) might indicate the fact that the species lives in the upper soil layers, but this remains a tentative explanation.

**Distribution.** Nearly Pan-European species. Anthropochorous and has spread beyond its natural range.

**Taxonomic status.** Valid. To date, around 21 subspecies of *Brachydesmussuperus* have been described, mostly by Verhoeff (1891, 1895, 1907, 1928, 1930a, 1930b, 1932, 1941, 1942, 1951, 1952) and Attems (1908, 1927).

**Conservation status.** Not assessed by the IUCN.

**Genetic information.** Two overlapping fragments of the mitochondrial COI region (LabID Bsuper1; 167 bp in total, GenBank Accession No. PP576055) were successfully amplified. Nucleotide blast search with a subsequent alignment of the sequences and simple neighbour-joining tree analysis showed the closest relative to be *B.superus*, GenBank Accession No. HQ966183, from Lombardy, Italy. In this case, sequencing of the type has irreversibly connected this COI fragment with the species name *B.superus*, which will be helpful in the subsequent taxonomic and barcoding projects.

##### Diplopoda: Julida: Julidae

**2. *Cylindroiulusignoratus* Attems, 1927**

**Original publication of the name.**Attems (1927: 199).

**Figure 10.**
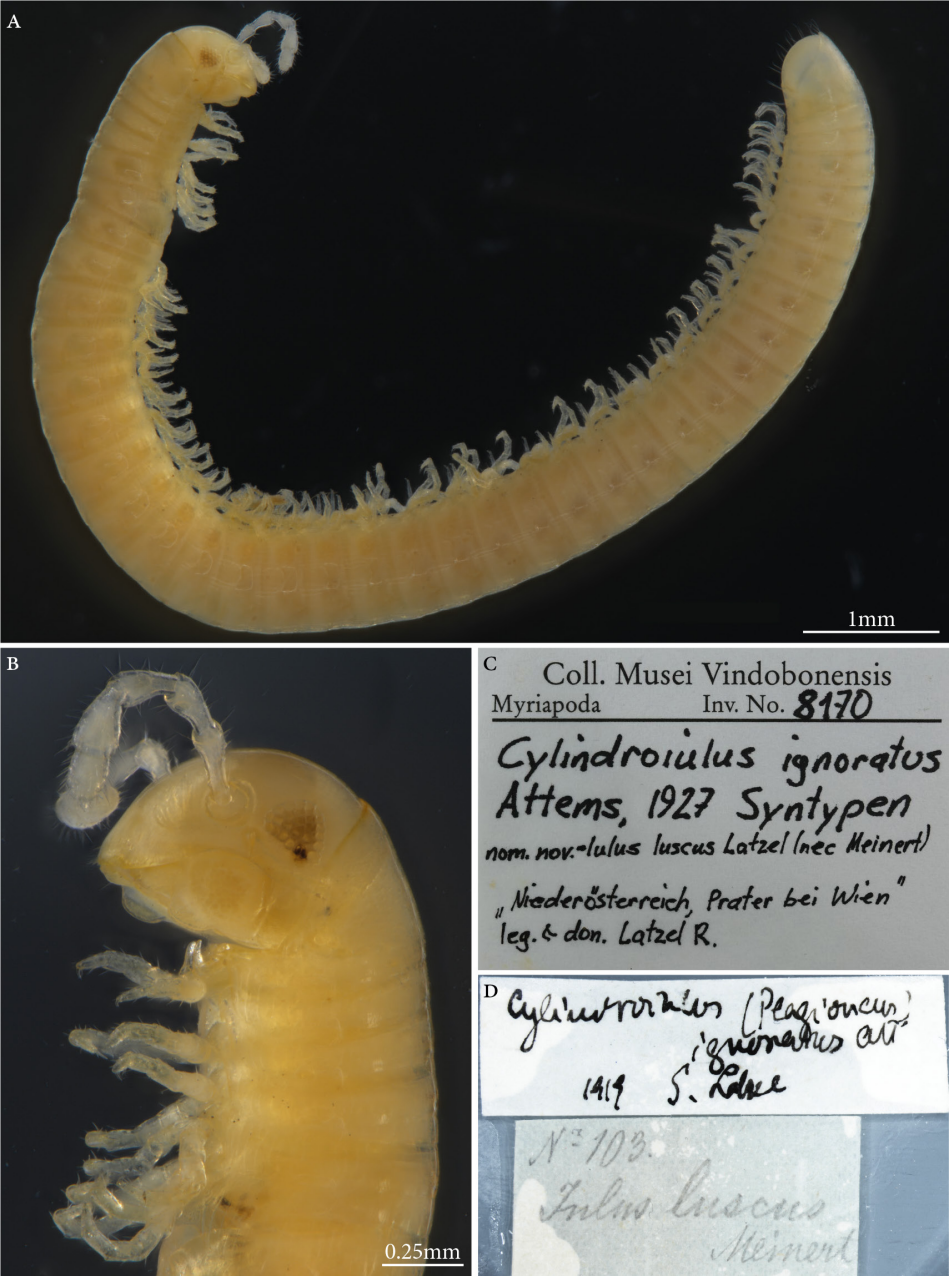
*Cylindroiulusignoratus* Attems, 1927, male syntype (MY8170) **A** habitus lateral view **B** head and anteriormost body rings, lateral view **C, D** labels.

**Syntypes.**NHMW MY8170; three males, three females, three subadults (in alcohol), “Nr 103 *Julusluscus* Meinert”, Niederösterreich, Prater bei Wien, Latzel don. leg. (Fig. 10). NHMWMY 8171; several specimens Styria, Graz, Leechwald, Rhabarberbeet, Attems leg. NHMWMY 8172; 15 specimens, one micro-preparation, Lower Austria, Laxenburg. Preservation condition: good.

**Type locality.** Vienna, Lower Austria, Styria.

**Distribution.** Mainly Central Europe, Britain, Ireland, and southern Scandinavia. Also introduced into New Zealand and North America.

**Taxonomic status.** Not valid. A junior subjective synonym of *Cylindroiulusparisiorum* (Brölemann & Verhoeff, in Brölemann 1896).

**Conservation status.** Not assessed by the IUCN.

**Genetic information.** Genetic analysis was not successful.

**3. *Iulusscandinavius* Latzel, 1884**

**Original publication of the name.**Latzel (1884: 322).

**Figure 11.**
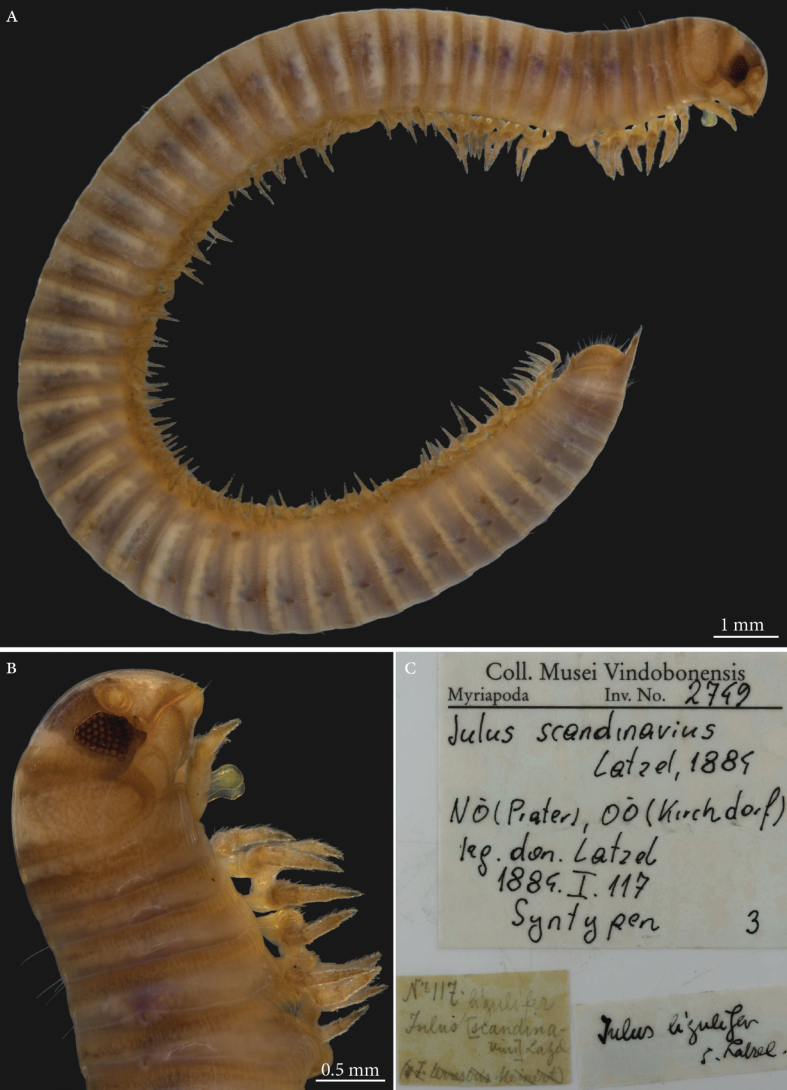
*Iulusscandinavius* Latzel, 1884, male syntype (MY2749) **A** habitus, lateral view **B** head and anteriormost body rings, lateral view **C** labels.

**Syntypes.**NHMW MY2749; two males, one female (in alcohol) (Fig. 11). Preservation condition: good.

**Remarks.** As many of the types of Robert Latzel, the original type locality of this species was not provided with precision and mentioned by Latzel (1884) as the Austro-Hungarian Empire, the crown lands of Lower Austria, Upper Austria, Bohemia, Moravia and Western Hungary. Five specimens are listed in the the acquisition book in 1884.I.117, whereas only three exist in the collection. The whereabouts of the remaining syntypes is unknown. An additional label “*Julusligulifer*” is also contained in the jar. This label must have been added subsequently as *Julusligulifer* Latzel, in Verhoeff, 1891 is a junior subjective synonym of *Julusscandinavius*.

**Type locality.** Lower Austria; Vienna, Prater, Upper Austria, Kirchdorf.

**Etymology.** Not mentioned in the original description but the name refers to the fact the author believed the species is rare in Central Europe and should most probably come from Scandinavia and Denmark (Latzel 1884: 324). The name is used as an adjective.

**Distribution.** A very common species in Central Europe with a wide distribution range. Mostly encountered in woodlands although also recorded on heaths, wetlands, humid open grassland, and sand dunes (Kime and Enghoff 2017).

**Taxonomic status.** Valid.

**Conservation status.** Not assessed by the IUCN.

**Genetic information.** Genetic analysis was not successful.

#### ﻿Insecta type series

##### Insecta: Orthoptera: Rhaphidophoridae

**1. *Locustacavicola* Kollar, 1833**

**Original publication of the name.**Kollar (1833a: 80).

**Figure 12.**
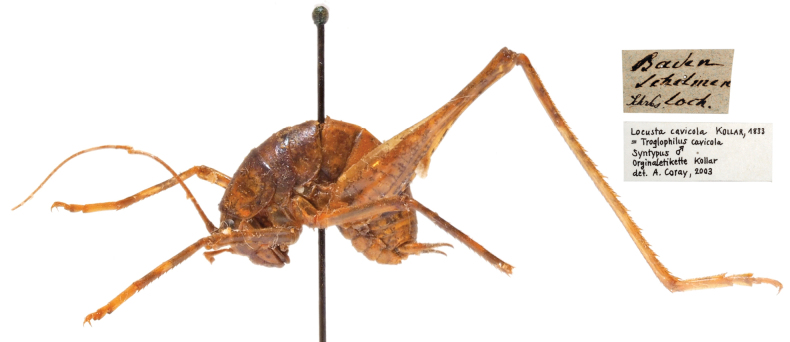
*Troglophiluscavicola*, Syntype, male, lateral view and labels.

**Syntype.** One male (dry mounted; Fig. 12). Preservation condition: poor.

**Remarks.** The original description is based on several male individuals, found by Carl von Schreibers (1775–1852), director of the United Natural History Cabinet, in the cave “Schelmenloch” south of Vienna around 1831 (Kollar 1833a; Christian 2008). Handwritten labels write Kollar det. A. Corey, 2003.

**Type locality.** Schelmenloch (cave), Baden, south of Vienna, Lower Austria.

**Etymology.** the species name is a noun, coming from the Latin word *cavum*, meaning cave dweller. The current combination *Troglophiluscavicola* by Krauss (1878) [1879] is a tautological combination of the Greek word *troglophil*, meaning cave-loving, therefore “the cave-loving cave dweller” (Christian 2008).

**Taxonomic status.** Valid as*Troglophiluscavicola* (Kollar, 1833).

**Distribution.** The main distribution area of *Troglophiluscavicola* is in southeastern Europe. From central Greece, the range extends across the Balkan Peninsula to the Bergamo Alps, the south of Graubünden to Austria. The northern limit of distribution is south of Vienna (Moog 1982; Christian 2008).

**Conservation.***Troglophiluscavicola* is in the LC category (Europe and Austria) (Hochkirch et al. 2016; Zuna-Kratky et al. 2017).

**Genetic information.** As there is only one, already damaged, syntype left, no genetic analysis was performed.

##### Insecta: Orthoptera: Tetrigidae

**2. *Tetrixtuerki* Krauss, 1876**

**Original publication of the name.**Krauss (1876: 103)

**Figure 13.**
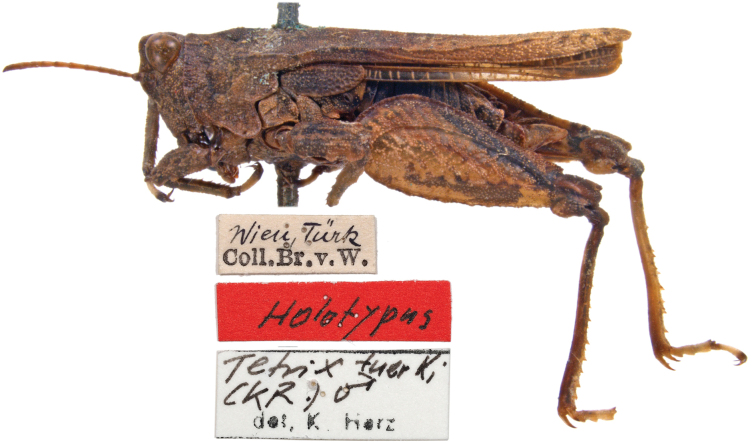
*Tetrixtuerki*, Holotype, male, lateral view and labels.

**Holotype.** One male (dry mounted; Fig. 13). Preservation condition: good.

**Remarks.** In Brunner von Wattenwyl’s directory I (Fig. 2), the specimens in question are listed with the number 1183 from the year 1859. On a glued-in note next to it is written in handwriting: “An den Ufern der Donau bei Wien gefangen, von d. südlichen nur in Färbung differierend” (“Caught on the banks of the Danube near Vienna, differing from the southern one(s) only in coloration”) (Fig. 3). This note was presumably written by Türk and handed over to Brunner von Wattenwyl together with the specimens. As southern species he probably means *T.depressa* and *T.meridionalis*, which were available to him as comparative material from the Mediterranean region. As Krauss (1876) notes, Türk described *T.tuerki* (Krauss 1876) as *T.depressa* Brisout de Barneville, 1848 (Türk 1860) and *Paratettixmeridionalis* (Rambur, 1838) as *T.meridionalis* (Türk 1862).

**Type locality.** Vienna, on flat, sandy banks of the Danube, washed by water, sparsely vegetated, in the Prater, Brigittenau, near Klosterneuburg and in several other places (Krauss 1876: 104).

**Etymology.** The species name is a patronym, a noun in the genitive, named after Rudolf Türk.

**Taxonomic status.** Valid.

**Distribution.***Tetrixtuerki* is a Pontomediterranean faunal element that is native to the Alps and mountain ranges of eastern and southern Europe, but also occurs east of the Black Sea region (Zuna-Kratky et al. 2017).

**Conservation.***Tetrixtuerki* is in the VU category for Europe (Hochkirch et al. 2016) and in the EN category for Austria (Zuna-Kratky et al. 2017).

**Figure 14.**
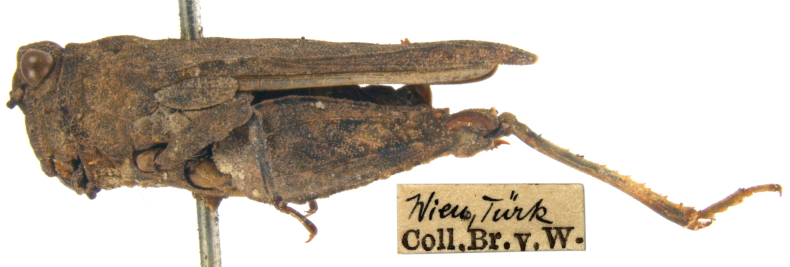
*Tetrixtuerki*, Topotype, female, lateral view and labels.

**Genetic information.** To refrain from damaging the types, a topotype specimen (Fig. 14, female) collected together with the holotype was used for genetic analysis. Genetic analysis was successful for one fragment of the COI region (LabID Ttuerki; 101 bp in total, GenBank Accession No. PP579753). This short sequence is identical to the unpublished COI sequences from the GenBank (GU706152–GU706154) collected at the Austrian-Germany border (47.50 N; 11.50 E).

#### ﻿Actinopteri type series

##### Actinopteri: Cypriniformes: Leuciscidae*

classification according to van der Laan et al. (2023).

**1. *Abramisleuckartii* Heckel, 1836**

**Original publication of the name.**Heckel (1836: 229, pl. XX, fig. 5).

**Remarks.** This paper (Heckel 1836) was published in the Annals of the Vienna Museum of Natural History (Annalen des Wiener Museums der Naturgeschichte), which was a short living journal; only two volumes have been published, the first one, as commonly cited, in 1836 (for 1835) and the second one, in parts, in 1837–1840 (Ahnelt and Mikschi 2008). However, the volume (or some part of it) was possibly published in 1835; there is also a separate dated 1835.

**Figure 15.**
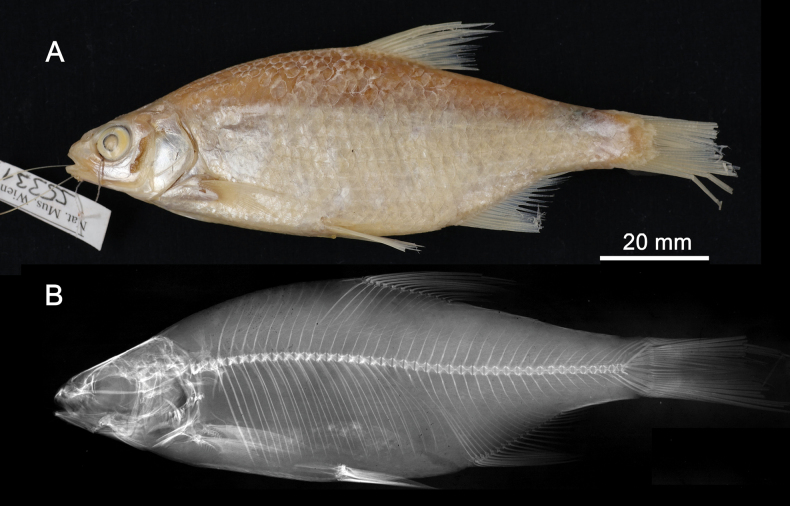
*Abramisleuckartii* syntype, NMW 55331, SL 105 mm **A** left lateral view **B** radiograph.

**Syntypes.**NMW 55331 (a specimen in alcohol), 94754 (a pair of pharyngeal bones with teeth). Recent measurements: TL ca 130 mm (the caudal fin damaged), SL 105 mm (Fig. 15). Preservation condition: good.

**Figure 16.**
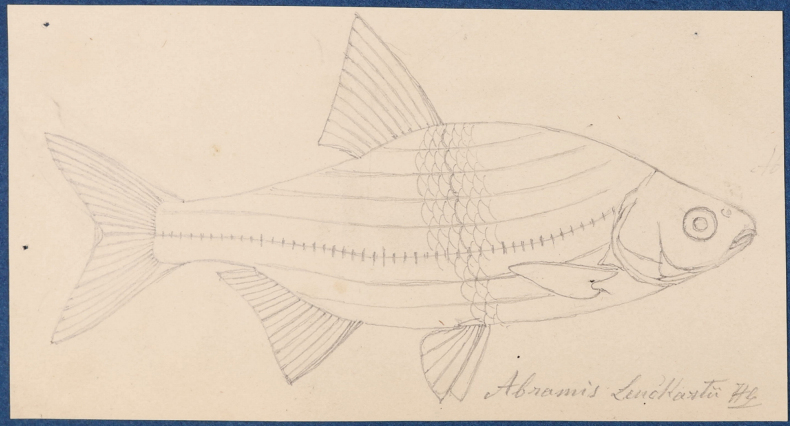
A draft by Heckel of *Abramisleuckartii* for Heckel (1836: pl. XX, fig. 5) (NHMW Archive).

**Remarks.** The original description is based on more than one individual (the numbers of countable feature are given as ranges, e.g., the number of branched anal-fin rays is 15–17). The extant syntype (NMW 55331) has 15 (if two last rays are counted as one, as it was accepted at the time), similar to a syntype in the original drawing (Fig. 16). Acquisition record 1836.I.10 indicates three specimens.

**Type locality.** “Schnellfliessenden Stellen der Donau bei Fischment unter Wien” (“Fast-flowing parts of the Danube near Fischment downstream of Vienna”) in the original description (Heckel 1836: 230); acquisition 1836.I.10 reads only “Danube”.

**Etymology.** The species name is a patronym, a noun in the genitive; named for Friedrich Sigismund Leuckart, a German naturalist (1794–1843).

**Taxonomic status.** Hybrid between *Rutilusrutilus* (Linnaeus, 1758) × *Abramisbrama* (Linnaeus, 1758) (Günther 1868: 214). This opinion is correct as the specimen has character states intermediate between *Rutilus* and *Abramis* (especially, the shape of the posterior process of the basioccipital and the number of branched anal-fin rays). The availability of the name is not affected if it is applied to a taxon later found to be of hybrid origin, Art. 17 of the Code (International Commission on Zoological Nomenclature 1999).

**Distribution.** Only known by the syntypes.

**Conservation status.** None (a hybrid).

**Genetic information.** Genetic analysis was not successful.

**2. *Abramisschreibersii* Heckel, 1836**

**Original publication of the name.**Heckel (1836: 227, pl. XX, fig. 4)

**Figure 17.**
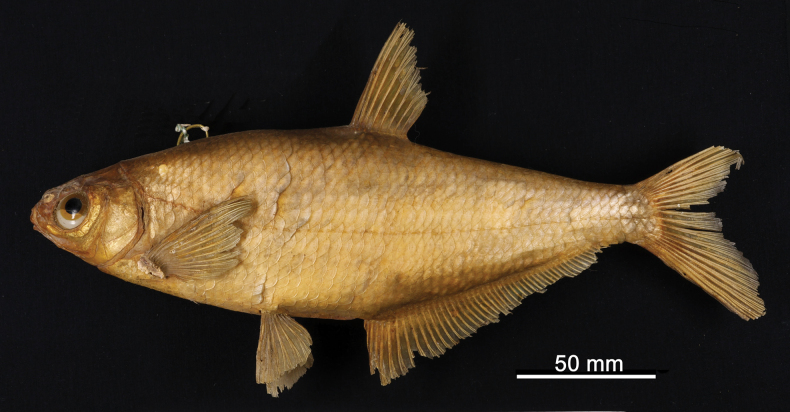
*Abramisschreibersii* syntype, NMW 16584, SL ca 215 mm, left lateral view.

**Syntypes.**NMW 16584 (1), 79462–63 (1, 1); all are stuffed individuals. Recent measurements (TL, SL): NMW 16584 ca 255 mm, ca 215 mm (Fig. 17); 79462 ca 223 mm, ca 185 mm; 79463 ca 230 mm, ca 195 mm. Preservation condition: average.

**Remarks.** The original description is based on more than one individual (the numbers of countable feature are given as ranges, e.g., the number of branched anal-fin rays is 39–43); 38 branched anal-fin rays in the illustrated individual. The extant syntypes are all with Acquisition Number 1825.V.32 which indicates three specimens.

**Type locality.** “Schnellfliessenden Stellen der Donau unter Wien, auch in der March kommt er vor“ (“Fast-flowing parts of the Danube below Vienna, also in the March“) (Heckel, 1836: 228). Acquisition 1825.V.32 (as *Balerus* (sic) neu species): “ II. Semester 1825, vom Laboratorio zukauft”.

**Etymology.** The species name is a patronym, a noun in the genitive; named for Carl Franz Anton Ritter von Schreibers (1775–1852), an Austrian naturalist and botanist, the director of the Natural History Cabinet since 1806.

**Taxonomic status.** Treated as a synonym of *Abramissapa* (Pallas, 1814) since as early as Heckel and Kner (1857: 115), now in the genus *Ballerus*.

**Distribution.***Ballerussapa* is native in large rivers draining to Black, Azov, Caspian, and Aral seas. Introduced elsewhere (Northern Dvina, Volkhov, Rhine, Vistula) (Freyhof and Kottelat 2008a).

**Conservation.** IUCN: *Ballerussapa* is in the LC category (Freyhof and Kottelat 2008a). In the Red Data List of Lower Austria (Wolfram and Mikschi 2007: 110) as Not Endangered (“nicht gefährdet”).

**Genetic information.** DNA extraction was performed on scales from two stuffed specimens, NMW 16584 and 79462, but genetic analysis was successful only on the latter. Two overlapping fragments of the mitochondrial COI regions (LabID Abram2; 217 bp in total, GenBank Accession No. PP576053) were successfully amplified in the specimen NMW 79462. The sequence was identical to the *Ballerussapa* sequences from Austria (Zangl et al. 2022).

**3. *Alburnusbreviceps* Heckel & Kner, 1857**

**Original publication of the name.**Heckel and Kner (1857: 134, fig. 69).

Although dated 1858, the book (Heckel and Kner 1857) was already printed in December 1857 as shown by Svojtka et al. (2012: 60). Rudolf Kner donated it to the library of the Zoological-Botanical Society at the meeting on 2 December 1857 (Anonymous 1857b: 158).

**Holotype.**NMW 55539 (in alcohol). Recent measurements: TL 152 mm, SL 124 mm. Preservation condition: average.

**Figure 18.**
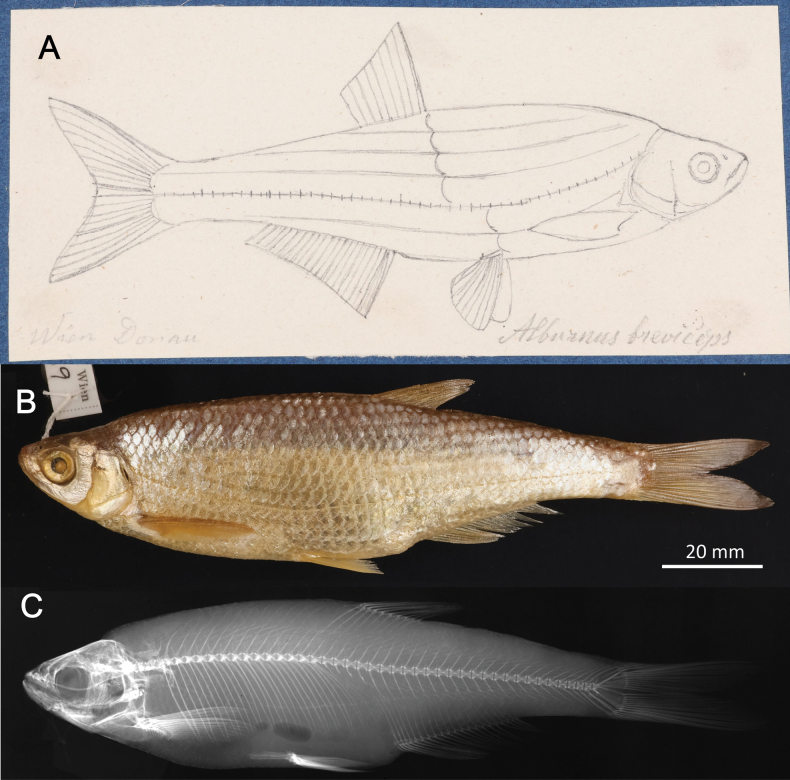
*Alburnusbreviceps* holotype, NMW 55339, SL 124 mm **A** a draft drawing by Heckel of the described specimen from the Danube at Vienna, for Heckel and Kner (1857: fig. 69) (NHMW Archive) **B** left lateral view **C** radiograph.

**Remarks.** The original description is based on one individual of 5 Zoll (Viennese inches) of total length (= 131.7 mm) (Fig. 18a). Recent measurements (TL, SL): 132 mm, 114 mm. The length, number of branched anal-fin rays (19) and the lateral-line scales (50) suit to those in the 55539 specimen (Fig. 18b, c). Acquisition number, 1856.VII.63, indicates one specimen (as “*Alburnusbreviceps* Heckel”).

**Type locality.** Not provided in the original description (Heckel and Kner 1857: 134–135). Acquisition entry 1856.VII.63: Danube, Vienna. Acquisition 1856.VII contains a remark that it had been earlier recorded as 1856.I. (this number is still indicated in respective labels and cards).

**Etymology.** The species name is an adjective, short-headed, comes from the Latin word *brevis*, meaning short, and *ceps*, head.

**Taxonomic status.** Synonymised with *Alburnusalburnus* (Linnaeus, 1758) soon after the description (e.g., Günther 1868: 313).

**Distribution.***Alburnusalburnus* is native in most of Europe north of Caucasus, Pyrénées, and Alps, eastward to Ural and Emba. Locally introduced elsewhere (Spain, Italy, the Irtysh River) (Freyhof and Kottelat 2008b).

**Conservation**. IUCN: *Alburnusalburnus* is in the LC category (Freyhof and Kottelat 2008b). In the Red Data List of Lower Austria (Wolfram and Mikschi 2007: 111) as Not Endangered (“nicht gefährdet”).

**Genetic information.** Genetic analysis was not successful.

**4. *Aspiusmento* Heckel, 1836**

**Original publication of the name.**Heckel (1836: 225, pl. XIX, fig. 3).

**Remarks.** The name of the species in the acquisition records (listed below) (e.g., Fig. 4) are given as *heckelii* because Fitzinger (1832: 335) had already published this name (unavailable as neither indication (reference) nor description were provided) as “AspiusHeckelii. Mihi. Im Gebirge; in Flussen. Bisher nur in Ober-Osterreich gefunden; in der Traun. Sehr selten“ (In the mountains; in rivers. So far only found in Upper Austria; in Traun. Very rare).

On the other hand, Heckel knew that Agassiz was going to describe the species as the two ichthyologists were well acquainted. At the time Agassiz stayed in Vienna in 1830, he was preparing a multi-volume monography titled ‘Histoire naturelle des poissons d’eau douce de l’Europe centrale, ou description anatomique et historique des poissons qui habitent les lacs et les fleuves de la chaine des Alpes et les rivières qu’ils reçoivent dans leur cours’ (‘Natural history of the freshwater fishes of Central Europe, or anatomical and historical description of the fishes which inhabit the lakes and rivers of the Alps and the rivers which they receive in their course’). This work, which remained unfinished, has a curious history. Agassiz undertook it in 1828, in Munich, having the plates of his future work drawn by Joseph Dinkel. On August 30, 1830, Agassiz published a prospectus in German and French announcing the book: “In the arrangement of the materials I followed the procedure that I am going to indicate: everything is arranged by natural families, each of which is the subject of a particular monograph. General considerations on the class of fishes should first serve as an introduction to my work, but what I have to say cannot be appreciated until after the publication of all the particular facts, I have had to return these generalities at the end of the work. Each monograph therefore begins with the indication of the general external characteristics, and the main organizational features of a detailed exposition of the characters of each genus, I have given the anatomy as complete and as concise as possible of the species…” (Surdez 1973: 69–70). However, only three volumes, on Salmonidae, were published while the volumes on cyprinids have never appeared. It can be assumed that Heckel decided to publish the new species by himself but with attribution to Aggassiz, as *Aspiusmento* Ag. (Heckel 1836: 226): “Später erhielt das hiesige Museum durch die Güte des Herrn Professor Agassiz sehr schöne Exemplare seines AspiusMento aus München; ich habe nun diese Exemplare auf das sorgfältigste mit jenen aus der Traun verglichen, …” (“Later, the local museum received by the generosity of Professor Agassiz very beautiful specimens of his *AspiusMento* from Munich; I have now most carefully compared these specimens with those from the Traun...”).

**Syntypes.**NMW 16261 (1) and 16441 (1), both stuffed; 50440 (1), 55630 (1), 55650 (2) and 55652 (1), in alcohol; NMW 94795 (a pair of pharyngeal bones).

Recent measurements (SL): NMW 16261, 140 mm; NMW 16441, 134 mm, NMW 50440, 221 mm; NMW 55630, 190 mm; NMW 55650, 157 and 137.5 mm, NMW 55652, 219 mm. Preservation condition: poor to good.

**Remarks.** The original description (Heckel 1836: 225–226) clearly indicates the three samples of specimens on which it was based. All three samples are still present among specimens labelled as syntypes.

**Figure 19.**
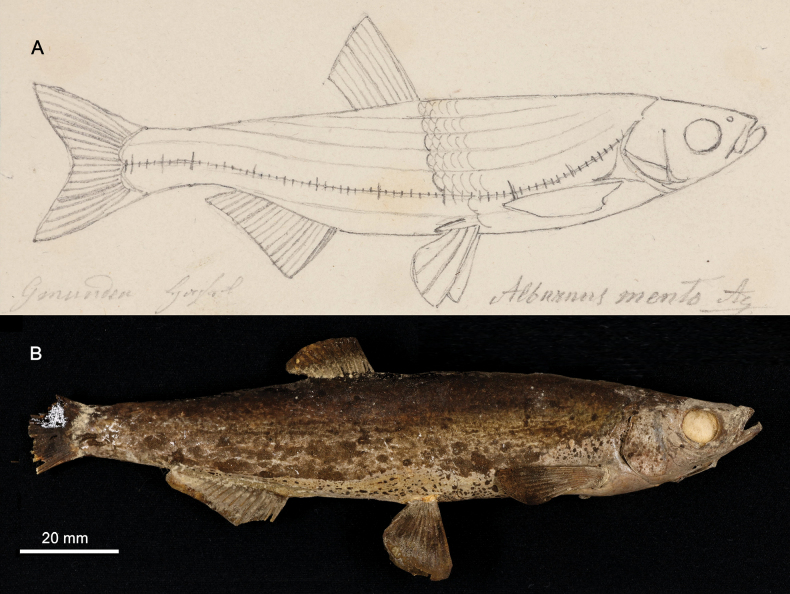
*Aspiusmento***A** a draft drawing by Heckel of a syntype from Gmunden, for Heckel (1836: pl. XIX, fig. 3) (NHMW Archive) **B** syntype NMW 16441, SL 140 mm, right lateral view.

**Figure 20.**
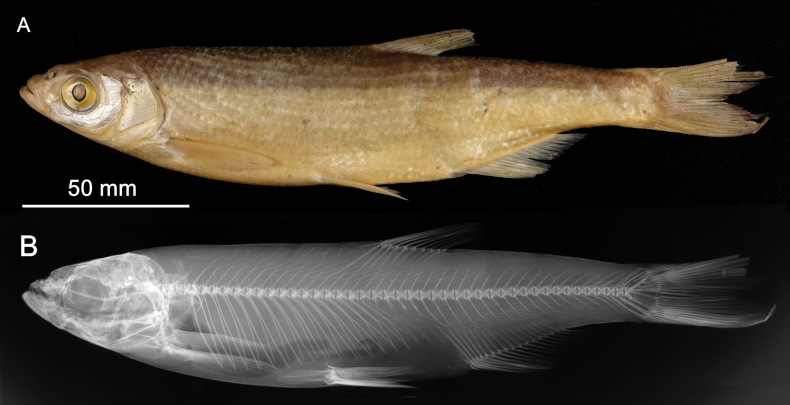
*Aspiusmento*, syntype, NMW 55630, SL 188 mm, Danube, Vienna **A** left lateral view **B** radiograph.

1. NMW 16261 and 16441: specimens collected by Heckel during his travel to Upper Austria in September 1824 in Lake Traun at Gmunden (the acquisition number 1824.II.10); two specimens are still in NMW (16261 and 16441) (Fig. 19), and one was sent to Muséum national d’Histoire naturelle in Paris (MNHN-IC-0000-3894). NMW 94795, a pair of pharyngeal bones (locality: Gmunden; labelled (handwritten by Heckel) as *AspiusHeckelii*) apparently belongs to one of the two stuffed specimens. The two NMW specimens have a standard length (139.8 mm and 134 mm, respectively) which corresponds to total length equalling “Spanne” [Handspanne] (the distance between the end of the little finger and the end of the thumb that is ca 18–22 cm), mentioned in the original description.
2. NMW 50440, 55650 and 55652: specimens received later [than 1824] from Agassiz. These specimens are most probably those registered under the acquisition number 1830.II.3. The acquisition 1830.II contains seven entries in total (e.g., 1830.II.1 is for *Gobiouranoscopus*) and reads “Bavaria. November 1829. Von Herrn Leopold Fitzinger durch Kauf”. This acquisition is made by Jos. Natterer and 1830.II.3 refers to later by Heckel, 6 individuals (4 were sent to Lüttich (Liege) on exchange). The labels for NMW 50440, 55650, and 55652 (with the acquisition number 1830.II.3) reading ”Durch Agassiz aus München” (by Agassiz from Munich) appeared later, at Steindachner’s time, and are most probably based on information from the Heckel’s description of *Aspiusmento* Agassiz as a synonym of *Aspiusheckelii* Fitzinger (Heckel 1836: 225).
3. NMW 55630: one specimen, 9 Zoll (Viennese inches) long (total length; 237 mm) from the Danube near Vienna. This specimen (Fig. 20) was registered under the acquisition number 1836.I.19: Danube at Vienna. November 1835.

**Type locality.** The original description reads (Heckel 1836: 225–226): 1. “…bei Gmunden in Ober-Oesterreich in September 1824, und zwar ziemlich häufig unter der über die Traun führenden Brucke” (near Gmunden in Upper Austria in September 1824, quite often under the bridge over the Traun) (the acquisition number 1824.II.10: Traun, … Heckels Reise durch Oberösterreich… Nr. 80); 2. Bavaria. November 1829 (acquisition number 1830.II.3, purchased from Leopold Fitzinger; 3. Danube at Vienna. November 1835 (acquisition number 1836.I.19).

**Etymology.** The species name is a noun in apposition; an Italian *mento* for chin, mentum, reflecting a peculiar feature of the fish, its protruding chin.

**Taxonomic status.** After recent revisions of the genus *Alburnus*, it is commonly considered that *Alburnusmento* is a valid species (e.g., Bogutskaya and Naseka 2004: 79; Freyhof and Kottelat 2007: 214, 217; Kottelat and Freyhof 2007: 171; Bogutskaya et al. 2017: 106; Freyhof et al. 2018: 130). Lectotypification may be required as the syntypes include both the lacustrine form, the “true *Alburnusmento*” in its modern concept (the syntypes from Traunsee, Austria, and from Bavaria), and a riverine fish (NMW 55630, from Vienna) that belongs to a recently described species, *Alburnussava* Bogutskaya, Zupančič, Jelić, Diripasko & Naseka, 2017 (Bogutskaya et al. 2017).

**Distribution.***Alburnusmento* is a lacustrine species in most subalpine lakes in Germany and Austria.

**Conservation.** IUCN: *Alburnusmento* is in the LC category (Freyhof and Kottelat 2008c). In the Red Data List of Lower Austria (Wolfram and Mikschi 2007: 21, as *Chalcalburnuschalcoidesmento*) not referred to any of threatened categories.

**Genetic information.** Amplification and sequencing of only the first of the two overlapping fragments of the mitochondrial COI region (LabIDs Amento1, Amento3, Amento5; 114 bp in total, GenBank Accession Nos. PP579754–PP579756) was successful in two lacustrine (NMW 50440 and 55652) and one riverine specimen (NMW 55630). In this short fragment, all three sequences of all three specimens differ in one nucleotide base. Nucleotide blast search puts them in the same group as *A.mento* and other “shemayas” from Turkey (e.g., GenBank Accession Nos. MT407383, NC019574, MG182572, MT407410, MW649504). In the publication reporting on Austrian DNA barcode inventory of fish species (Zangl et al. 2022), *A.mento* was not mentioned. Thus, further research is needed to resolve the taxonomic status of this group and of the type specimens.

**5. *Bliccaargyroleuca* Heckel, 1843**

**Original publication of the name.**Heckel (1843: 1007, pl. 1).

**Remarks.**Heckel (1843) is a part of the Vol. 1, Part 2, of Russegger‘s Reisen in Europa, Asien und Afrika mit besonderer Rücksicht auf die naturwissenschaftlichen Verhältnisse der betreffenden Länder, unternommen in den Jahren 1835 bis 1841. Also published as a special print under the title Abbildungen und Beschreibungen der Fische Syriens nebst einer neuen Classification und Charakteristik sämmtlicher Gattungen der Cyprinen (Illustrations and descriptions of the fish of Syria along with a new classification and characteristics of all genera of cyprinids). It contains pp. 1001–1012 Zahn-System der Cyprinen (Tooth system of the cyprinids), pp. 1013–1043 Dispositio systematica familiae Cyprinorum (Systematic arrangement of the family Cyprinidae), and pp. 1044–1099 Süsswasser-Fische Syriens (Freshwater fishes of Syria). A volume of figures for this publication was published later, presumably in 1843–1838, in Stuttgart.

In the original publication, Heckel only refers to the structure of the pharyngeal teeth of a single specimen (Heckel 1843: pl. 1), and the description is unambiguously available as providing a clear diagnosis referring to a single species name. Though, in later times, the date and authorship of the species name was often thought to be Heckel and Kner (1857: 120), presumably following Günther (1868: 306) and Berg (1916: 305).

It is not quite clear why Heckel did not refer in his publications to the Linnaeus’ (1758) name of the species, *Cyprinusbjoerkna*, and is always only citing the species name as *blicca*, e.g., *Cyprinusblicca* of Bloch (1782) and Fries and Eckstrom (Fries et al. 1837: tab.12) (Heckel 1843: 1032; Heckel and Kner 1857: 120). As Heckel (1843: 1032) established a new genus, *Blicca*, with pharyngeal teeth 2.5–5.2 (in contrast to 3.5–5.3 in *Abramis*), it seems quite probable that the new name *argyroleuca* was given just to avoid Striktland’s tautonymy (to avoid the *Bliccablicca* combination) as it was a common practice at the time (also, see *Idusmelanotus* below).

**Figure 21.**
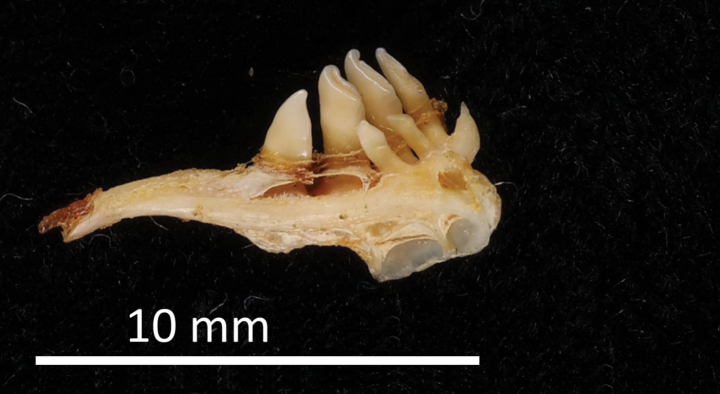
Possible holotype (or a syntype) of *Bliccaargyroleuca*, NMW 94767, left pharyngeal bone.

**Holotype or a syntype**. NMW 94767, left pharyngeal bone (uppermost tooth in the longer row broken) (Fig. 21).

**Figure 22.**
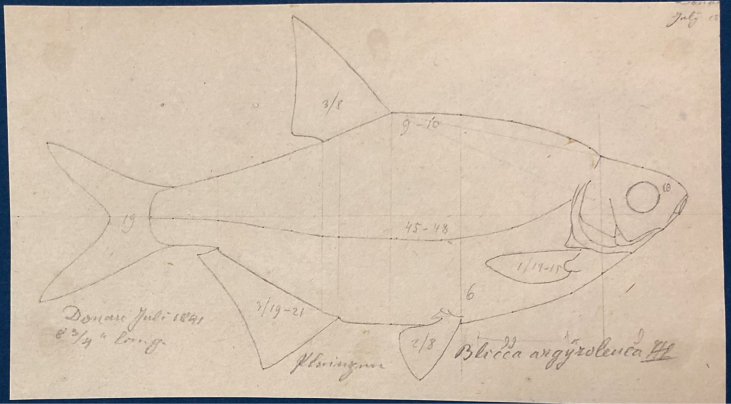
A draft by Heckel of the drawing of a specimen (or a possible syntype) of *Bliccaargyroleuca*, representing a fish collected in the Danube in July 1841; this may be a composite (note ranges of counts) (NMH Archive).

**Remarks.** A single (left) pharyngeal bone is now kept in the collection. As mentioned in Introduction, in many cases, individuals from which the pharyngeal bones were taken for a special study, are still kept in NHMW. We failed to find any individual lacking pharyngeal bones that could be a source of the original description. Although it is worth mentioning, that Heckel had apparently examined more than one whole individual identified by him as *Bliccaargyroleuca* before he published the description as his original drawing represents the fish collected in the Danube in July 1841 (Fig. 22) with counts given as ranges, e.g., 19–21 anal-fin branched rays. At present, there are no specimens of *Blicca* in the Fish collection that could be confirmed as collected in July 1841 from the Danube. However, we cannot exclude that pharyngeal teeth morphology was studied in more than one specimen.

Apparently due to the misinterpretation of the date and authorship, all specimens in NMW lots, historically (since Heckel’s time) labelled as *Bliccaargyroleuca*, became considered as syntypes of the species: NMW 16901 (2; 1840, Fish market in Berlin), 54918 (6; 1836, Vienna), 54919 (4; 1836, Neusiedlersee), 54920 (1; 1842, Pommern). All 13 of them have the pharyngeal bones intact.

**Figure 23.**
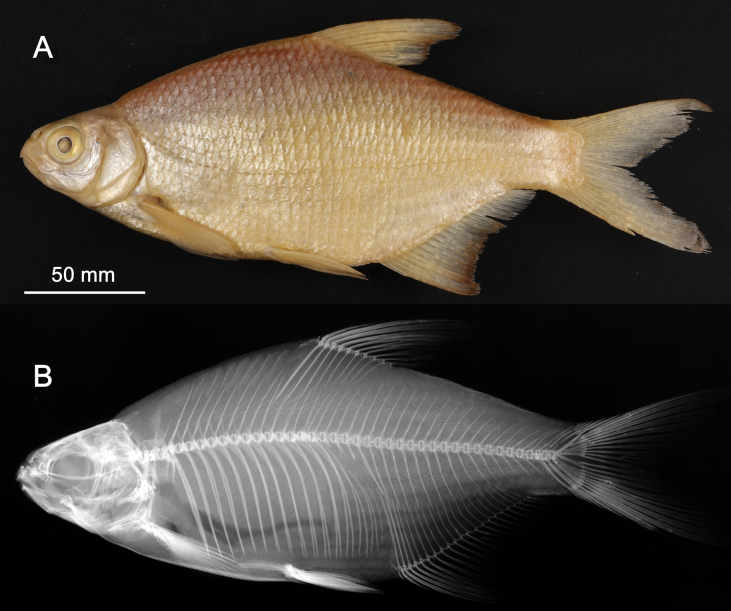
*Bliccaargyroleuca*NMW 54918:2, SL 156 mm, Danube at Vienna **A** left lateral view **B** radiograph, with intact pharyngeal bones.

Among the mentioned above possible syntypes, NMW 54918 (6 specimens, SL 111–222 mm) (Fig. 23) is the only lot with individuals collected in the Danube at Vienna (acquisition 1836.I.9).

**Type locality.** Not provided in the original description (Heckel 1843: 1007). The label of the possible holotype NMW 94767 reads Oder that is later included in the range of the species by Heckel and Kner (1857: 122).

**Etymology.** The species name is a patronym, a noun in the genitive; named for Friedrich Sigismund Leuckart, a German naturalist (1794–1843).

**Taxonomic status.** Synonym of *Bliccabjoerkna* (Linnaeus, 1758).

**Distribution.***Bliccabjoerkna* is native to North, Baltic, White, Black (south to Rioni drainage) and Caspian Sea basins, Atlantic basin southward to Adour drainage and Mediterranean basin in France (Hérault and Rhône drainages), in Aral, Marmara and Anatolian Black Sea basins west of Ankara. Locally introduced elsewhere (Spain, northeastern Italy, France) (Freyhof and Kottelat 2008d).

**Conservation status.** IUCN: *Bliccabjoerkna* is in the LC category (Freyhof and Kottelat 2008d). In the Red Data List of Lower Austria (Wolfram and Mikschi 2007: 113) as Not Endangered (“nicht gefährdet”).

**Genetic information.** Amplification and sequencing of only the second of the two overlapping fragments of the mitochondrial COI region (LabID Bargy4; 112 bp in total, GenBank Accession No. PP579757) was successful in the specimen NMW 54918. This short fragment is identical to sequences of *Bliccabjoerkna* collected in Austria (Zangl et al. 2022).

**6. *Cyprinusacuminatus* Heckel & Kner, 1857**

**Original publication of the name.**Heckel and Kner (1857: 57, fig. 22).

**Remarks.** The name is objectively invalid being a junior homonym of *Cyprinusacuminatus* Richardson, 1846.

**Figure 24.**
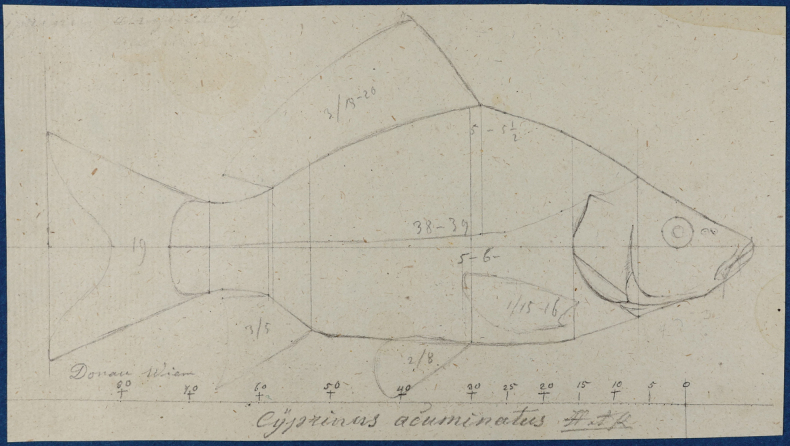
A draft by Heckel of his drawing of *Cyprinusacuminatus* for Heckel and Kner (1857: fig. 22), Danube, Vienna (NHMW Archive).

The original description is based on more than one individual (the numbers of countable feature are given as ranges, e.g., the number of branched dorsal-fin rays is 18–20). Besides, Heckel refers to two of his earlier species (unavailable, nomina nuda): *Cyprinusangulatus* and *Cyprinusthermalis* “Heck. nov. spec. (Hungaria)” (Heckel 1843: 1013). Fig. 24 represents a draft (made by Heckel) of the original drawing used in the original publication (Heckel and Kner 1857: fig. 22) of an individual from the Danube at Vienna.

**Syntypes.**NMW 52846 (2), acquisition 1836.I.2, Vienna; 52854 (1) and 52855 (1), acquisition 1836.I.22, Neusiedlersee, coll. Lestrin; 52927 (1), 52928 (1), 52929 (1), 53403 (2), acquisition 1840.III.3, Plattensee (Balaton), received from “Laboratorium”; 52950 (9), acquisition 1840.III.4, Kesythely (Keszthely, Balaton), received from “Laboratorium”; 94708 (a pair of pharyngeal bones; before 1857, Heckel).

**Figure 25.**
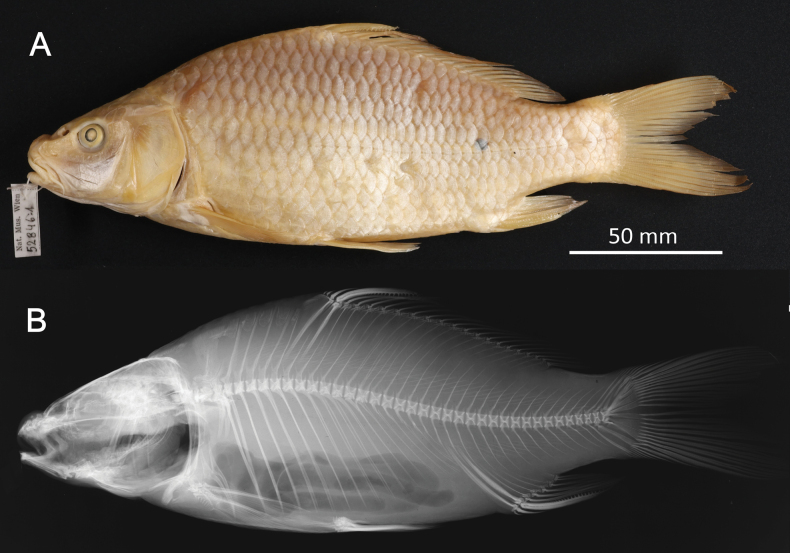
A syntype of *Cyprinusacuminatus*, NMW 52846:1, SL 182.5 mm, Danube, Vienna **A** left lateral view **B** radiograph.

Recent measurements of the Viennese syntypes, NMW 52846 (TL, SL): 230 mm, 182.5 mm (Fig. 25) and 123.5 mm, 97 mm. Preservation condition good.

**Type locality.** Danube, Neusiedler Lake and Plattensee (Balaton Lake) in the original description (Heckel and Kner 1857: 60); these localities refer to the localities of the syntypes.

**Etymology.** The species name is a Latin adjective, past participle of *acuminare* “to sharpen”, from *acumen* “a point”, and refers to the shape of the snout.

**Taxonomic status.** The name has been considered a synonym of *Cyprinuscarpio* Linnaeus, 1758, or its variety, from as early as at least Günther (1868: 26).

**Distribution.** Wild European *Cyprinuscarpio* is native to Black, Caspian and Aral Sea basins. Introduced throughout the world. Cultivated in large quantities for human food and stocked for sport fishing (Balon 1995).

**Conservation status.** IUCN: wild native European *Cyprinuscarpio* is in the category VU (under criteria A2ce) (Freyhof and Kottelat 2008e). Important to emphasize, that common carp (*Cyprinuscarpio*) is the world’s oldest domesticated and the most important aquaculture species, but the native populations are slowly but continuously declining due to diverse reasons, first of all, competition with domesticated introduced common carp. In Western Europe, there is even a debate if native common carp still exist. Also, hybridisation with domesticated introduced stocks, East Asian congeners, and their hybrids, is a serious long-term threat for the species. However, superficially pure carp (currently, it is impossible to identify pure carp by genetic analysis) are still abundant in the lower parts of rivers within its native range. Most likely, only very few stocks remain “genetically unpolluted” as a result of this long-lasting process. The average age of the spawners is estimated to be between 20–25 years, as they are a long-lived species (up to 50 years). Although no population data exists, it is suspected that in the past 60 to 75 years within the species native range, river regulation (due to channelization and dams), which impacts the species as they need flooded areas at very specific times to successfully spawn, and hybridisation with introduced stock, has caused a population decline of over 30% (Freyhof and Kottelat 2008e).

In the Red Data List of Lower Austria (Wolfram and Mikschi 2007: 38) the wild native carp is in the category 2, Critically Endangered (“stark gefährdet”).

**Genetic information.** Shot-gun sequencing resulted in over 68 million pair-end reads. Based on a subset of 15 million reads, a complete mitochondrial genome was assembled (LabID Cacu1; GenBank Accession No. for COI PP576059; for complete mitochondrial genome PP621518). According to a nucleotide blast search, the sequence with the highest identity score is OL693871, *C.carpio* from Eugene, Portland, USA. Further analysis is beyond the scope of this paper, and will be presented elsewhere.

**7. *Idusmelanotus* Heckel, 1843**

**Original publication of the name.**Heckel (1843: 1008, pl. I).

**Remarks.** In the original publication, Heckel only refers to the structure of the pharyngeal teeth, and the description is unambiguously available as providing a clear diagnosis referring to a single species name. Though, in later times, the date and authorship of the species name was often thought to be Heckel (1852a: 56, 66) (Günther 1868: 230) or Heckel and Kner (1857: 147, figs 77, 78), apparently following, e.g., Berg (1912: 163).

As Heckel (1843: 1037) established a new genus *Idus* in the same publication, it seems quite probable that the new name *melanotus* was given to just avoid Strickland’s tautonymy (to avoid the *Idusidus* combination) as it was a common practice at the time (similar to *Bliccaargyroleuca* above).

**Figure 26.**
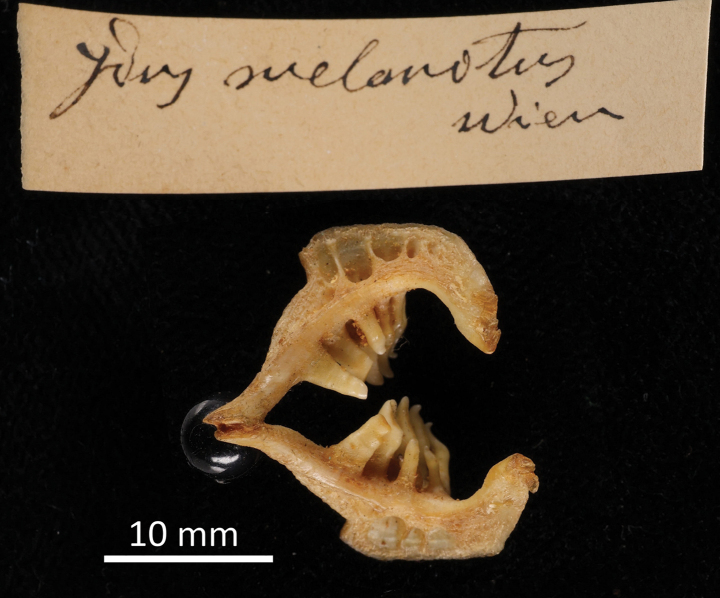
Syntype of *Idusmelanotus*, NMW 94805, a pair of pharyngeal bones, which apparently belongs to NMW 58775:1 (Fig. 27 below, see the text for explanation).

**Figure 27.**
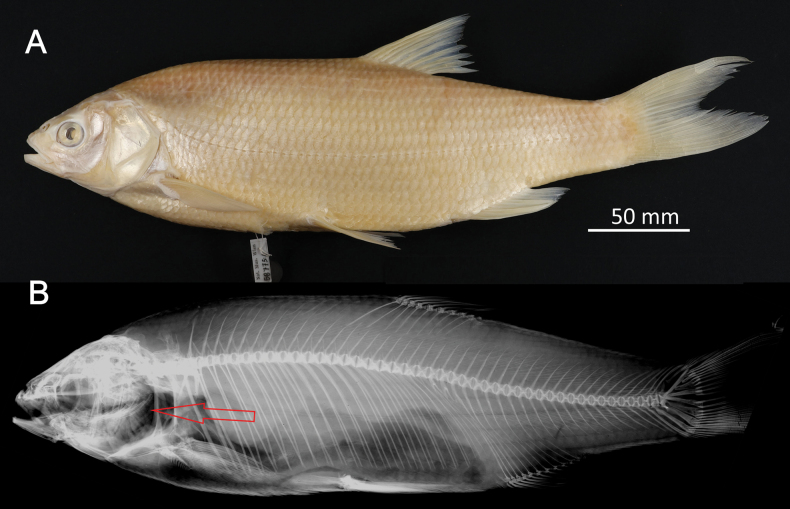
Syntype of *Idusmelanotus*NMW 58775:1 (with its pharyngeal bones separated as NMW 94805, Fig. 22) **A** left lateral view **B** radiograph; arrow indicates the lack of the pharyngeal bones.

**Figure 28.**
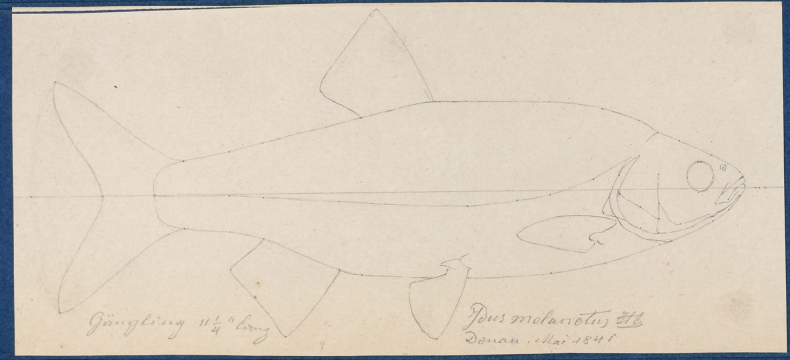
A draft by Heckel of the drawing of a specimen (a possible syntype) *Idusmelanotus*, representing a fish collected in the Danube in May 1841 (NHMW Archive).

**Syntypes.** 1. NMW 94805, a pair of pharyngeal bones (Fig. 26) labelled *Idusmelanotus* Heckel, that may belong to NMW 58775:1 (Fig. 27). 2. Specimens collected or received in the Fish collection before 1843 and lacking pharyngeal bones that may indicate that Heckel examined the teeth and used these data in the original description, as follows: “Alte Sammlung” (Vienna): 53434 (1); Acquisition 1840.VII.10–11 (Berlin, leg. Rammelsberg): 53436 (1), 53438 (1), 53467 (1); Acquisition 1825.V.35a (Bayern, leg. Langthaler): 53439 (1); Acquisition 1842.I.13 (Pommern, coll. Hornschuh): 53455 (1); Acquisition 1825.V.34 (Vienna): 58775:1. 3. The specimen in a draft of the drawing, collected in the Danube in May 1841, 11 ¼ inches long (296 mm) (Fig. 28) (absent at present from the collection).

Recent measurements of NMW 58775:1 (TL, SL): 380 mm, 291 mm. Preservation condition: good.

**Remarks.** As explained in Introduction, in many cases, cypriniform specimens from which the pharyngeal bones were taken for a special study by Heckel, were still kept in the collection. We assumed that the pharyngeal bones NMW 94805 belong to the individual under the number NMW 58775 as they suit each other by size, and NMW 58775:1 is the only one extant individual collected at Vienna before 1843, which lacks pharyngeal bones, among the whole set of extant Heckel’s *I.melanotus* specimens.

**Type locality.** Not provided in the original description (Heckel 1843: 1008). The NMW 94805 and 58775 (possibly representing one and the same individual) are from Vienna. The Danube by Vienna is also included in the range of distribution of the species by Heckel and Kner (1857: 148).

**Etymology.** The species name is a Latinized Greek adjective, *melano*, meaning black and *melanotus*, meaning the black-coloured one, alluding to the predominantly black dorsal colouration of the fish.

**Taxonomic status.** Synonym of *Leuciscusidus* (Linnaeus, 1758) since soon after the description (e.g., Günther 1868: 230).

**Distribution.***Leuciscusidus* is native to Baltic, Black, northern Caspian and North Sea basins, Atlantic basin southward to Seine and lower Loire drainages (France). Introduced to Great Britain and northern Italy (Freyhof and Kottelat 2008f).

**Conservation status.** IUCN: *Leuciscusidus* is in the LC category (Freyhof and Kottelat 2008f). In the Red Data List of Lower Austria (Wolfram and Mikschi 2007: 113) in the category 3, Endangered (“gefährdet”).

**Genetic information.** DNA extraction was performed on two specimens, NMW 53434 and 58775, but genetic analysis was successful only on the first. Two overlapping fragments of the mitochondrial COI region (LabID Imel1; 217 bp in total, GenBank Accession No. PP576058) were successfully amplified. The sequence is identical to the *L.leuciscus* or *L.idus* (which, based on COI sequences, exhibit no differences) sequences from Austria (Zangl et al. 2022).

**8. *Idusminiatus* Heckel & Kner, 1857**

**Original publication of the name.**Heckel and Kner (1857: 151, no figure).

**Remarks.** In an earlier publication, Heckel (1843: 1038) introduced the name *Idusminiatus* but did not provide any reference, figure or description leaving the name nomen nudum. Similar to the case of *Idusmelanotus*, described above, a pair of pharyngeal bones is kept labelled as “*Idusminiatus* Heckel. Hofgarten” among the Heckel’s collection of cyprinid pharyngeal bones, NMW 94807. The name became available in Heckel and Kner (1857) as above, and, as an exception, no figure of the fish is provided.

**Figure 29.**
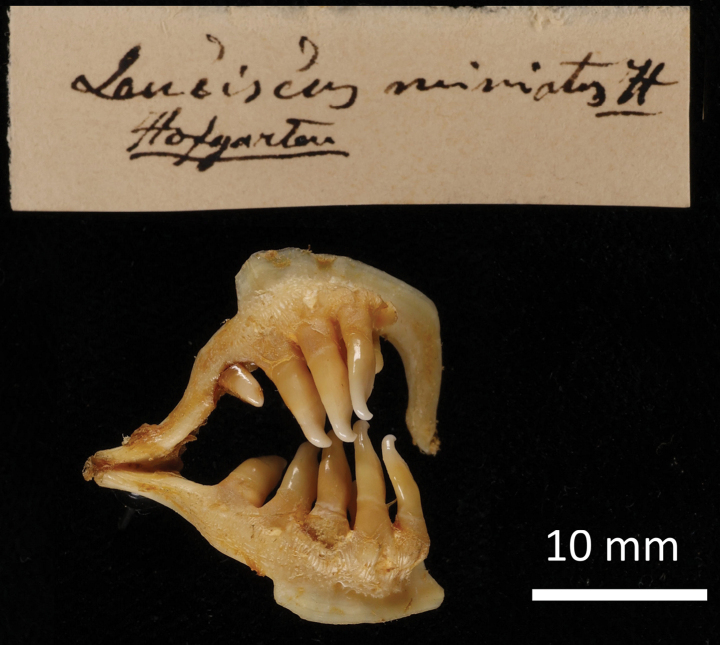
*Idusminiatus* syntype, NMW 94807, a pair of pharyngeal bones, apparently dissected out of NMW 53432 (now decomposed).

**Holotype.**NMW 53432 and a pair of pharyngeal bones, NMW 94807 (Fig. 29), that apparently belongs to this individual. Preservation condition: very poor (decomposed).

**Remarks.** The original description per se is based on a single specimen; and only one specimen was registered as *Idusminiatus* Heckel from “Hofgarten” – acquisition entry 1852.XV.1, Royal Gardens of Burg (k.k. Hofgarten), received from Court gardener (Hofgärtner) Antoine, is handwritten by Heckel. However, the text in Heckel and Kner (1857: 151–152) mentions observations on size of the species: “Reaching the size and weight of the Orfe, our longest specimens do not measure a full foot”.

**Type locality.** The species name is applied to captive fish; they had been kept in the pond of the Imperial court garden of the castle in Vienna but originated from Tyrol (Heckel and Kner 1857: 151): “For many years, numerous specimens of a fish very close to the orfe have lived in the pond of the imperial court garden of the castle in Vienna, which was supposedly first brought here from Tyrol, but which maintains and reproduces constantly in its own characteristics. Although it is therefore only a cultured fish and is limited to a single locality, we believe that we should not ignore it and distinguish it as *Idusminiatus*, a new species”. So, according to Art. 76.1.1 of the Code (International Commission on Zoological Nomenclature 1999), the type locality is Tyrol.

**Etymology.** The species name is a Latin first/second-declension adjective meaning scarlet, cinnabar-red in reference to the reddish (“blasser rot”) colouration of the back of the fish.

**Taxonomic status.** Synonym of *Leuciscusidus* (Linnaeus, 1758).

**Distribution.** As *Leuciscusidus* (above).

**Conservation status.** As *Leuciscusidus* (above).

**Genetic information.** Amplification and sequencing of only the second of the two overlapping fragments of the mitochondrial COI region (LabID Imini1; 149 bp in total, GenBank Accession No. PP579758) was successful in the specimen NMW 53432. The sequence is identical to the *L.leuciscus* or *L.idus* (which based on COI sequences exhibit no differences) sequences from Austria (Zangl et al. 2022).

**9. *Leuciscusvirgo* Heckel, 1852**

**Original publication of the name.**Heckel (1852a: 69, pl. VI, figs 1–8, mature male with breeding tuberculation, and pl. VII, figs 1–5).

**Remarks.** This paper is published in the Proceedings of the Academy of Sciences in Vienna, Mathematics and Natural Sciences class (Sitzungsberichte der Akademie der Wissenschaften in Wien, Mathematisch-Naturwissenschaftliche Klasse), Vol. 9 (1) with pagination 49–123, and numbers of plates of figures VI–XIII, and, also, as a separate with different pagination, 127–201, and numbers of plates of figures, XI–XVIII. It is one of six papers by Heckel (1850, 1851a, 1851b, 1851c, 1852a, 1852b) in Sitzungsberichte as a series of reports on his travel to the Alps area.

The original description is based on a number of individuals, the length of the described specimens is 6–15 Zoll (Viennese inches) (= 158–395 mm) (Heckel 1852a: 76); one of them was apparently dissected as the numbers of vertebrae is given, and at least four specimens are presented by the pharyngeal bones.

**Figure 30.**
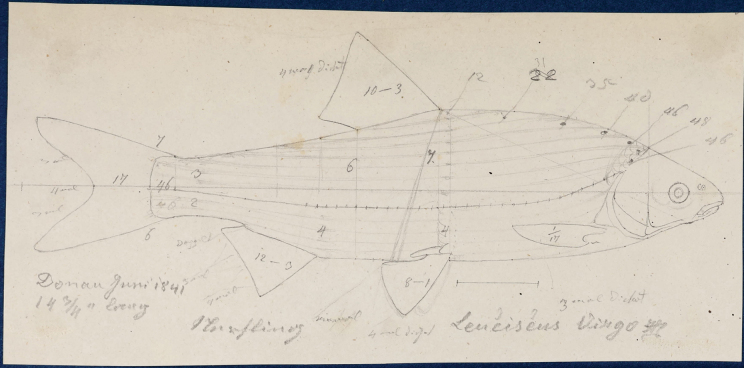
A draft by Heckel of the drawing of *Leuciscusvirgo* published in Heckel (1852a: pl. VI and VII), Danube, June 1841 (NHMW Archive).

**Syntypes.**NMW 22373 (1) and NMW 50626 (1), whole individuals in alcohol, pharyngeal bones intact; NMW 94733 (4 pairs of pharyngeal bones from fish of a variety of size). It is not clear to which acquisition numbers these individuals refer to; there are at least three acquisition entries referring to this species: 1. 1825.IV.4 (one specimen), 2. 1825.IV.4 (one specimen), both purchased in the first semester of 1825 from “Laboratorium” (supposedly, Danube at Vienna); 3. 1836.I.12 (two specimens), Danube, no other data; all three acquisition records were made by Heckel, first as *Leuciscus Jeses* but then the species name corrected (in pencil) to *virgo*. In 1825.IV.4 entry, there is a later note by Heckel in pencil “[sent] to Munich”. One more syntype, apparently not preserved as a whole fish or lost, is the one in the figures (Heckel 1852a: pl. VI–VII), collected in the Danube in June 1841, 14 ¾ Zoll (Viennese inches) long (389 mm) (Fig. 30).

**Figure 31.**
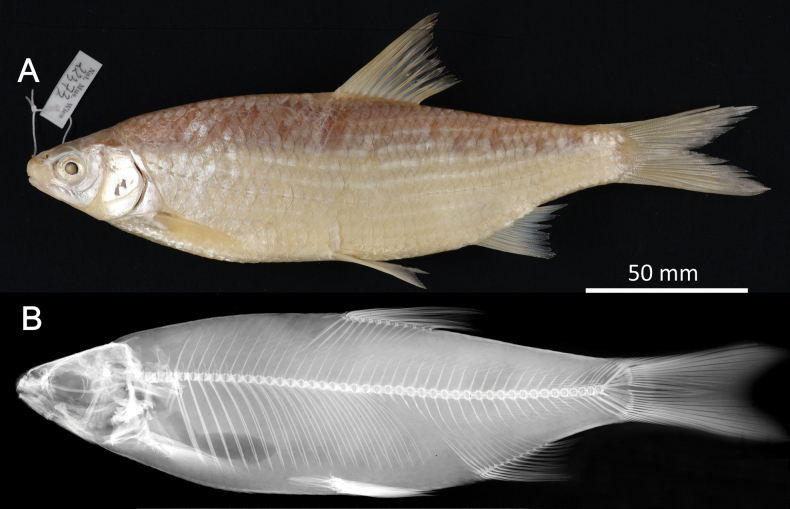
A syntype of *Leuciscusvirgo*, NMW 22373, SL 180 mm, possibly Vienna **A** left lateral view **B** radiograph.

Recent measurements of the extant syntypes collected at Vienna (TL, SL): NMW 22373 (Fig. 31), 225 mm, 180 mm; 50626, 210 mm, 152 mm. Also, NMW 94733, a pair of pharyngeal bones. Preservation condition: good.

**Type locality.** Not clearly provided in the original description but apparently the Danube. The syntypes are from Vienna and from the Danube without specification.

**Etymology.** The species name is a Latin word for virgin or maiden, which serves both as adjective and substantive.

**Taxonomic status.** Commonly treated as a synonym of *Rutiluspigus* (Lacepède, 1803) in earlier literature (e.g., Berg 1912: 79; Kottelat 1997: 79); a valid species, *Rutilusvirgo* (Heckel, 1852), in most recent publications (Bogutskaya and Iliadou 2006: 294, Kottelat and Freyhof 2007: 247; many others).

**Distribution.** Danube drainage upriver of Iron Gate; most abundant in Save drainage (Freyhof and Kottelat 2008g).

**Conservation status.** IUCN: *Rutilusvirgo* is in the LC category (Freyhof and Kottelat 2008g). In the Red Data List of Austria (Wolfram and Mikschi 2007: 47) (as *R.pigusvirgo*) in the category 2, Critically Endangered (“stark gefährdet”).

**Figure 32.**
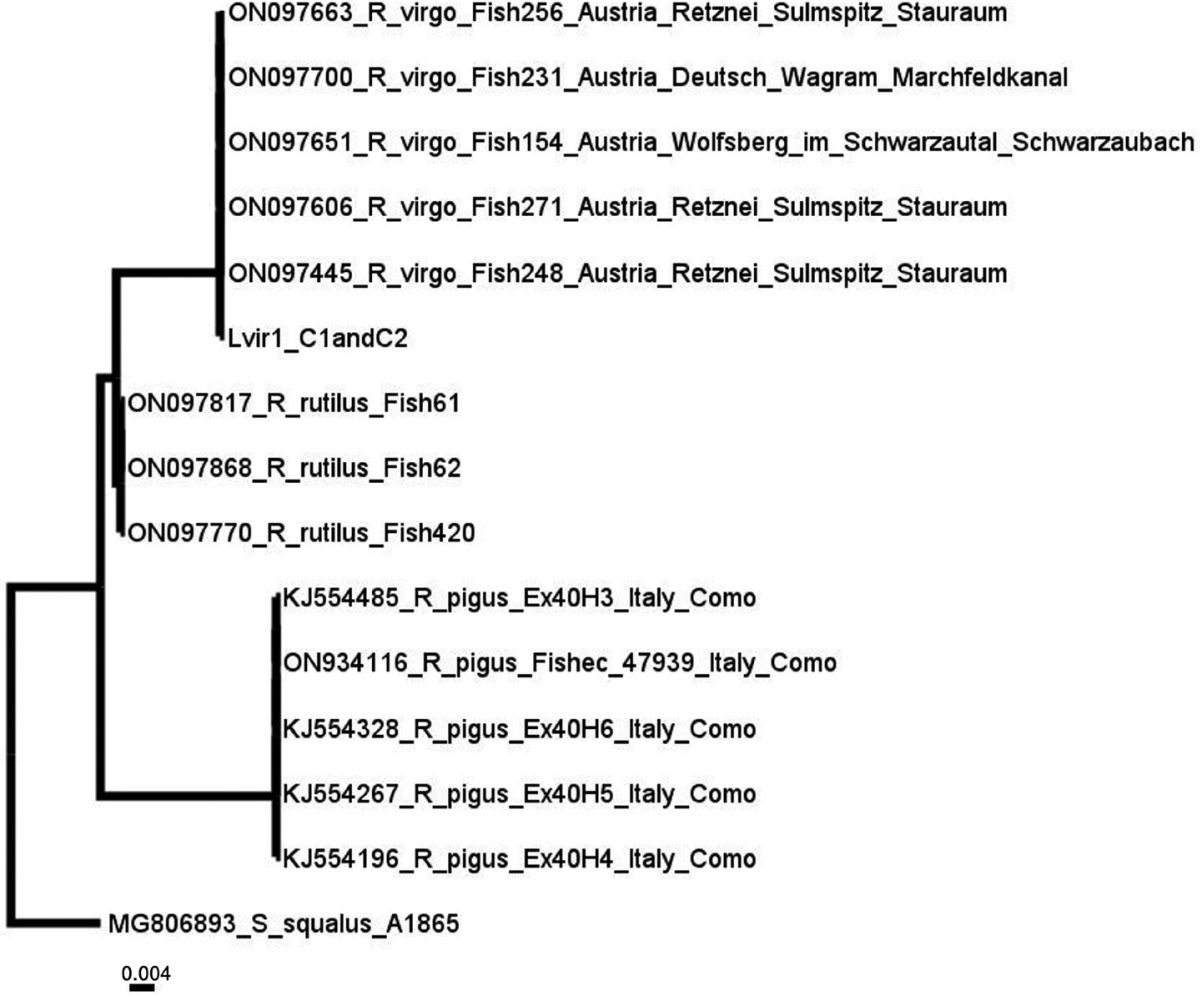
A simple neighbour-joining tree (MEGA 6.0; Tamura et al. (2013)) calculated based on a 218-bp long cytochrome oxidase I fragment and sequences downloaded from GenBank. Species names, localities, and accession numbers are given.

**Genetic information.** Two overlapping fragments of the mitochondrial COI region (LabID Lvir1; 217 bp in total, GenBank Accession No. PP576056) were successfully amplified in the specimen NMW 50626. The sequence is identical to the Austrian *R.virgo* sequences (Zangl et al. 2022) and clearly distant from both *Rutilusrutilus* (Linnaeus, 1758) and *R.pigus* (Fig. 32).

**10. *Phoxinusmarsilii* Heckel, 1836**

**Original publication of the name.**Heckel (1836: 232).

**Figure 33.**
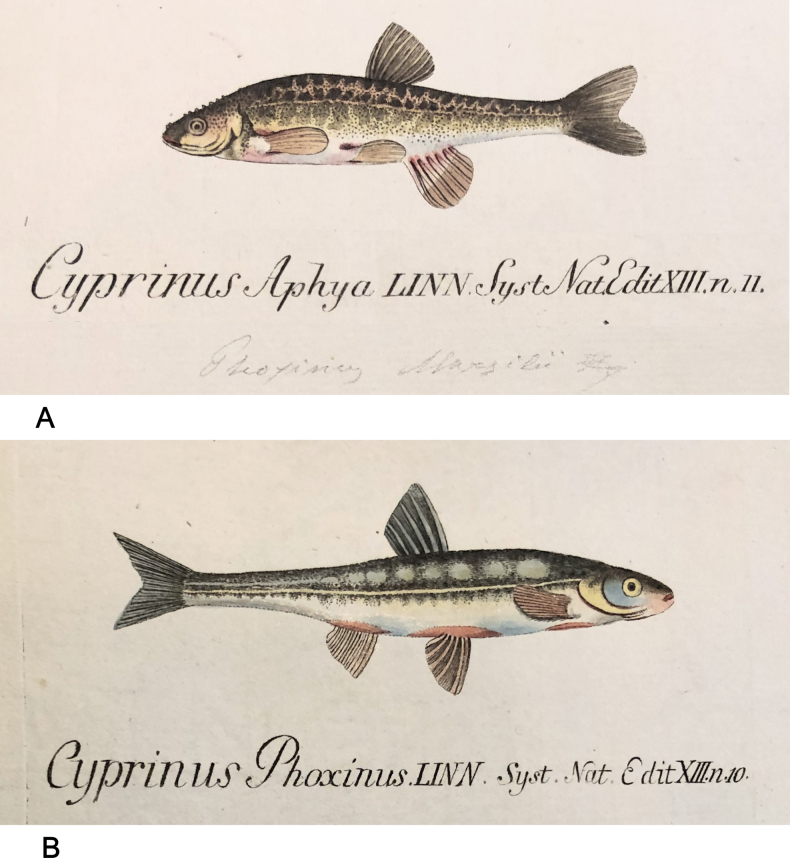
**A***Cyprinusaphya* from von Meidinger (1786: pl. XV); note the handwritten identification made by Heckel **B***Cyprinusphoxinus* from von Meidinger (1790: pl. XXXIX).

**Remarks.** The original description does not contain any specification of the examined specimens but makes clear that Heckle had examined (or observed) many. Heckel also refers to *Cyprinusaphya* of von Meidinger (1786: pl. XV) (Fig. 33a), *Cyprinusphoxinus* of von Meidinger (1790: pl. XXXIX) (Fig. 33b) and *Phoxinuslaevis* of Fitzinger (1832: 337).

He also provides a comparison of *Phoxinus* from the upper Danube in Germany (that could represent *P.csikii* in present understanding) and the new species: “Our museum owes many specimens of this species to the kindness of Professor Agassiz, who found them in Bavaria, and sent to the Cabinet Collection under the name *Phoxinuslaevis*. How closely this species approaches our local *PhoxinusMarsilii* in colour, from which it differs slightly by its larger scales and the lateral line that disappears in front of the tail, I do not dare to determine from specimens in alcohol; meanwhile, the black spot on the caudal fin is clearly visible, the back appears light brown with darker spots, the sides are mottled black along its length and the belly is silver; in terms of size they are at least 1/3 larger than the following [*P.marsilii*]”.

**Figure 34.**
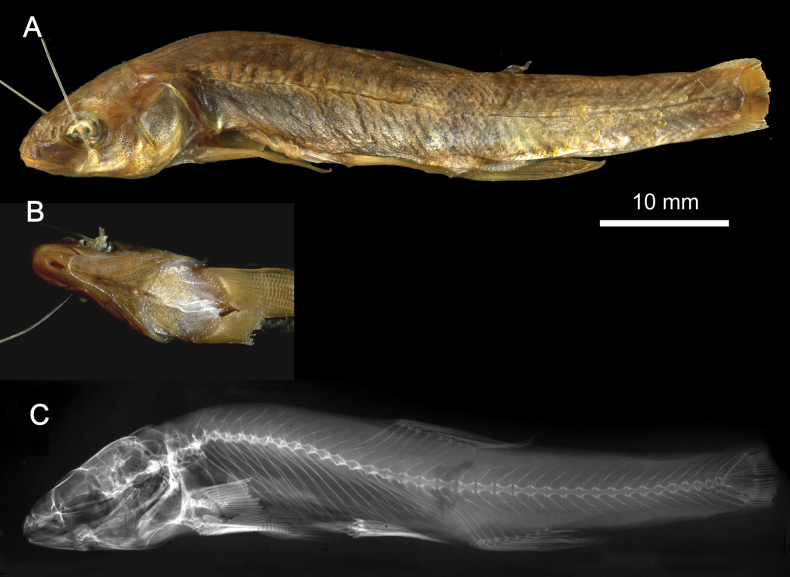
Lectotype of *Phoxinusmarsilii*, NMW 51225, lectotype, SL 65.5 mm, male, possibly, at Vienna **A** lateral view, **B** ventral view of head and breast to show a distinguishing feature of the species, continuous patches of breast scales (type 6 as defined in Bogutskaya et al. 2019: table 2) **C** radiograph.

**Lectotype.**NMW 51225, male (Fig. 34) (former 51225:2). Lectotype designated by Palandačić et al. (2017b: 2). Recent measurement of the lectotype: SL 65.5 mm. Preservation condition: bad (desiccated). Former syntypes (51225:1 and 51225:3–6), now paralectotypes, are NMW 98672.

The sample NMW 51225 was apparently registered (included into the inventory book) in Pietschmann’s time (judging from the number of the record and the handwriting) as belonging to the acquisition record 1836.I.20 for “*Phoxinusmarsilii* Donau”, but the locality was given as Vienna, possibly based on some information (e.g., labels that have been lost). However, there were only two (not six) individuals in the acquisition entry 1836.I.20 and the species name was given as already existing (known) that may indicate that the sample had been collected (received) after the species description. The six specimens which were considered syntypes, NMW 51225:1–6 (now 51225 and 98672) are in a very similar preservation condition (dried apparently long ago), so, all six specimens may belong to one and the same sample. We would assume that Heckel had seen all museum samples that were present in the collection before he described the species in 1836. These could be as follows.

1. 1825.IV.16 (eight specimens, bought from Laboratorium; possibly, Danube at Vienna or nearby).
2. 1826.VI.10 (two specimens, Moosbrunn in the south of Vienna);
3. 1830.II.5 for *Leuciscus Aphya*, 11 specimens (three from them were sent to Lüttich (Liege) on exchange) and 1830.II.6 for *Leuciscus Phoxinus*, ten specimens; in total, these acquisitions include seven samples (records) received from Leopold Fitzinger (see also the account on *Aspiusmento* above) and, apparently, the identifications were made by him; localities not specified but apparently the Danube.

**Type locality.** The original description does not specify neither examined individuals nor localities. However, it is clear from the context (Heckel 1836: 232–233) that the name is assigned to a *Phoxinus* from the Austrian part of the Danube: “Man findet unser Fischchen sehr häufig und in grossen Gesellschaften in allen klaren Bächen der Wiener-Gegend und weiter” (“Our little fish can be found very often and in large groups in all clear streams in the Vienna area and beyond”), which is compared with the Bavarian *P.laevis* (Heckel 1836: 232). As Heckel also refers to *Cyprinusaphya* and *Cyprinusphoxinus* of von Meidinger (1786: tab. XV and 1890: XXXIX, respectively) and *Phoxinuslaevis* of Fitzinger (1832: 337), he apparently defines the range of the species as, at least, Danube within the [former] Austrian Empire.

As shown above, the exact locality of the lectotype is not quite clear; it is still probable that it belongs to the acquisition 1836.I.20 and the locality is Vienna. Genetic analysis presented in Palanadačić et al. (2017a, 2020) shows that the same mitochondrial genetic lineage (lineage 9) has been distributed in Vienna in the last 200 years.

**Etymology.** The species name is a patronym, a noun in the genitive; named after Count Luigi Ferdinando Marsili (or Marsigli, Latin Marsilius; 1658–1730), an Italian scholar and natural scientist, an author of “Danubius Pannonico-Mysicus”, richly illustrated work in six volumes containing much valuable historic and scientific information on the river Danube (published in 1726).

**Taxonomic status.** Recently re-established as a valid species (Palandačić et al. 2017a); earlier, it was commonly treated as a synonym of *Phoxinusphoxinus* (Linnaeus, 1758).

**Distribution.** Danube drainage in Austria and Germany; also, Odra drainage in Germany (J. Freyhof, personal communication).

**Conservation status.** Not evaluated by IUCN. In the Red Data List of Lower Austria (Wolfram and Mikschi 2007: 91) (as *P.phoxinus*) in the category 4, Potentially Endangered (“potentiell gefährdet”); evaluation of the conservation status of all three *Phoxinus* species distributed in Austria according to most recent revisions (Palandačić et al. 2017a, 2020) is strongly required.

**Genetic information.** Three previously published partial sequences of the genes cytochrome b (cytb, MF408203), COI (MF407956) and internal transcribed spacer 1 (ITS1, MN818242).

**11. *Squaliusdelineatus* Heckel, 1843**

**Original publication of the name.**Heckel (1843: 1041).

**Remarks.** The original description is based on more than one individual (the number of lateral-line scales is given as a range, and two localities are mentioned).

**Figure 35.**
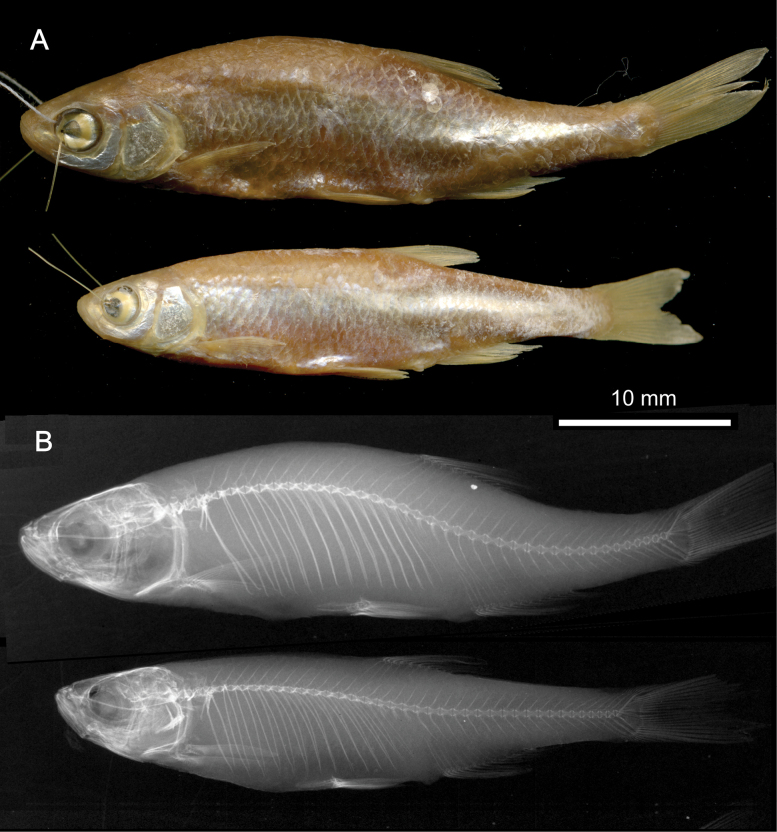
Syntypes of *Squaliusdelineatus*, NMW 50794:1, 3, Aderklaa **A** left lateral view **B** radiograph.

**Syntypes.**NMW 49783 (7) and 50794 (6) (Fig. 35), in alcohol, both from the acquisition record (made by Heckel) 1840.IX.4 (15 specimens, purchased from “Laboratorio”; Aderklaa); NMW 50796, in alcohol, acquisition record 1842.IV.33 (ten specimens (11 in the jar), Datschitz, Mähren [Dačice, Moravia]; NMW 94777 (a pair of pharyngeal bones, Vienna). Recent measurement of the syntypes (SL): NMW 49783: 57–59 mm; NMW 50794: 56.5–65 mm; NMW 50796: 39.5–67 mm. Preservation condition: average.

**Type locality.** In the original description as “in der Ebene des Marchfelds bei Wien, so wie auch in Mahren die einzelnen Feldlachen hautig bewonnt” (“in the plain of the Marchfeld near Vienna, as well as in Moravia”) (Heckel 1843: 1041). The Marchfeld is an area right in the north-east of Vienna on the left bank of the Danube; the locality Aderklaa belongs to this region (and is currently within the administrative boundaries of the city of Vienna).

**Etymology.** The species name is an adjective from Latin *delineatus*, past participle of *delineare* (to sketch out, from *de*- completely) + *lineare* (draw lines, from *linea* line), that refers to a peculiar feature of the species, a very shortened (reduced) lateral line.

**Taxonomic status.** A valid species since it was described, in a genus of its own, *Leucaspius* Heckel & Kner (1857: 145).

**Distribution.***Leucaspiusdelineatus* is native to Europe from lower Rhine and northern Germany eastward to southern Baltic basin, Black Sea basin south to Rioni drainage, Aegean Sea basin (from Maritsa to Nestos), and in northern Caspian basin; in Asia, native to western Caspian basin (south to Kura drainage). Introduced elsewhere (France, Great Britain, Switzerland, western Siberia in the Ob drainage in Russia and Kazakhstan) (Freyhof and Kottelat 2008h).

**Conservation status.** IUCN: *Leucaspiusdelineatus* is in the LC category (Freyhof and Kottelat 2008h). In the Red Data List of Lower Austria (Wolfram and Mikschi 2007: 70) in the category 3, Endangered (“gefährdet”).

**Genetic information.** Genetic analysis was not successful.

**12. *Squaliuslepusculus* Heckel, 1852**

**Original publication of the name.**Heckel (1852a: 109, pl. XII, figs 1–8).

**Figure 36.**
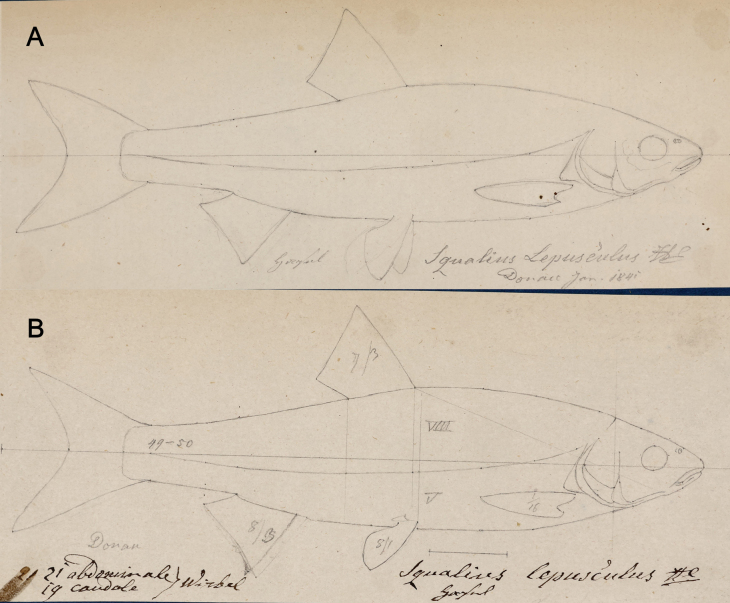
**A***Squaliuslepusculus*, original drafts by Heckel for the lithograph of the species presented in the original description (Heckel, 1852a: 109, pl. XII) and, later, in Heckel and Kner (1857: fig. 104) **B** a specimen collected in the Danube in January 1841 (NHMW Archive).

**Figure 37.**
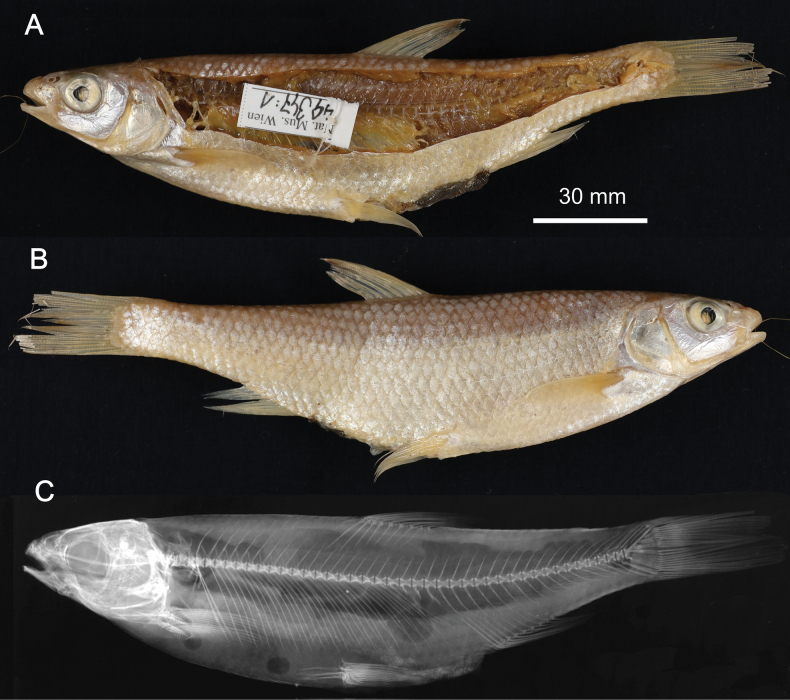
Possible syntype of *Squaliuslepusculus*, NMW 49347:1, SL 128.5 mm, Vienna **A** left and **B** right lateral views **C** radiograph.

**Syntypes.** The original description is mostly based on a single individual eight Viennese inches (= 158 mm) long (Heckel 1852a: 110, pl. XI, figs 1–4). However, there are clear indications that Heckel used more than one specimen for the description. First, two drafts by Heckel represent different fishes: one (Fig. 36a) is apparently taken from a real fish collected in the Danube in January 1841, and the other one (Fig. 36b) may be a composite as it contains the number of lateral-line scales as a range (49–50) and a note, added apparently later (in ink) “vertebrae 21 abdominal and 19 caudal”. Second, as the number of vertebrae is given, then, apparently, a specimen (or specimens) was/were dissected; indeed, there are two entirely laterally dissected specimens among the specimens considered syntypes of the species, NMW 49347:1 (Fig. 37) and 49359:2. Third, individuals NMW 49354:1, 59348, and 49359:1 lack the pharyngeal bones that may indicate that one of these specimens were used for the drawing of the pharyngeal bones (Heckel 1852a: pl. XI, fig. 3). NMW specimens historically labelled as syntypes of the species (all collected before 1852) are as follows: NMW 49345 (2), 49347 (2), 49348 (1), all three samples belong to the Acquisition 1825.IV.12–13, purchased from Laboratorio, Vienna; 49359 (2) and 49393 (2), both from the acquisition 1840.IX.8, purchased from Laboratorio, Moosbrunn (in the south of Vienna). Recent measurements (SL): NMW 49345 (2): 129 mm, 94 mm; 49347: 128.5 mm, 109 mm, 49348 (1): 146 mm; 49359 (2): 143 mm, 118.5 mm; and 49393 (2): 127 mm, 119 mm.

**Type locality.** Danube near Vienna and Moosbrun (defined by the possible syntypes as above). In the original description, the type locality is not specified per se; specimens seen by Heckel (including those deposited in the collection at the time) are from Upper Danube, Vienna, Vltava near České Budějovice, Olsa at Teschen (Cieszyn), upper reaches of the Elbe, and the Oder.

**Etymology.** The species name is a Latin masculine noun, diminutive *of lepus + -culus*, meaning a young hare, or leveret.

**Taxonomic status.** Synonymised with *Leuciscusleuciscus* (Linnaeus, 1758) (= *Leuciscusvulgaris* auct.) soon after the description (Günther 1868: 226).

**Distribution.***Leuciscusleuciscus* is native to North, Baltic, White, Barents, Caspian (Volga and Ural), Black Sea (Danube to Dnieper) basins (Freyhof 2011).

**Conservation status.** IUCN: *Leuciscusleuciscus* is in the LC category (Freyhof 2011). In the Red Data List of Lower Austria (Wolfram and Mikschi 2007: 118) as Not Endangered (“nicht gefährdet”)

**Genetic information.** Two overlapping fragments of the mitochondrial COI region (LabID Sleb1; 192 bp in total, GenBank Accession No. PP576057) were successfully amplified in the specimen NMW 49345:1. The sequence is identical to the *L.leuciscus* or *L.idus* (which based on COI sequences exhibit no differences) sequences from Austria (Zangl et al. 2022).

#### ﻿Aves type series

##### Aves: Anseriformes: Anatidae

**1. *Anserbrevirostris* Brehm, 1831**

**Original publication of the name.**Brehm (1831: 844).

**Syntypes.** 1. AMNH 730708, 2. RMNH 87330, 3. NHMW 55.170 additional 4. NHMW 20.928.

**Figure 38.**
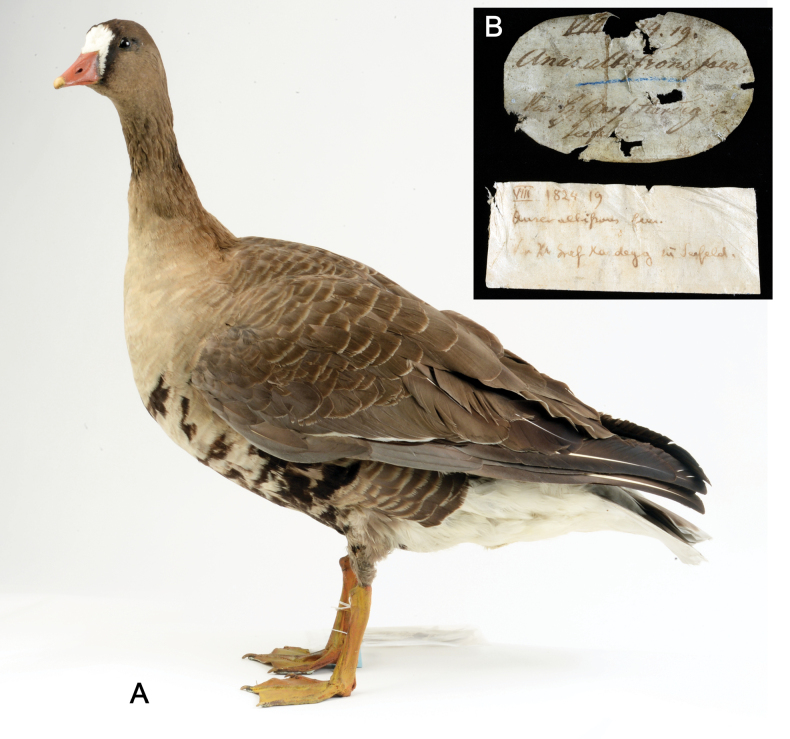
Syntype of *Anserbrevirostris***A** syntype of *Anserbrevirostris* “Heckel” C.L. Brehm, 1831, NMW 55.170 **B** corresponding original labels, removed from pedestal.

Syntypes in the bird collection Natural History Museum Vienna:

**NHMW 55.170** dry mounted (Fig. 38a); Acqu. No. 1824.VIII.19, female, adult; Seefeld; leg. et don. Graf Hardegg [Johann Dominik von Hardegg] (Fig. 38b); date of collecting is not given, presumably 1824. Preservation condition: good.

**Figure 39.**
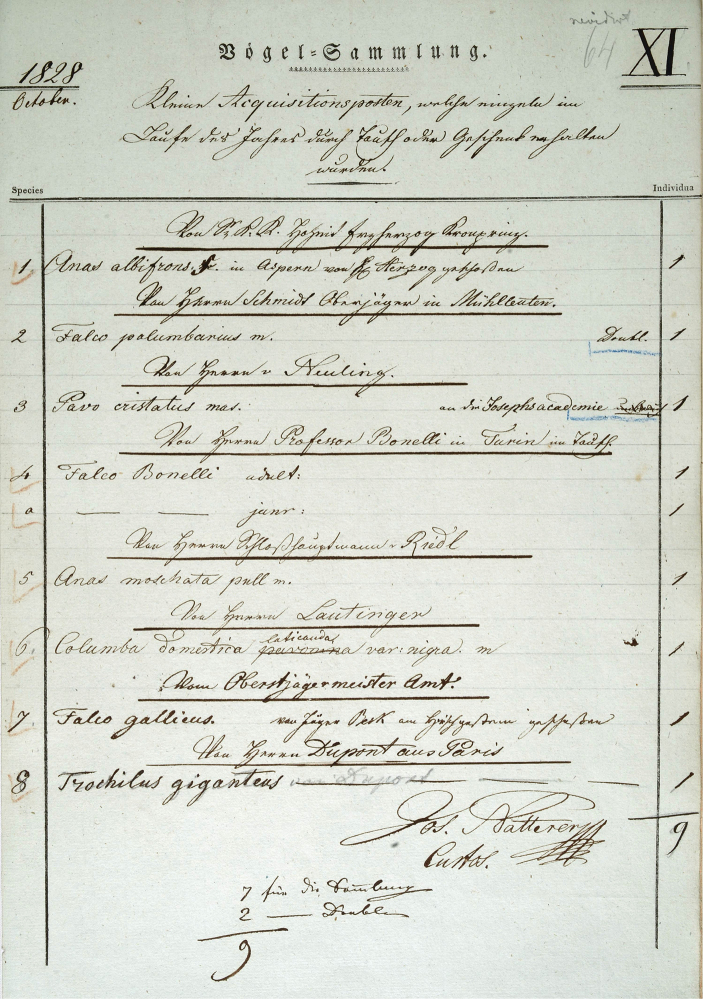
Acquisition Sheet “1828.XI”, record 1828.XI.1. Entry for year 1828 in the acquisition book of the bird collection/NHM Vienna for the specimen of *Ansererythropus* (here sub *Anasalbifrons*), NMW 20.928 (= syntype of *Anserbrevirostris* “Heckel” C.L. Brehm, 1831).

**NHMW 20.928** dry mounted; Acqu. No. 1828.XI.1 (Fig. 39), female; Aspern; leg.: shot by H. [Herrn] Herzog; don. Erzherzog Kronprinz [Ferdinand]; date of collecting is not given, presumably 1828; though Marschall and Pelzeln (1882) states 27.11.[1828]. Preservation condition: good.

**Remarks.** Syntype status of AMNH 730708, RMNH 87330, 3. NHMW 55.170 was confirmed by Mlikovsky (2023), syntype status of NHMW 20.928 confirmed by Schifter et al. (2007).

**Type locality.** 1. presumably Austria (from Vienna Market), 2. “Europe”, 3. Seefeld [Seefeld-Kadolz, Lower Austria; 48°43'N, 16°10'E]; 4. Aspern [48°13'N, 16°29'E Lower Austria; today 22^nd^ district of Vienna].

**Etymology.** The species name *brevirostris* is from Latin *brevis* (short), *rostrum* (beak).

**Taxonomic status.** Synonym of *Ansererythropus* (Linnaeus, 1758).

**Distribution.** Breeds in discontinuous narrow band across Arctic Eurasia from Norway to E Siberia. Winters from C and SE Europe east to Iran and in some regions of E Asia (Carboneras and Kirwan 2020).

**Conservation status.** In the IUCN category VU (BirdLife International 2018).

**Genetic information.** Two overlapping fragments of the mitochondrial COI region (LabID Aerythro; 220 bp in total, GenBank Accession No. PP576054) were successfully amplified in the specimen NHMW 55.170. Unfortunately, the sequence was not long enough to unambiguously connect the type specimen with a certain mitochondrial genetic lineage. Thus, further molecular analysis of the type(s) is needed.

## ﻿Conclusions

This catalogue presents and annotates historical type series of three parasitic worms, three myriapods, two insects, twelve fish, and one bird species with type locality in the state of Vienna. The catalogue includes historical information and the references to the literature in which they are mentioned, as well as photographs of specimens and their labels, scans of acquisition records, and radiographs where available. A total of 500 digital items have been produced, including the digitisation of 22 original descriptions, 17 drawings and illustrations, 64 acquisition books, registers, and labels, 52 catalogue cards, 91 radiographs, 241 image files, 12 short COI sequences, and one complete mitochondrial genome.

Genetic analysis was at least partially successful in 11 of the 21 type series, but only one extraction produced DNA of a quality that allowed shotgun sequencing, whereas in ten type series short fragments (100–230 bp) of COI were amplified and sequenced. The only existing *L.cavicola* syntype is already damaged and missing a leg, so genetic analysis was not attempted. Of the 27 specimens used for DNA extraction, genetic analysis provided at least some results in 13 specimens (48%), which is higher than previously reported for the NHMW Fish collection (Palandačić et al. 2020).

For the myriapod *Brachydesmussuperus*, the genetic analysis provided the first genetic information of this species in Austria and a genetic reference for the species name to be used in further (barcoding) projects. For the insect *Tetrixtuerki*, the COI fragment obtained was identical to the COIs originating from specimens collected from the Austrian-German border. For the fish species *Abramisschreibseri*, *Bliccaargyroleuca*, *Cyprinusacuminatus*, *Idusmelanotus*, and *Idusminiatus*, the genetic analysis confirmed their taxonomic status as synonyms of *Ballerussapa*, *Bliccabjoerkna*, *Cyprinuscarpio*, *Leuciscusidus*, and *L.idus*, respectively. For *Rutilusvirgo*, the genetic analysis confirmed the difference from *R.pigus* and the genetic identity with *R.virgo* recently collected in Austria (Zangl et al. 2022). For *Aspiusmento*, the DNA fragment obtained did not contain sufficient genetic information to resolve the taxonomic ambiguity associated with the lacustrine and riverine forms (for details see Results), of which the type series probably consists, and further research is therefore required. However, this study provides the first genetic record of *A.mento* in Austria, as this species was not included in the Austrian Barcode of Life project (Zangl et al. 2022). Similar to *A.mento*, the DNA fragment obtained from the bird species *Anserbrevirostris* did not contain sufficient genetic information to assign the type specimen to any of the currently valid *Anser* species.

Despite the partial success of the genetic analyses, this catalogue demonstrates the usefulness of ESA with the addition of genetic data. The catalogue contains digitised data from 21 type series, making them available to scientists around the world for further study.

## ﻿Additional information

### Author ORCIDs

Anja Palandačić https://orcid.org/0000-0002-4555-5240

Min J. Chai https://orcid.org/0009-0003-6213-2973

Nesrine Akkari https://orcid.org/0000-0001-5019-4833

Pedro R. Frade https://orcid.org/0000-0002-4010-255X

Susanne Randolf https://orcid.org/0000-0002-2402-3077

Nina G. Bogutskaya https://orcid.org/0000-0002-9153-0095

### Data availability

All of the data that support the findings of this study are available in the main text.

## References

[B1] Agatha S Ganser MH Santoferrara LF (2021) The importance of type species and their correct identification: A key example from tintinnid ciliates (Alveolata, Ciliophora, Spirotricha). The Journal of Eukaryotic Microbiology 68(6): e12865. 10.1111/jeu.12865 34243218

[B2] Agne S Naylor GJP Preick M Yang L Thiel R Weigmann S Paijmans JLA Barlow A Hofreiter M Straube N (2022a) Taxonomic identification of two poorly known lantern shark species based on mitochondrial DNA from wet-collection paratypes. Frontiers in Ecology and Evolution 10: 910009. 10.3389/fevo.2022.910009

[B3] Agne S Preick M Straube N Hofreiter M (2022b) Simultaneous barcode sequencing of diverse museum collection specimens using a mixed RNA bait set. Frontiers in Ecology and Evolution 10: 909846. 10.3389/fevo.2022.909846

[B4] Ahnelt H Mikschi E (2008) The type of *Gobiussemilunaris* Heckel, 1837 (Teleostei: Gobiidae). Annalen des Naturhistorischen Museums in Wien 109B: 67–72. https://www.zobodat.at/pdf/ANNA_109B_0067-0072.pdf

[B5] Akkari N Enghoff H Metscher BD (2015) A new dimension in documenting new species: High-detail imaging for myriapod taxonomy and first 3D cybertype of a new millipede species (Diplopoda, Julida, Julidae). PLOS ONE 10(8): e0135243. 10.1371/journal.pone.0135243 PMC4550252 26309113

[B6] Akkari N Ganske A-S Komerički A Metscher B (2018) New avatars for Myriapods: Complete 3D morphology of type specimens transcends conventional species description (Myriapoda, Chilopoda). PLOS ONE 13(7): e0200158. 10.1371/journal.pone.0200158 PMC6029791 29969504

[B7] Albano PG Schnedl S-M Eschner A (2018) An illustrated catalogue of Rudolf Sturany’s type specimens in the Naturhistorisches Museum Wien, Austria (NHMW): Deep-sea Eastern Mediterranean molluscs. Zoosystematics and Evolution 94(1): 29–56. 10.3897/zse.94.20116 30416602 PMC6225989

[B8] Anonymous (1857a) Nekrolog. Johan Jacob Heckel. Verhandlungen und Mitteilungen des Siebenbürgischen Vereins für Naturwissenschaften zu Hermannstadt 8: 119–124. https://www.zobodat.at/pdf/VerhMittNaturwissHermannstadt_8_0119-0124.pdf

[B9] Anonymous (1857b) [Herr Prof. Dr. R. Kner übergab ein Exemplar des von ihm in Verbindung mit dem verewigten Custos J. Heckel herausgegebenen Werkes]. Sitzungsberichte – Verhandlungen der Zoologisch-Botanischen Gesellschaft in Wien 7: 3–163. https://www.zobodat.at/pdf/VZBG_7_0003-0163.pdf

[B10] Antić D Akkari N (2020) *Haasea* Verhoeff, 1895—a genus of tumultuous history and chaotic records—redefinition, revision of taxonomy and geographic distributions, with descriptions of two new species from Austria and Serbia (Diplopoda, Chordeumatida, Haaseidae). Zootaxa 4798(1): 001–077. 10.11646/zootaxa.4798.1.1 33056685

[B11] Antognazza CM Palandaćić A Delmastro GB Crosa G Zaccara S (2023) Current and historical genetic variability of native brown trout populations in a southern Alpine ecosystem: Implications for future management. Fishes 8(8): 411. 10.3390/fishes8080411

[B12] Attems CMT Graf von (1908) Myriopoden von Elba. Zoologische Jahrbücher, Abteilung für Systematik, Ökologie und Geographie der Tiere 26(2): 181–195. https://www.biodiversitylibrary.org/partpdf/75062

[B13] Attems CMT Graf von (1927) Über palaearktische Diplopoden. Archiv für Naturgeschichte 92(1–2): 1–256. https://www.zobodat.at/pdf/Archiv-Naturgeschichte_92A_1_0001-0144.pdf

[B14] Balon EK (1995) Origin and domestication of the wild carp, *Cyprinuscarpio*: From Roman gourmets to the swimming flowers. Aquaculture (Amsterdam, Netherlands) 129(1–4): 3–48. 10.1016/0044-8486(94)00227-F

[B15] Bauernfeind E (2003) The Vienna Bird Collection: History and Main Research Focus. Bonn Zoological Bulletin 51: 147–149. https://www.zobodat.at/pdf/Bonner-Zoologische-Beitraege_51_0147-0149.pdf

[B16] Berg LS (1912) Faune de la Russie et des pays limitrophes fondés principalements sur les collections du Musée Zoologique de l’Académie Impériale des Sciences de St.-Pétersbourg. Poissons (Marsipobranchii et Pisces). Vol. III. Ostariophysi. Part 1. Izdanije Imperatorskoj Akademii Nauk, St. Petersbourg, 336 pp. https://archive.org/details/faunedelarussiee1191berg [In Russian]

[B17] Berg LS (1916) Les Poissons des eaux douces de la Russie. Ryabushinskiy, Moscow, 563 pp. [In Russian]

[B18] Berg H-M (2016) Die Vogelsammlung im Naturhistorischen Museum Wien. Vogelschutz in Österreich. Mitteilungen von BirdLife Österreich 40: 20–21.

[B19] BirdLife International (2018) *Ansererythropus*. The IUCN Red List of Threatened Species 2018: e.T22679886A132300164. 10.2305/IUCN.UK.2018-2.RLTS.T22679886A132300164.en [Accessed on 19 December 2023]

[B20] Bloch ME (1782) D. Marcus Elieser Bloch’s, ausübenden Arztes zu Berlin;…, Oeconomische Naturgeschichte der Fische Deutschlands. Mit Sieben und Dreissig Kupfertafeln nach Originalen. Theil 1. Auf Kosten des Verfassers und in Commission bei dem Buchhändler Hr. Hesse, Berlin, 128 pp. https://archive.org/details/DMarcusElieserB00BlocC/page/n5/mode/2up

[B21] Bogutskaya NG Iliadou K (2006) *Rutiluspanosi*, a new roach from western Greece (Teleostei: Cyprinidae). Zoosystematica Rossica 14(2): 293–298. 10.31610/zsr/2005.14.2.293

[B22] Bogutskaya NG Naseka AM (2004) Catalogue of agnathans and fishes of fresh and brackish waters of Russia with comments on nomenclature and taxonomy. KMK Scientific Press, Moscow, 389 pp. [In Russian]

[B23] Bogutskaya NG Zupančič P Jelić D Diripasko OA Naseka AM (2017) Description of a new species of *Alburnus* Rafinesque, 1820 (Actinopterygii, Cyprinidae, Leuciscinae) from the Kolpa River in the Sava River system (upper Danube drainage), with remarks on the geographical distribution of shemayas in the Danube. ZooKeys 688: 81–110. 10.3897/zookeys.688.11261 PMC5704097 29358895

[B24] Bogutskaya NG Jelić D Vucić M Jelić M Diripasko OA Stefanov T Klobučar G (2019) Description of a new species of *Phoxinus* from the upper Krka River (Adriatic Basin) in Croatia (Actinopterygii: Leuciscidae), first discovered as a molecular clade. Journal of Fish Biology 96(2): 378–393. 10.1111/jfb.14210 31750931

[B25] Bogutskaya NG Mikschi E Riedl M Szeiler S Frade P Palandačić A (2022) An annotated catalogue of the type specimens described by Maximilian Holly housed in the Natural History Museum of Vienna. Part. 1. Chordata. Annalen des Naturhistorischen Museums in Wien 124B: 19–92. https://www.zobodat.at/pdf/ANNA_124B_0019-0092.pdf

[B26] Böhm LK Supperer R (1953) Beobachtungen über eine neue Filarie (Nematoda), *Wehrdikmansiarugosicauda* Böhm and Supperer, 1953, aus dem subkutanen Bindegewebe des Rehes. Sitzungsberichte der Akademie der Wissenschaften Mathematisch-Naturwissenschaftliche Klasse 162: 95–104. https://www.zobodat.at/pdf/SBAWW_162_0095-0104.pdf

[B27] Braun M (1883) Zur Entwicklungsgeschichte des breiten Bandwurms (Bothriocephalus latus Brehms). A. Stuber, Würzburg, 72 pp. https://babel.hathitrust.org/cgi/pt?id=uc1.b3371776&seq=3

[B28] Braun M (1901) Zur Revision der Trematoden der Vögel. II. Centralblatt für Bakteriologie. Parasitenkunde und Infektionskrankheiten 29(23): 941–948.

[B29] Braun M Lühe M (1910) A handbook of practical parasitology. Translated by L. Forter. John Bale, Sons & Danielson, London, 208 pp. 10.5962/bhl.title.27217

[B30] Brehm CL (1831) Handbuch der Naturgeschichte aller Vögel Deutschlands. Bernhard Friedrich Voigt, Ilmenau, 844 pp. 10.5962/bhl.title.169254

[B31] Brehm CL (1855) Der vollständige Vögelsang: Eine gründliche Anleitung alle europaischen Vögel. B. Voigt, Weimar, 416 pp. 10.5962/bhl.title.15791

[B32] Brisout de Barneville L (1848) Catalogue des Acrididés qui se trouvent aux environs de Paris. Annales de la Société Entomologique de France 6: 411–445. https://www.biodiversitylibrary.org/page/9485831#page/417/mode/1up

[B33] Brölemann HW (1896) Matériaux pour servir a une faune des myriapodes de France. Feuille des jeunes naturalistes 26(311): 214–218.

[B34] Brunner B (1914) Zur Erinnerung an Carl Brunner von Wattenwyl (1823–1914). https://hls-dhs-dss.ch/de/articles/028793/2003-01-30/

[B35] Carboneras C Kirwan GM (2020) Lesser White-fronted Goose (*Ansererythropus*), version 1.0. In: del Hoyo J, Elliott A, Sargatal J, Christie DA, de Juana E (Eds) Birds of the World. Cornell Lab of Ornithology, Ithaca, NY, USA. 10.2173/bow.lwfgoo.01 [Accessed on 19 December 2023]

[B36] Carus JV (1880) Heckel, Johann Jakob. In: Allgemeine Deutsche Biographie (ADB). Band 11, Duncker und Humblot, Leipzig, 205. https://de.wikisource.org/wiki/ADB:Heckel,_Johann_Jakob

[B37] Castañeda-Rico S Edwards CW Hawkins MTR Maldonado JE (2022) Museomics and the holotype of a critically endangered cricetid rodent provide key evidence of an undescribed genus. Frontiers in Ecology and Evolution 10: 930356. 10.3389/fevo.2022.930356

[B38] Christian E (2008) Höhlenheuschrecken - Zum Jubiläum einer Wortschöpfung. Die Höhle 59(1–4): 48–58. https://www.zobodat.at/pdf/Hoehle_059_0048-0058.pdf

[B39] Durette-Desset M-C Digiani MC (2010) Taxonomic revision of the type specimens of Ethiopian Nippostrongylinae (Nematoda) deposited at the Natural History Museum of London. Zootaxa 2494(1): 1–28. 10.11646/zootaxa.2494.1.1

[B40] Federhen S (2014) Type material in the NCBI Taxonomy Database. Nucleic Acids Research 43(D1): D1086–D1098. 10.1093/nar/gku1127 PMC4383940 25398905

[B41] Feigin CY Newton AH Doronina L Schmitz J Hipsley CA Mitchell KJ Gower G Llamas B Soubrier J Heider TN Menzies BR Cooper A O’Neill RJ Pask AJ (2017) Genome of the Tasmanian tiger provides insights into the evolution and demography of an extinct marsupial carnivore. Nature Ecology & Evolution 2(1): 182–192. 10.1038/s41559-017-0417-y 29230027

[B42] Fischer M Schönmann R Moschner I (1976) Das Naturhistorisches Museum in Wien und seine Geschichte. Annalen des Naturhistorischen Museums in Wien 80: 1–24. https://www.zobodat.at/pdf/ANNA_80_0001-0024.pdf

[B43] Fitzinger LJFJ (1832) Ueber die Ausarbeitung einer Fauna des Erzherzogthumes Öesterreich, nebst einer systematischen Aufzählung der in diesem Lande vorkommenden Säugethiere, Reptilien und Fische, als Prodrom einer Fauna derfelben. Beiträge zur Landeskunde Österreichs unter der Enns, Vienna 1: 280–340.

[B44] Fitzinger LJFJ (1856) Geschichte des kön. Hof-Naturalien-Cabinetes zu Wien. 1. Abtheilung. Älteste Periode bis zum Tode Kaiser Leopold II, 1792. Sitzungsberichte der Akademie der Wissenschaften mathematisch-naturwissenschaftliche Klasse 21: 433–479. https://www.zobodat.at/pdf/SBAWW_21_0433-0479.pdf

[B45] Freyhof J (2011) *Leuciscusleuciscus* (errata version published in 2016). The IUCN Red List of Threatened Species 2011: e.T11887A97808936. 10.2305/IUCN.UK.2008.RLTS.T11887A3312583.en [Accessed on 11 October 2023]

[B46] Freyhof J Kottelat M (2007) Review of the *Alburnusmento* species group with description of two new species (Teleostei: Cyprinidae). Ichthyological Exploration of Freshwaters 18(3): 213–225.

[B47] Freyhof J Kottelat M (2008a) *Ballerussapa*. The IUCN Red List of Threatened Species 2008: e.T135639A4168069. 10.2305/IUCN.UK.2008.RLTS.T135639A4168069.en [Accessed on 11 October 2023]

[B48] Freyhof J Kottelat M (2008b) *Alburnusalburnus* (errata version published in 2020). The IUCN Red List of Threatened Species 2008: e.T789A174775859. 10.2305/IUCN.UK.2008.RLTS.T789A174775859.en [Accessed on 11 October 2023]

[B49] Freyhof J Kottelat M (2008c) *Alburnusmento*. The IUCN Red List of Threatened Species 2008: e.T135634A4167016. 10.2305/IUCN.UK.2008.RLTS.T135634A4167016.en [Accessed on 11 October 2023]

[B50] Freyhof J Kottelat M (2008d) *Bliccabjoerkna* (errata version published in 2020). The IUCN Red List of Threatened Species 2008: e.T39270A174781952. 10.2305/IUCN.UK.2008.RLTS.T39270A174781952.en [Accessed on 11 October 2023]

[B51] Freyhof J Kottelat M (2008e) *Cyprinuscarpio*. The IUCN Red List of Threatened Species 2008: e.T6181A12559362. 10.2305/IUCN.UK.2008.RLTS.T6181A12559362.en [Accessed on 09 October 2023]

[B52] Freyhof J Kottelat M (2008f) *Leuciscusidus*. The IUCN Red List of Threatened Species 2008: e.T11884A3312021. 10.2305/IUCN.UK.2008.RLTS.T11884A3312021.en [Accessed on 06 November 2023]

[B53] Freyhof J Kottelat M (2008g) *Rutilusvirgo*. The IUCN Red List of Threatened Species 2008: e.T135722A4193052. 10.2305/IUCN.UK.2008.RLTS.T135722A4193052.en [Accessed on 09 October 2023]

[B54] Freyhof J Kottelat M (2008h) *Leucaspiusdelineatus*. The IUCN Red List of Threatened Species 2008: e.T11873A3311162. 10.2305/IUCN.UK.2008.RLTS.T11873A3311162.en [Accessed on 09 October 2023]

[B55] Freyhof J Kaya C Bayçelebi E Geiger MF Turan D (2018) Generic assignment of *Leuciscuskurui* Bogutskaya from the upper Tigris drainage, and a replacement name for *Alburnuskurui* Mangit and Yerli (Teleostei: Leuciscidae). Zootaxa 4410(1): 113–135. 10.11646/zootaxa.4410.1.6 29690159

[B56] Fries BF Ekström CU Sundevall CJ (1837) Skandinaviens fiskar, målade efter lefvande exemplar och ritade på sten af Wilh. von Wright, med text af B. Fr. Fries, och C. U. Ekström. (Pisces Scandinavie.... Versio Latina...), 3. Stockholm, 222 pp. http://hdl.handle.net/2077/29271

[B57] Gebhardt L (1964) Brehm, Christian Ludwig. In: Gebhardt L Die Ornithologen Mitteleuropas. 1747 bemerkenswerte Biographien vom Mittelalter bis zum Ende des 20. Jahrhunderts. Brühl, Giessen, 51–52.

[B58] Gemel R Gassner G Schweiger S (2019) Katalog der Typen der Herpetologischen Sammlung des Naturhistorischen Museums Wien. Annalen des Naturhistorischen Museums in Wien 121B(2018): 33–248. http://verlag.nhm-wien.ac.at/pdfs/121B_033248_Gemel.pdf

[B59] Goldstein PZ DeSalle R (2011) Integrating DNA barcode data and taxonomic practice: Determination, discovery, and description. BioEssays 33(2): 135–147. 10.1002/bies.201000036 21184470

[B60] Günther A (1868) Catalogue of the fishes in the British Museum. Catalogue of the Physostomi, containing the families Heteropygii, Cyprinidae, Gonorhynchidae, Hyodontidae, Osteoglossidae, Clupeidae, Chirocentridae, Alepocephalidae, Notopteridae, Halosauridae, in the collection of the British Museum, 7. Order of the Trustees, London, 512 pp. https://www.biodiversitylibrary.org/item/34522#page/11/mode/1up

[B61] Hamann G [Ed.] (1976) Die Geschichte der Wiener naturhistorischen Sammlungen bis zum Ende der Monarchie. Mit einem Kapitel über die Zeit nach 1919 von M. Fischer, I. Moschner u. R. Schönmann. Veröffentlichungen aus dem Naturhistorischen Museum in Wien, Neue Folge 13: 1–98. https://www.zobodat.at/publikation_volumes.php?id=33611

[B62] Hardisty AR Ellwood ER Nelson G Zimkus B Buschbom J Addink W Rabeler RK Bates J Bentley A Fortes JAB Hansen S Macklin JA Mast AR Miller JT Monfils AK Paul DL Wallis E Webster M (2023) Digital Extended Specimens: Enabling an Extensible Network of Biodiversity Data Records as Integrated Digital Objects on the Internet. Bioscience 72(10): 978–987. 10.1093/biosci/biac060 PMC9525127 36196222

[B63] Haston E Cubey R Harris D (2012) Data concepts and their relevance for data capture in large scale digitisation of biological collections. International Journal of Humanities and Arts Computing 6(1–2): 111–119. 10.3366/ijhac.2012.0042

[B64] Hawkins MTR Flores MFC McGowen M Hinckley A (2022) A comparative analysis of extraction protocol performance on degraded mammalian museum specimens. Frontiers in Ecology and Evolution 10: 984056. 10.3389/fevo.2022.984056

[B65] Heckel JJ (1836) Über einige neue, oder nicht gehörig unterschiedene Cyprinen, nebst einer systematischen Darstellung der europäischen Gattungen dieser Gruppe. Annalen des Wiener Museums der Naturgeschichte 1: 219–234, Taf. 19–21. https://www.zobodat.at/pdf/AWMN_1_0219-0234.pdf

[B66] Heckel JJ (1843) Ichthyologie. In: Russegger J, Reisen in Europa, Asien und Afrika mit besonderer Rücksicht auf die naturwissenschaftlichen Verhältnisse der betreffenden Länder, unternommen in den Jahren 1835 bis 1841. 1. Band, 2. Teil. E. Schweizerbart, Stuttgart, 991–1099. https://www.biodiversitylibrary.org/item/80448#page/529/mode/1up

[B67] Heckel JJ (1850) Bericht über das Vorkommen fossiler Fische zu Seefeld in Tirol und Monte Bolca im Venetianischen. Jahrbuch der k.k. geologischen Reichsanstalt 1(4): 696–701. https://www.zobodat.at/pdf/JbGeolReichsanst_001_0696-0701.pdf

[B68] Heckel JJ (1851a) Ueber die in den Seen Oberösterreichs vorkommenden Fische. Sitzungsberichte der kaiserlichen Akademie der Wissenschaften, mathematisch-naturwissenschaftliche Classe 6(2): 145–149. https://www.zobodat.at/pdf/SBAWW_06_0145-0248.pdf

[B69] Heckel JJ (1851b) Ueber die Ordnung der Chondrostei und die Gattungen *Amia*, *Cyclurus*, *Notaeus*. Sitzungsberichte der kaiserlichen Akademie der Wissenschaften, mathematisch-naturwissenschaftliche Classe 6(2): 219–224. https://www.zobodat.at/pdf/SBAWW_06_0145-0248.pdf

[B70] Heckel JJ (1851c) Bericht einer auf Kosten der kais. Akademie der Wissenschaften durch Oberösterreich nach Salzburg, München, Innsbruck, Botzen, Verona, Padua, Venedig und Triest unternommenen Reise. Sitzungsberichte der kaiserlichen Akademie der Wissenschaften, mathematisch-naturwissenschaftliche Classe 7(2): 281–333. https://www.zobodat.at/pdf/SBAWW_07_0281-0333.pdf

[B71] Heckel JJ (1851d) Stör-Arten der Lagunen bei Venedig. [Weitere Fortsetzung des Reiseberichtes]. Sitzungsberichte der kaiserlichen Akademie der Wissenschaften, mathematisch-naturwissenschaftliche Classe 7(4): 547–563. https://www.zobodat.at/pdf/SBAWW_07_0547-0563.pdf

[B72] Heckel JJ (1852a) Fortsetzung des im Julihefte 1851 enthaltenen Berichtes über eine, auf Kosten der kais. Akademie der Wissenschaften unternommene, ichthyologische Reise. Anhang III. Über die zu den Gattungen *Idus*, *Leuciscus* und *Squalius* gehörigen Cyprinen. Sitzungsberichte der kaiserlichen Akademie der Wissenschaften, mathematisch-naturwissenschaftliche Classe 9(1): 49–123. https://www.zobodat.at/pdf/SBAWW_9_0049-0123.pdf

[B73] Heckel JJ (1852b) Verzeichniss der Fische des Donaugebietes in der ganzen Ausdehnung des österreichischen Kaiserstaates. Verhandlungen der Zoologisch-Botanischen Vereins in Wien 2: 28–33. https://www.zobodat.at/pdf/VZBG_2_0001-0120.pdf

[B74] Heckel JJ Kner R (1857) Die Süsswasserfische der Österreichischen Monarchie, mit Rücksicht auf die angränzenden Länder. W. Engelmann, Leipzig, 388 pp. https://www.zobodat.at/pdf/MON-V-FISCH_0001_0001-0388.pdf

[B75] Herzig-Straschil B (1997) Franz Steindachner (1834–1919) and other prime contributors to the Ichthyological Collection of the Naturhistorisches Museum Wien. In: Pietsch TW Anderson Jr WD (Eds) Collection Building in Ichthyology and Herpetology.The American Society of Ichthyologists and Herpetologists, Special Publication 3(1), 101–108.

[B76] Hildebrandt HCM (1929) Christian Ludwig Brehm als Ornithologe. Mitteilungen aus dem Osterlande 20: 23–54.

[B77] Hochkirch A Nieto A García Criado M Cálix M Braud Y Buzzetti FM Chobanov D Odé B Presa Asensio JJ Willemse L Zuna-Kratky T Barranco Vega P Bushell M Clemente ME Correas JR Dusoulier F Ferreira S Fontana P García MD Heller K-G Iorgu IȘ Ivković S Kati V Kleukers R Krištín A Lemonnier-Darcemont M Lemos P Massa B Monnerat C Papapavlou KP Prunier F Pushkar T Roesti C Rutschmann F Şirin D Skejo J Szövényi G Tzirkalli E Vedenina V Barat Domenech J Barros F Cordero Tapi PJ Defaut B Fartmann T Gomboc S Gutiérrez-Rodríguez J Holuša J Illich I Karjalainen S Kočárek P Korsunovskaya O Liana A López H Morin D Olmo-Vidal JM Puskás G Savitsky V Stalling T Tumbrinck J (2016) European Red List of Grasshoppers, Crickets and Bush-crickets. Publications Office of the European Union, Luxembourg, 88 pp. https://op.europa.eu/en/publication-detail/-/publication/e2d74198-523d-11e7-a5ca-01aa75ed71a1/language-en

[B78] Hoeksema BW Koh EGL (2009) Depauperation of the mushroom coral fauna (Fungiidae) of Singapore (1860s–2006) in changing reef conditions. The Raffles Bulletin of Zoology 22(Supplement): 91–101.

[B79] Hoeksema BW van der Land J van der Meij SET van Ofwegen LP Reijnen BT van Soest RWM de Voogd NJ (2011) Unforeseen importance of historical collections as baselines to determine biotic change of coral reefs: The Saba Bank case. Marine Ecology (Berlin) 32(2): 135–141. 10.1111/j.1439-0485.2011.00434.x

[B80] Ilie V Schiller E Stagl V (2009) Type specimens of the Geophilomorpha (Chilopoda) in the Natural History Museum in Vienna. Kataloge der wissenschaftlichen Sammlungen des Naturhistorischen Museums in Wien. Band 22, Myriapoda Heft 4. Verlag des Naturhistorischen Museums Wien, Vienna, 75 pp.

[B81] International Commission on Zoological Nomenclature (1999) International Code of Zoological Nomenclature, Fourth Edition: adopted by the International Union of Biological Sciences. The International Trust for Zoological Nomenclature, London, 306 pp. https://code.iczn.org/?frame=1

[B82] International Union for Conservation of Nature (2012) IUCN Red List Categories and Criteria: Version 3.1. Second edition. IUCN, Gland, Switzerland and Cambridge, UK, 32 pp. https://portals.iucn.org/library/node/10315

[B83] Kähsbauer P (1959) Intendant Dr. Franz Steindachner, sein Leben und Werk. Annalen des Naturhistorischen Museums in Wien 63: 1–30. https://www.zobodat.at/pdf/ANNA_63_0001-0030.pdf

[B84] Kaltenbach AP (2001) Die Orthopterensammlung des Naturhistorischen Museums in Wien und ihre Geschichte. Entomologica Austriaca: Zeitschrift der Osterreichischen Entomologischen Gesellschaft 4: 21–23. https://www.zobodat.at/pdf/ENTAU_0004_0021-0023.pdf

[B85] Kehlmaier C Zinenko O Fritz U (2020) The enigmatic Crimean green lizard (*Lacertaviridismagnifica*) is extinct but not valid: Mitogenomics of a 120-year-old museum specimen reveals historical introduction. Journal of Zoological Systematics and Evolutionary Research 58(1): 303–307. 10.1111/jzs.12345

[B86] Kime RD Enghoff H (2017) Atlas of European millipedes 2: Order Julida (Class Diplopoda). European Journal of Taxonomy 346(346): 1–299. 10.5852/ejt.2017.346

[B87] Kleinschmidt A (1955) Brehm, Christian Ludwig. Neue Deutsche Biographie 2: 570. https://www.deutsche-biographie.de/pnd116469838.html#ndbcontent

[B88] Kollar V (1824) Monographia Chlamydum. Cum tabulis aeneis duabus coloratus. JG Heubner, Vienna, 49 pp. 10.5962/bhl.title.52095

[B89] Kollar V (1831a) Insekten des Schneebergs. In: Schmidl A. Der Schneeberg in Unterösterreich. Vienna, 36–41.

[B90] Kollar V (1831b) Über Insecten, als Ursache verschiedener Krankheiten bey Menschen und Thieren. Wiener Zeitschrift für Kunst, Literatur, Theater und Mode: 781–786, 792–795, 799–801.

[B91] Kollar V (1833a) Systematisches Verzeichnis der im Erzherzogthume Oesterreich vorkommenden geradflügeligen Insecten. Beiträge zur Landeskunde Oesterreichs unter der Enns, Vienna 3: 67–87.

[B92] Kollar V (1833b) Die Kornschabe, *Tineagranella* Lin. Verhandlungen der K.K. Landwirthschafts-Gesellschaft in Wien, und Aufsätze vermischten ökonomischen Inhaltes. Neue Folge 1(2): 52–59.

[B93] Kollar V (1837) Naturgeschichte der schädlichen Insecten in Beziehung auf Landwirthschaft und Forstcultur. Monografien Entomologie Gemischt 5: 1–421. https://www.zobodat.at/pdf/MON-E-DIV_0005_0001-0421.pdf

[B94] Kollar V (1842) Über einige dem Feld- und Gartenbau verderbliche Insecten. Verhandlungen der K.K. Landwirthschafts-Gesellschaft in Wien, und Aufsätze vermischten ökonomischen Inhaltes. Neue Folge 11(2): 125–148.

[B95] Kollar V (1850) Ueber Weinbeschädigung durch einen kleinen Nachtfalter, *Tortrix* Roserana Fröhl., in den Weingärten von Brunn nächst Mödling. Sitzungsberichte der kaiserlichen Akademie der Wissenschaften, mathematisch-naturwissenschaftliche Classe 5: 89–91. https://www.zobodat.at/pdf/SBAWW_05_0001-0093.pdf

[B96] Kollar V (1855) Ueber Beschädigung des Roggens durch *Apameabasilinea* W.V. (mit Abbild.). Verhandlungen des Zoologisch-Botanischen Vereins in Wien 5: 697–700. https://www.zobodat.at/pdf/VZBG_5_0697-0700.pdf

[B97] Kottelat M (1997) European freshwater fishes. Biologia 52(suppl. 5): 1–271.

[B98] Kottelat M Freyhof J (2007) Handbook of European freshwater fishes. Publications Kottelat, Cornol, Switzerland, 646 pp.

[B99] Krauss HA (1876) *Tettix* Türki nov. spec. (Orthopt.). Entomologische Monatsblätter: 103–104.

[B100] Krauss HA (1878) [1879] Die Orthopteren-Fauna Istriens. Sitzungsberichte der kaiserlichen Akademie der Wissenschaften, mathematisch-naturwissenschaftliche Classe 78(1): 451–544. https://www.zobodat.at/pdf/SBAWW_78_0451-0544.pdf

[B101] Kress JW Erickson DL (2012) DNA barcodes: methods and protocols. In: Kress JW Erickson DL (Eds) Methods in Molecular Biology, vol.858. Springer Science+Business Media, 4–8. 10.1007/978-1-61779-591-6_3 22684949

[B102] Lacepède BGE (1803) Histoire naturelle des poissons. Vol. 5. F. Dufart, Paris, 1–803. https://archive.org/details/histoirenaturel1lacea

[B103] Latzel R (1884) Die Myriopoden der Österreichisch-ungarischen Monarchie. Zweite Hälfte. Die Symphylen, Pauropoden und Diplopoden. A. Hölder, Vienna, 414 pp.

[B104] Lefoulon E Kuzmin Y Plantard O Mutafchiev Y Otranto D Martin C Bain O (2014) Redescription of *Cercopithifilariarugosicauda* (Böhm and Supperer, 1953) (Spirurida: Onchocercidae) of roe deer, with an emended diagnosis of the genus *Cercopithifilaria* and a genetic characterisation. Parasitology International 63(6): 808–816. 10.1016/j.parint.2014.07.011 25108130

[B105] Lendemer J Thiers B Monfils AK Zaspel J Ellwood ER Bentley A LeVan K Bates J Jennings D Contreras D Lagomarsino L Mabee P Ford LS Guralnick R Gropp RE Revels M Cobb N Seltmann K Aime MC (2020) The extended specimen network: A strategy to enhance US biodiversity collections, promote research and education. Bioscience 70(1): 23–30. 10.1093/biosci/biz140 31949317 PMC6956879

[B106] Li C Corrigan S Yang L Straube N Harris M Hofreiter M White WT Naylor GJP (2015) DNA capture reveals transoceanic gene flow in endangered river sharks. Proceedings of the National Academy of Sciences of the United States of America 112(43): 13302–13307. 10.1073/pnas.1508735112 26460025 PMC4629339

[B107] Linnaeus C (1758) Systema Naturae, Ed. X. (Systema naturae per regna tria naturae, secundum classes, ordines, genera, species, cum characteribus, differentiis, synonymis, locis. Tomus I. Editio decima, reformata.), 1. Laurentii Salvii, Holmiae, 824 pp. 10.5962/bhl.title.542

[B108] Marschall AF Graf Pelzeln A von (1882) Ornis Vindobonensis. Die Vogelwelt Wiens und seiner Umgebung. Georg Paul Faesy, Vienna, 192 pp.

[B109] Maxted N (1992) Towards defining a taxonomic revision methodology. Taxon 41(4): 653–660. 10.2307/1222391

[B110] Meineke EK Davies TJ Daru BH Davis CC (2018) Biological collections for understanding biodiversity in the Anthropocene. Philosophical Transactions of the Royal Society B: Biological Sciences 374(1763): 20170386. 10.1098/rstb.2017.0386 PMC6282082 30455204

[B111] Mikschi E (2009) Geschichte der Fischforschung am Naturhistorischen Museum. Österreichs Fischerei 62: 292–296.

[B112] Mlikovsky J (2023) Systematic Catalogue of the Birds of Siberia. Historical Ornithology, vol. 1. Center for Historical Ornithology, Praha, 3167 pp.

[B113] Monfils AK Krimmel ER Linton DL Marsico TD Morris AB Ruhfel BR (2022) Collections education: The extended specimen and data acumen. Bioscience 72(2): 177–188. 10.1093/biosci/biab109 35145351 PMC8824687

[B114] Moog O (1982) Die Verbreitung der Höhlenheuschrecken *Troglophiluscavicola* Kollar und *T.neglectus* Krauss in Österreich (Orthoptera, Rhaphidophoridae). Sitzungsberichte der Akademie der Wissenschaften, mathematisch-naturwissenschaftliche Klasse 191: 185–207. https://www.zobodat.at/pdf/SBAWW_191_0185-0207.pdf

[B115] Palandačić A Naseka AM Ramler D Ahnelt H (2017a) Contrasting morphology with molecular data: An approach to revision of species complexes based on the example of European *Phoxinus* (Cyprinidae). BMC Evolutionary Biology 17(184): 1–17. 10.1186/s12862-017-1032-x 28793855 PMC5549366

[B116] Palandačić A Naseka AM Ramler D Ahnelt H (2017b) Corrigendum to «Contrasting morphology with molecular data: an approach to revision of species complexes based on the example of European (Cyprinidae)» by Palandačić et al. 2017. Biodiversity Data Journal 5: e21772[1–5]. 10.3897/BDJ.5.e21772 PMC5665002 29104443

[B117] Palandačić A Kruckenhauser L Ahnelt H Mikschi E (2020) European minnows through time: museum collections aid genetic assessment of species introductions in freshwater fishes (Cyprinidae: *Phoxinus* species complex). Heredity 124(3): 410–422. 10.1038/s41437-019-0292-1 31896822 PMC7028953

[B118] Palandačić A Kapun M Greve C Schell T Kirchner S Kruckenhauser L Szucsich N Bogutskaya NG (2023) From historical expedition diaries to whole genome sequencing: A case study of the likely extinct Red Sea torpedo ray. Zoologica Scripta 00: 1–20. 10.1111/zsc.12632

[B119] Pallas PS (1814) Zoographia Rosso-Asiatica, sistens omnium animalium in extenso Imperio Rossico et adjacentibus maribus observatorum recensionem, domicilia, mores et descriptiones anatomen atque icones plurimorum. Vol. 3. Academia Scientiarum, Petropolis (Sankt Petersburg), 428 pp. https://archive.org/details/zoographiarossoa22pall

[B120] Pohl JE Kollar V (1832) Brasiliens vorzüglich lästige Insecten. In: Reise im Inneren von Brasilien. Auf allerhöchsten Befehl Seiner Majestät des Kaisers von Österreich Franz des Ersten in den Jahren 1817–1821: 1–20.

[B121] Price B Dupont S Allan E Blagoderov V Butcher A Durrant J Holtzhausen P Kokkini P Livermore L Hardy H Smith V (2018) . ALICE: Angled Label Image Capture and Extraction for high throughput insect specimen digitisation. 10.31219/osf.io/s2p73

[B122] Prosser SWJ deWaard JR Miller SE Hebert PDN (2016) DNA barcodes from century-old type specimens using next-generation sequencing. Molecular Ecology Resources 16(2): 487–497. 10.1111/1755-0998.12474 26426290

[B123] Rabitsch W (2006) Geschichte und Bibliographie der Wanzenkunde in Österreich Denisia 19: 41–94. https://www.zobodat.at/pdf/DENISIA_0019_0041-0094.pdf

[B124] Rambur P (1838) Orthoptères. In: Rambur P Faune entomologique de l’Andalousie: Deux forts volumes in octavo accompagnés de 50 planches. Vol. 2. A. Bartrand, 12–94.

[B125] Raxworthy CJ Smith BT (2021) Mining museums for historical DNA: Advances and challenges in museomics. Trends in Ecology & Evolution 36(11): 1049–1060. 10.1016/j.tree.2021.07.009 34456066

[B126] Renner SS (2016) A return to Linnaeus’s focus on diagnosis, not description: The use of DNA characters in the formal naming of species. Systematic Biology 65(6): 1085–1095. 10.1093/sysbio/syw032 27146045

[B127] Richardson J (1846) Report on the ichthyology of the seas of China and Japan. Report of the British Association for the Advancement of Science 15^th^ meeting [1845]: 187–320. https://archive.org/details/reportonichthyol00rich

[B128] Robbirt KM Roberts DL Hutchings MJ Davy AJ (2014) Potential disruption of pollination in a sexually deceptive orchid by climatic change. Current Biology 24(23): 2845–2849. 10.1016/j.cub.2014.10.033 25454786

[B129] Saint Quentin D (1970) Katalog der Odonaten-Typen im Naturhistorischen Museum Wien. Annalen des Naturhistorischen Museums in Wien 74: 253–279. https://www.zobodat.at/pdf/ANNA_74_0253-0279.pdf

[B130] Sattmann H (2002) Anfänge der systematischen Helminthologie in Österreich. Denisia 6: 271–290. https://www.zobodat.at/pdf/DENISIA_0006_0271-0290.pdf

[B131] Sattmann H Konecny R Stagl V (2000) Die Geschichte der Helminthensammlung am Natur-historischen Museum in Wien, Teil 1 (1797–1897). Mitteilungen der Österreichischen Gesellschaft für Tropenmedizin und Parasitologie 21(1999): 83–92. https://www.zobodat.at/pdf/MOGTP_21_0083-0092.pdf

[B132] Sattmann H Stagl V Esberger R Konecny R (2001) Die Geschichte der Helminthensammlung am Naturhistorischen Museum in Wien, Teil 2 (1898–1998). Mitteilungen der Österreichischen Gesellschaft für Tropenmedizin und Parasitologie 22(2000): 25–32. https://www.zobodat.at/pdf/MOGTP_22_0025-0032.pdf

[B133] Schefbeck G (1996) The Austro-Hungarian Deep-sea Expeditions. In: Uiblein F Ott J Stachowitsch M (Eds) Deep-sea and extreme shallow-water habitats: affinities and adaptations.Österreichische Akademie der Wissenschaften, Biosystematics and Ecology Series 11: 1–27.

[B134] Schifter H (1991) Typen von Theodor von Heuglin beschriebener Vögel in der Vogelsammlung des Naturhistorischen Museums Wien. Annalen des Naturhistorischen Museums in Wien 92B: 59–76. https://www.zobodat.at/pdf/ANNA_92B_0059-0076.pdf

[B135] Schifter H (2010) Specimens in the public galleries reflecting the history of the Bird Collection at the Museum of Natural History in Vienna. In: Bauernfeind E Gamauf A Berg H-M Muraoka Y (Eds) Proceedings of the 5th International Meeting of European Bird Curators, Nat.Hist. Mus. Vienna, Aug. 29^th^–31^st^, 2007. Publishing House of the Natural History Museum Vienna, Vienna, 237–255.

[B136] Schifter H Bauernfeind E Schifter T (2007) : Die Typen der Vogelsammlung des Naturhistorischen Museums Wien. Teil I. Nonpasseres. Katalog der wissenschaftlichen Sammlungen des Naturhistorischen Museums in Wien, Band 20. Aves Heft I. Naturhistorischen Museums, Vienna, 376 pp. https://www.zobodat.at/pdf/kat-nhmw_20_0003-0376.pdf

[B137] Schileyko A Stagl V (2004) The collection of Scolopendromorph Centipedes (Chilopoda) in the Natural History Museum in Vienna: A critical re-evaluation of former taxonomic identifications. Annalen des Naturhistorischen Museums in Wien 105B: 67–137. https://www.zobodat.at/pdf/ANNA_105B_0067-0137.pdf

[B138] Schiner IJR (1860) Vincenz Kollar. Wiener Entomologische Monatschrift 4: 222–224.

[B139] Schrötter A (1861) Vincenz Kollar. Almanach der kaiserlichen Akademie der Wissenschaften 11: 154–169.

[B140] Silva PC Malabarba MC Malabarba LR (2017) Using ancient DNA to unravel taxonomic puzzles: the identity of *Deuterodonpedri* (Ostariophysi: Characidae). Neotropical Ichthyology 15(1): 1–12. 10.1590/1982-0224-20160141

[B141] Silva PC Malabarba MC Malabarba LR (2019) Integrative taxonomy: Morphology and ancient DNA barcoding reveals the true identity of *Astyanaxtaeniatus*, a tetra collected by Charles Darwin during the Beagle’s Voyage. Zoologischer Anzeiger 278: 110–120. 10.1016/j.jcz.2018.12.007

[B142] Stagl V (2006) Robert Latzel - his life-work and importance for Myriapodology. Norwegian Journal of Entomology 53: 223–236.

[B143] Stagl V Sattmann H (2013) Der Herr der Würmer: Leben und Werk des Wiener Arztes und Parasitologen Johann Gottfried Bremser (1767–1827). Böhlau, Vienna, 240 pp. 10.7767/boehlau.9783205789857

[B144] Stagl V Stoev P (2005) Type specimens of the order Callipodida (Diplopoda) in the Natural History Museum in Vienna. Kataloge der wissenschaftlichen Sammlungen des NHMW. Myriapoda, Heft 2. Natural History Museum, Vienna, 26 pp.

[B145] Stagl V Zapparoli M (2006) Type specimens of the Lithobiomorpha (Chilopoda) in the Natural History Museum in Vienna. Kataloge der wissenschaftlichen Sammlungen des NHMW. Myriapoda, Heft 3. Natural History Museum, Vienna, 49 pp.

[B146] Stoev P Komerički A Akkari N Liu S Zhou X Weigand A Hostens J Hunter C Edmunds S Porco D Zapparoli M Georgiev T Mietchen D Roberts D Faulwetter S Smith V Penev L (2013) *Eupolybothruscavernicolus* Komerički and Stoev sp. n. (Chilopoda: Lithobiomorpha: Lithobiidae): the first eukaryotic species description combining transcriptomic, DNA barcoding and micro-CT imaging data. Biodiversity Data Journal 1: e1013. 10.3897/BDJ.1.e1013 PMC3964625 24723752

[B147] Straube N Lyra ML Paijmans JLA Preick M Basler N Penner J Rödel M-O Westbury MV Haddad CFB Barlow A Hofreiter M (2021) Successful application of ancient DNA extraction and library construction protocols to museum wet collection specimens. Molecular Ecology Resources 21(7): 2299–2315. 10.1111/1755-0998.13433 34036732

[B148] Sullivan JP Hopkins CD Pirro S Peterson R Chakona A Mutizwa TI Mukweze Mulelenu C Alqahtani FH Vreven E Dillman CB (2022) Mitogenome recovered from a 19^th^ century holotype by shotgun sequencing supplies a generic name for an orphaned clade of African weakly electric fishes (Osteoglossomorpha, Mormyridae). ZooKeys 1129: 163–196. 10.3897/zookeys.1129.90287 36761845 PMC9836601

[B149] Surdez M (1973) Catalogue des Archives de Louis Agassiz (1807–1873). Université de Neuchatel, Institut de Géologie et Séminaire d’Histoire, 196 pp. [Version informatisée mai 2009 par MDS, Archives de l’Etat de Neuchâtel] https://doc.rero.ch/record/32397/files/PAL_E3549.pdf

[B150] Svojtka M Salvini-Plawen L Mikschi E (2009) Biographischer Abriss zu Johann Jakob Heckel (1790–1857). Österreichs Fischerei 62(11/12): 285–288.

[B151] Svojtka M Salvini-Plawen L Mikschi E (2012) Johann Jakob Heckel (1790–1857), der Begründer der systematischen Ichthyologie in Österreich: Ein biographischer Überblick. Schriften des Vereins zur Verbreitung Naturwissenschaftlicher Kenntnisse 148/150: 43–74.

[B152] Takano A Cole TCH Konagai H (2024) A novel automated label data extraction and data base generation system from herbarium specimen images using OCR and NER. Scientific Reports 14(1): 112. 10.1038/s41598-023-50179-0 38167449 PMC10761843

[B153] Tamura K Stecher G Peterson D Filipski A Kumar S (2013) MEGA6: Molecular evolutionary genetics analysis version 6.0. Molecular Biology and Evolution 30(12): 2725–2729. 10.1093/molbev/mst197 24132122 PMC3840312

[B154] Thaler K Gruber J (2003) Zur Geschichte der Arachnologie in Österreich 1758–1955. Denisia 8: 139–163. https://www.zobodat.at/pdf/DENISIA_0008_0139-0163.pdf

[B155] Tizzani P Fanelli A Belleau E (2021) Gastrointestinal parasites of black grouse *Lyrurustetrix*: A long-term study (1986–2019) in the French Alps. Research in Veterinary Science 137: 163–169. 10.1016/j.rvsc.2021.05.005 33989963

[B156] Türk R (1858) Ueber die in Oesterreich unter der Enns bis jetzt aufgefundenen Orthopteren. Wiener Entomologische Monatschrift 2(12): 361–381. https://www.zobodat.at/pdf/WEMS_2_0361-0381.pdf

[B157] Türk R (1860) Mehrere für Niederösterreichs Fauna neue Orthopteren. Wiener Entomologische Monatsschrift 4: 84–88. https://www.zobodat.at/pdf/WEMS_4_0084-0088.pdf

[B158] Türk R (1862) Für Niederösterreichs Fauna neue Orthopteren. Wiener Entomologische Monatsschrift 6: 81–82. https://www.zobodat.at/pdf/WEMS_6_0081-0082.pdf

[B159] Tyler MJ Fucsko L Roberts D (2023) Calamities causing loss of museum collections: A historical and global perspective on museum disasters. Zootaxa 5230(2): 153–178. 10.11646/zootaxa.5230.2.2 37044851

[B160] van den Elzen R Berg H-M Pilat M Renner SC (2023) Type specimens of Thamnophilidae Swainson, 1824 (Chordata, Animalia) in the Bird Collection of the Natural History Museum Vienna. Annalen des Naturhistorischen Museums in Wien 125B: 45–81. https://www.zobodat.at/pdf/ANNA_125B_0045-0081.pdf

[B161] van der Laan R Fricke R Eschmeyer WN [Eds] (2023) Eschmeyer’s Catalog of Fishes: classification. http://www.calacademy.org/scientists/catalog-of-fishes-classification/ [Electronic version accessed 7 October 2023]

[B162] van der Meij SET Moolenbeek RG Hoeksema BW (2009) Decline of the Jakarta Bay molluscan fauna linked to human impact. Marine Pollution Bulletin 59(4–7): 101–107. 10.1016/j.marpolbul.2009.02.021 19342065

[B163] van der Meij SET Suharsono Hoeksema BW (2010) Long-term changes in coral assemblages under natural and anthropogenic stress in Jakarta Bay (1920–2005). Marine Pollution Bulletin 60(9): 1442–1454. 10.1016/j.marpolbul.2010.05.011 20615515

[B164] van Steenberge M Snoeks J Vreven E (2016) Lingering taxonomic confusion in *Labeo* (Actinopterygii: Cypriniformes: Cyprinidae): correcting the records and basis of type designations for seven Congolese species. Acta Ichthyologica et Piscatoria 46(1): 1–8. 10.3750/AIP2016.46.1.01

[B165] Verhoeff KW (1891) Ein Beitrag zur mitteleuropäischen Diplopodenfauna. Berliner Entomologische Zeitschrift 36(1): 115–166. 10.1002/mmnd.18910360114

[B166] Verhoeff KW (1895) Beiträge zur Kenntnis paläarktischer Myriopoden. I. Aufsatz: Über einige neue Myriopoden der österreichisch-ungarischen Monarchie. Verhandlungen der Zoologisch-botanischen Gesellschaft in Wien 45: 284–298. https://www.zobodat.at/pdf/VZBG_45_0284-0298.pdf

[B167] Verhoeff KW (1907) Über Diplopoden. 7. (27.) Aufsatz: Europäische Polydesmiden. Zoologischer Anzeiger 32(12–13): 337–354.

[B168] Verhoeff KW (1928) Ueber Diplopoden aus Bulgarien gesammelt von Dr. I. Buresch. 3 Aufsatz in Dr. Karl W. Verhoeff in Pasing bein München. Band 1, 28–44.

[B169] Verhoeff KW (1930a) Über Diplopoden aus Italien, namentlich Piemont. 114. Diplopoden-Aufsatz. Zoologische Jahrbucher. Abteilung fur Systematik, Ökologie und Geographie der Tiere 59: 387–446.

[B170] Verhoeff KW (1930b) Zur Kenntnis italienischer Diplopoden. 119. Diplopoden-Aufsatz. Zoologische Jahrbucher. Abteilung fur Systematik, Ökologie und Geographie der Tiere 60: 281–326.

[B171] Verhoeff KW (1932) Zur Geographie, Ökologie und Systematik der Diplopoden Nordwestitaliens. Archiv für Naturgeschichte 1: 517–645.

[B172] Verhoeff KW (1941) Diplopoden der Insel Ischia, systematisch, morphologisch, phänologisch, ökologisch, geographisch. Zoomorphology 38(1): 147–196. 10.1007/BF02176181

[B173] Verhoeff KW (1942) Diplopoden der Insel Kapri. Zoologischer Anzeiger 139: 213–233.

[B174] Verhoeff KW (1951) Diplopoda, Chilopoda und Isopoda terrestria vom Mt. Soratte in Latium. Zoologische Jahrbucher. Abteilung fur Systematik, Ökologie und Geographie der Tiere 80: 205–255.

[B175] Verhoeff KW (1952) Weitere Beiträge zur Kenntnis der Isopoden- und Diplopodenfauna von Ischia und Capri. Bonner Zoologische Beitrage 3(1–2): 125–150.

[B176] von Meidinger C (1786) Icones piscium Austriae indigenorum, quos collegit vivisque coloribus espresos edidit C. Baron de Meidinger. Tafeln von J. Lachenbauer und M. Sedelmayer. Vol. 2: Plates X–XX. Baumeister Press, Vienna.

[B177] von Meidinger C (1790) Icones piscium Austriae indigenorum, quos collegit vivisque coloribus espresos edidit C. Baron de Meidinger. Tafeln von J. Lachenbauer und M. Sedelmayer. Vol. 4: Plates XXXI–XL. Baumeister Press, Vienna.

[B178] von Wurzbach C (1862) Heckel, Johann Jacob. In: Biographisches Lexikon des Kaiserthums Oesterreich. 8. Theil. Kaiserlich-königliche Hof- und Staatsdruckerei, Vienna, 184–189.

[B179] von Wurzbach C (1864) Kollar, Vincenz. In: Biographisches Lexikon des Kaiserthums Oesterreich. 12. Theil. Kaiserlich-königliche Hof- und Staatsdruckerei, Vienna, 333–338.

[B180] Webster GA (1959) *Orchipedumtracheicola* reported from a whistling swan, *Cygnuscolumbianus*. Canadian Journal of Zoology 37(2): 213–213. 10.1139/z59-023

[B181] Webster MS [Ed.] (2017) The Extended Specimen: Emerging Frontiers in Collections-Based Ornithological Research. Studies in Avian Biology Series, No. 50. CRC Press, Taylor and Francis Group, Boca Raton, FL, USA, 240 pp. 10.1201/9781315120454

[B182] Wehr EE Dikmans G (1935) New nematodes (Filariidae) from North American Ruminants. Zoologischer Anzeiger 110: 202–208.

[B183] Winston JE (1999) Describing species: Practical taxonomic procedure for biologists. Columbia University Press, 512 pp. https://www.jstor.org/stable/10.7312/wins06824

[B184] Wirkner C Stagl V Turk N (2002) Type specimens of the Chordeumatida in the Natural History Museum (Diplopoda). Kataloge der wissenschaftlichen Sammlungen des Naturhistorischen Museums in Wien, Band 16 Myriapoda, Heft 1.

[B185] Wolfram G Mikschi E (2007) Rote Liste der Fische (Pisces) Österreichs. Rote Listen gefährdeter Tiere Österreichs, Teil 2: Kriechtiere, Lurche, Fische, Nachtfalter, Weichtiere. Böhlau Verlag, Wien-Köln-Weimar, 515 pp.

[B186] Zahiri R Tarmann G Efetov KA Rajaei H Fatahi M Seidel M Jaenicke B Dalsgaard T Sikora M Husemann M (2021) An illustrated catalogue of the type specimens of Lepidoptera (Insecta) housed in the Zoological Museum Hamburg (ZMH): Part I. superfamilies Hepialoidea, Cossoidea, and Zygaenoidea. Evolutionary Systematics 5(1): 39–70. 10.3897/evolsyst.5.62003

[B187] Zangl L Schäffer S Daill D Friedrich T Gessl W Mladinić M Sturmbauer C Wanzenböck J Weiss SJ Koblmüller S (2022) A comprehensive DNA barcode inventory of Austria’s fish species. PLOS ONE 17(6): e0268694. 10.1371/journal.pone.0268694 PMC9182252 35679240

[B188] Zettel H Laciny A Bruckner H (2022) Catalogue of the type specimens of the family Gerridae (Insecta: Hemiptera: Heteroptera) in the Natural History Museum Vienna. Annalen des Naturhistorischen Museums in Wien 124B: 193–247. https://www.zobodat.at/pdf/ANNA_124B_0193-0247.pdf

[B189] Zettel H Bruckner H Laciny A Zenz K (2023) Catalogue of the type specimens of the family Hebridae (Insecta: Hemiptera: Heteroptera) in the Natural History Museum Vienna. Annalen des Naturhistorischen Museums in Wien 125B: 13–36. https://www.zobodat.at/pdf/ANNA_125B_0013-0036.pdf

[B190] Zuna-Kratky T (2017) Zur Geschichte der Heuschreckenforschung in Österreich. Denisia 39: 35–54. https://www.zobodat.at/pdf/DENISIA_0039_0035-0054.pdf

[B191] Zuna-Kratky T Karner-Ranner E Lederer E Braun B Berg H-M Denner M Bieringer G Ranner A Zechner L (2009) Verbreitungsatlas der Heuschrecken und Fangschrecken Ostösterreichs. Naturhistorisches Museum Wien, Vienna, 303 pp.

[B192] Zuna-Kratky T Landmann A Illich I Zechner L Essl F Lechner K Ortner A Weißmair W Wöss G (2017) Die Heuschrecken Österreichs. Denisia 39: 1–880.

